# Diazo compounds for the bioreversible esterification of proteins†
†Electronic supplementary information (ESI) available: Experimental procedures, analytical data, and spectral data for novel compounds. See DOI: 10.1039/c4sc01768d



**DOI:** 10.1039/c4sc01768d

**Published:** 2014-10-01

**Authors:** Nicholas A. McGrath, Kristen A. Andersen, Amy K. F. Davis, Jo E. Lomax, Ronald T. Raines

**Affiliations:** a Department of Chemistry , University of Wisconsin–Madison , 1101 University Avenue , Madison , WI 53706 , USA . Email: rtraines@wisc.edu; b Molecular & Cellular Pharmacology Graduate Training Program , University of Wisconsin–Madison , 1300 University Avenue , Madison , WI 53706 , USA; c Department of Biochemistry , University of Wisconsin–Madison , 433 Babcock Drive , Madison , WI 53706 , USA; d Graduate Program in Cellular and Molecular Biology , University of Wisconsin–Madison , 1525 Linden Drive , Madison , WI 53706 , USA

## Abstract

A diazo compound is shown to convert carboxylic acids to esters efficiently in an aqueous environment. The basicity of the diazo compound is critical: low basicity does not lead to a reaction but high basicity leads to hydrolysis. This reactivity extends to carboxylic acid groups in a protein. The ensuing esters are hydrolyzed by human cellular esterases to regenerate protein carboxyl groups. This new mode of chemical modification could enable the key advantages of prodrugs to be translated from small-molecules to proteins.

## Abstract 2

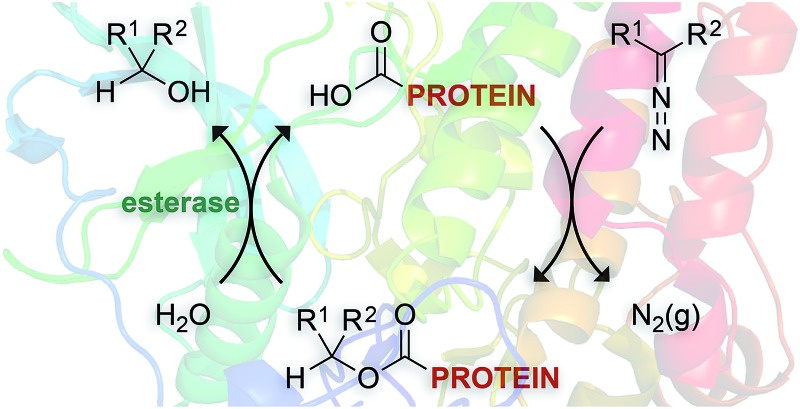
A diazo compound is shown to convert carboxylic acids to esters efficiently in an aqueous environment.

## Introduction

Esters are nearly absent from human cells, aside from acylglycerols and amino-acyl tRNAs. One reason is their lability in the presence of intracellular esterases.^1^ This reactivity is the basis for the clinical utility of the many prodrugs that are unmasked by enzyme-catalyzed ester hydrolysis.^2–5^

Inspired by prodrugs, we envisioned esterification as a means to modify proteins covalently but reversibly with desirable pendants, such as pharmacokinetic enhancing, cell-targeting, or cell-penetration moieties. The acetylation of serine and threonine residues is a natural post-translational modification.^6,7^ There is not, however, an efficient means to generate esters from protein carboxyl or hydroxyl groups by chemical synthesis. Carbodimides and other reagents can be used to activate protein carboxyl groups, but solvent water and protein amino, hydroxyl, and sulfhydryl groups compete effectively with exogenous alcohols for the ensuing activated acyl groups.^8–10^ Accordingly, we sought a different strategy—one that enlists *O*-alkylation rather than acyl transfer.

Diazo compounds are in widespread use in synthetic organic chemistry.^11–13^ The simplest—diazomethane—readily converts carboxylic acids into methyl esters with molecular nitrogen as the only byproduct. Diazomethane suffers, however, from non-specific reactivity. For example, diazomethane is known to react with water and with the side chains of lysine and tyrosine residues.^14^ Other non-stabilized diazo compounds or stabilized metal carbenoids are capable of esterifying carboxylic acids,^15–20^ but their high reactivity likewise limits utility with biomolecules.

We were aware of precedent for the use of stabilized diazo compounds in a biochemical context. In the 1960s, 2-diazoacetamide,^21,22^ *N*-(diazoacetyl)glycinamide,^23^ and diphenyldiazomethane^24^ were used to identify the most reactive carboxylic acid groups in proteins. These efforts required adding a vast molar excess (>10^3^-fold) of the diazo compound and tedious monitoring of reaction pH, all to achieve modest labelling. We suspected that the problem was non-specific reactivity, as with diazomethane.^14^ Here, we report on the reactivity of still more stabilized diazo compounds with relevant carboxylic acids.

## Results and discussion

**Scheme 1:**
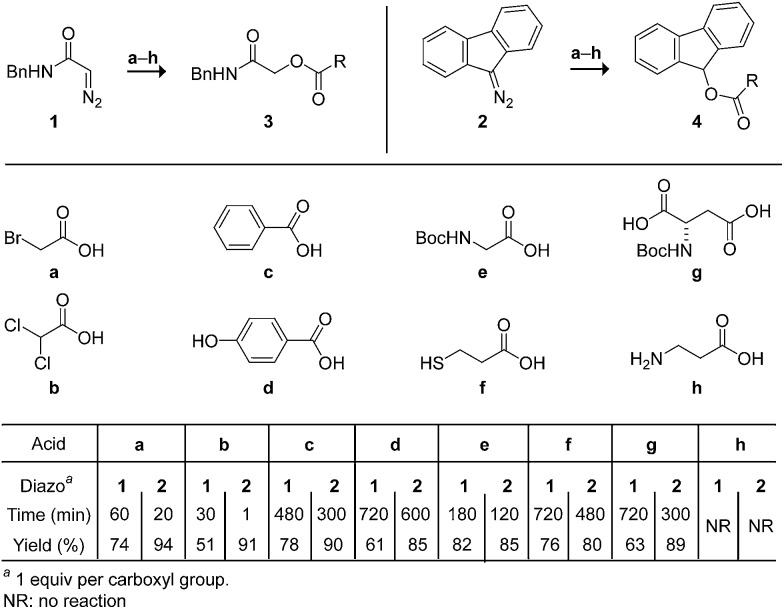
Chemoselective esterification in acetonitrile.

**Fig. 1:**
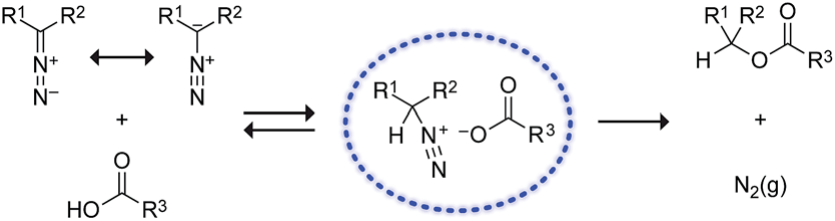
Putative mechanism for the *O*-alkylation of carboxylic acids by diazo compounds.^27,28^

We began by screening the reactivity of diazo compounds **1** and **2** with small-molecule carboxylic acids of varying acidity and bearing a variety of reactive functional groups. These diazo compounds were accessed from their corresponding azide by a deimidogenation reaction that uses a phosphinoester to convert an azide into a diazo-compound.^25,26^ In acetonitrile, both diazo compounds were unreactive toward some nucleophiles common in biomolecules: amino, hydroxyl, and sulfhydryl groups. Both, however, did react with carboxylic acids to give comparable yields of ester product (Scheme 1). In contrast to diazo compounds **1** and **2**, diethyl 2-diazomalonate was unreactive with **a–h**. Tellingly, β-alanine (**h**) proved unreactive with all three diazo compounds under any tested condition. This acid alone exists as a zwitterion, and its lack of reactivity is consistent with esterification occurring *via* a diazonium-carboxylate salt (Fig. 1).^27,28^ Moreover, we noted that a carboxylic acid (**a–g**) is acidic enough to promote the reaction, but a phenolic hydroxyl (**d**), sulfhydryl (**f**), or ammonium group (**h**) is not.

**Scheme 2:**
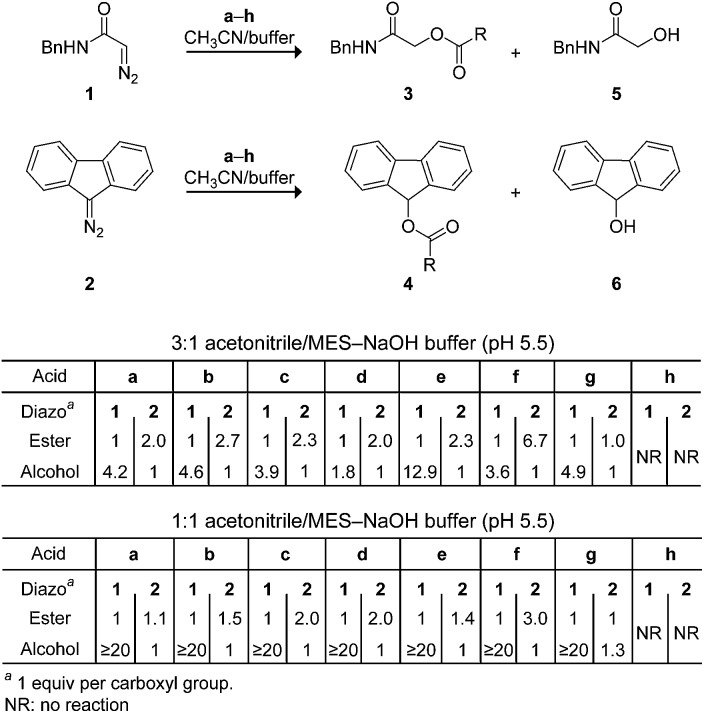
Chemoselective esterification in an aqueous environment.

Next, we investigated the analogous reactions in an aqueous environment (Scheme 2). Whereas diazo compound **1** was competent for esterification in a 3 : 1 mixture of acetonitrile and 10 mM MES–NaOH buffer, pH 5.5, the major product was alcohol **5** formed when water attacks the diazonium ion. On the other hand, diazo compound **2** gave primarily the desired ester **4** in all cases. Interestingly, the ester : alcohol product ratios with **1** were variable, whereas those with **2** were approximately two.^29^ These data are consistent with the nascent diazonium-carboxylate salt being maintained in a solvent cage by Coulombic forces (Fig. 1), as described by others.^28,30^ Carrying out the reaction in a 1 : 1 mixture of acetonitrile and 10 mM MES–NaOH buffer resulted in greatly deleterious partitioning with **1** but not **2**. Diethyl 2-diazomalonate was found to be unreactive in these aqueous conditions, as in neat acetonitrile.

What is the basis for this differential reactivity? The p*K*_a_ values of relevant acids have been measured in dimethyl sulfoxide.^31^ These p*K*_a_ values correlate with the observed reactivity *via* the mechanism of Fig. 1. Specifically, the conjugate base of diethylmalonate (p*K*_a_ 16.4)^32^ is weak, and diethyl 2-diazomalonate cannot abstract a proton from a carboxylic acid. On the other hand, the conjugate base of diethylacetamide (p*K*_a_ 35)^33^ is strong, and diazo compound **1** lacks chemoselectivity in an aqueous environment (Scheme 2). Only the conjugate base of fluorene (p*K*_a_ 22.6)^34^ is matched to the task, enabling diazo compound **2** access to the mechanism of Fig. 1 with high chemoselectivity.

**Scheme 3:**
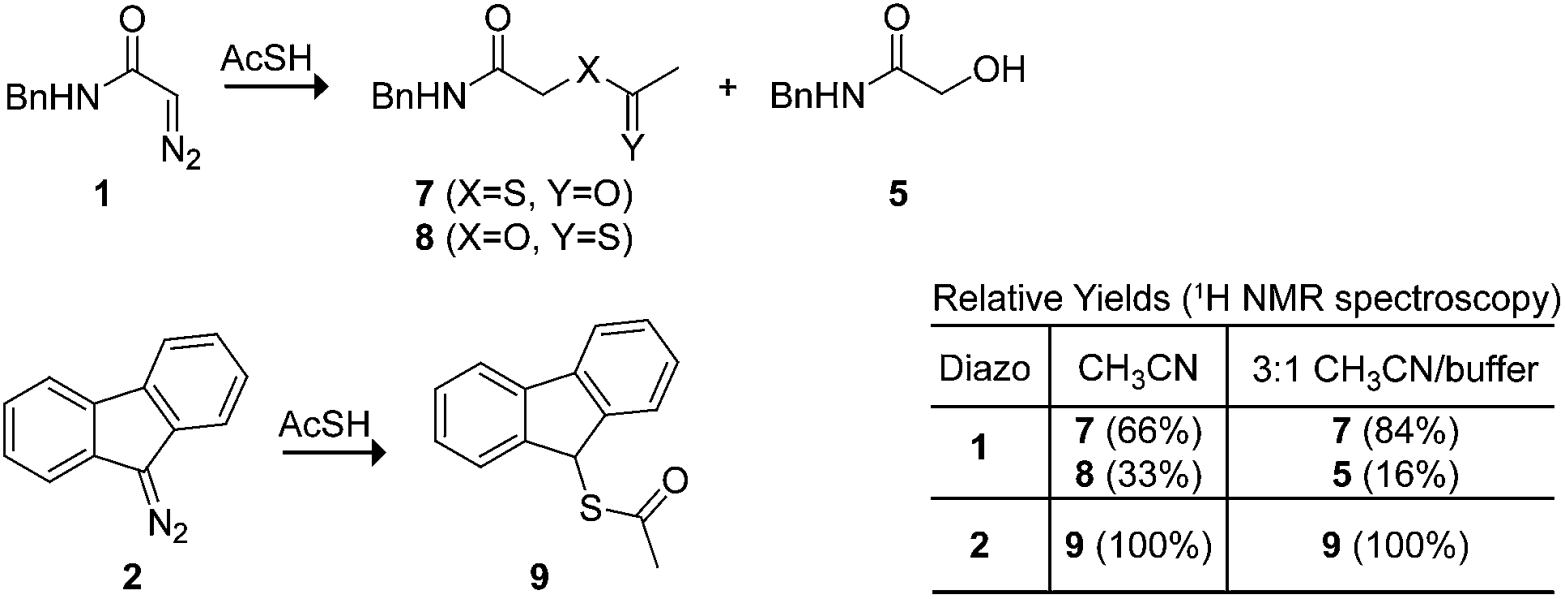
Thio- *versus* thionoester formation.

We used thioacetic acid to probe the mechanism of esterification. Its acidic proton resides primarily on sulfur,^35^ but an intermediate diazonium-thiocarboxylate salt could lead to either a thioester or thionoester product. In anhydrous acetonitrile, complete selectivity for thioester formation was obtained with diazo compound **2** (Scheme 3). This selectivity rules out a cyclic transition state reminiscent of an ene reaction,^36,37^ which would provide the thionoester as the product. On the other hand, diazo compound **1** produced a mixture of thio- and thionoester products. In an aqueous environment, these same reactants led exclusively to a thio- rather than a thionoester, along with the hydrolysis product from diazo compound **1**.^38^ The lack of thionoester formation can be attributed to the greater nucleophilicity of sulfur on the diazonium salt and the greater ability of oxygen to form a hydrogen bond with solvent water,^39^ which would decrease its nucleophilicity still further.

**Fig. 2:**
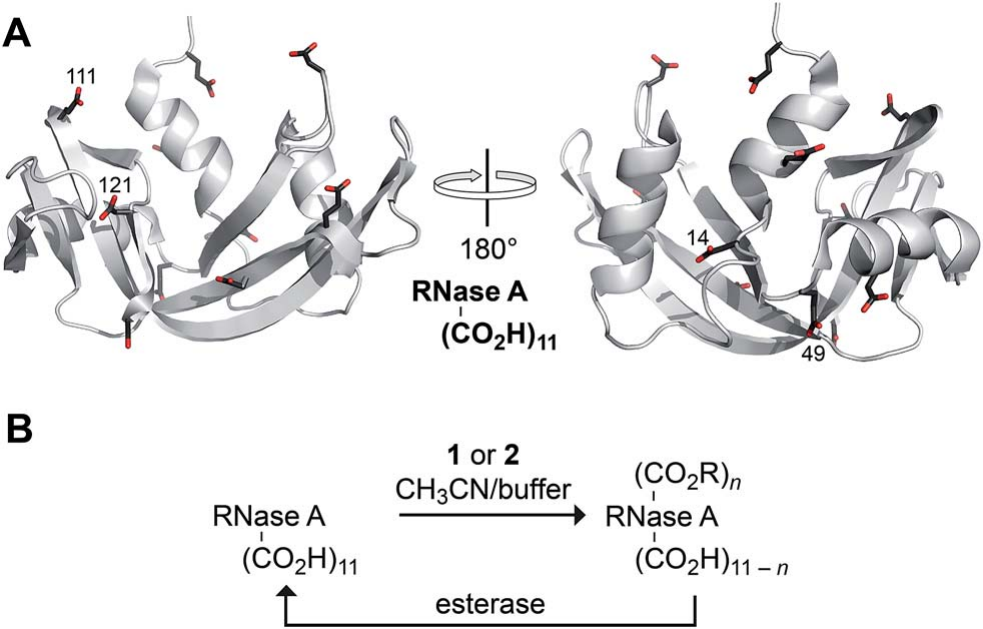
(A) Three-dimensional structure of RNase A (PDB entry 6rsa) showing its eleven carboxyl groups. The four residues labeled primarily by diazo compound **2** are indicated explicitly. (B) Scheme for the bioreversible esterification of RNase A with diazo compound **2**.

Finally, we assessed the ability of diazo compounds **1** and **2** to esterify the carboxyl groups present in a model protein, bovine pancreatic ribonuclease (RNase A; Fig. 2A). The catalytic activity of this well-characterized enzyme provides an extremely sensitive measure of native structure and function.^40^ RNase A was incubated for 4 h at 37 °C in a 1 : 1 mixture of 10 mM MES–NaOH buffer, pH 5.5, and acetonitrile containing a diazo compound (10 equiv.). Under these conditions, diazo compound **1** proved incapable of labelling the protein. Only in the presence of 200 equiv. was any esterification observed with this reagent, consistent with its tendency towards hydrolysis in an aqueous environment (Scheme 2). In contrast, diazo compound **2** esterified, on average, three of the eleven carboxylates (Fig. S1†). Using trypsin digestion coupled with mass spectrometry, we were able to identify the esterified residues. Diazo compound **2** (10 equiv.) labelled Asp14, Glu49, Glu111, and Asp121, almost exclusively. Diazo compound **1** (200 equiv.) labelled a similar subset of residues (Glu9, Asp14, Glu49, Glu111, and the C terminus). The basis for reaction with these particular residues is not clear, but likely integrates the reactivity (*i.e.*, high p*K*_a_) and accessibility of their carboxyl groups.

Most importantly, we probed the bioreversibility of our protein esterification reaction (Fig. 2B). To enhance the relevance of this experiment, we sought to employ esterases endogenous to human cells rather than commercial enzymes. Accordingly, we used recombinant DNA technology to add an 8-residue FLAG tag to the N-terminus of RNase A, and we esterified the ensuing FLAG–RNase A with diazo compound **2** as described above. We found labelling to be comparable to that of wild-type RNase A (Fig. S12†), and we observed that the enzyme lost half of its catalytic activity upon esterification (Fig. S13†).^41^ Then, we incubated the esterified protein with a HeLa cell extract. We recovered the enzyme from this complex mixture with an anti-FLAG antibody immobilized on magnetic beads. We could not detect any esters in the extract-treated enzyme with mass spectrometry (Fig. S12†),^42^ and we found that the enzyme had recovered all of its catalytic activity (Fig. S13†). These data indicate that the protein esterification reaction was fully bioreversible.

Finally, we sought to demonstrate the generality of our esterification method by employing a second protein substrate with a different tag for its purification. Specifically, we applied diazo compound **2** (10 equiv.) and the conditions described above to the mCherry variant of red fluorescent protein (RFP) from *Discosoma* sp. We found that His_6_–RFP became esterified with 1–3 fluorenyl groups. The protein did not lose its fluorescence during the esterification reaction. As with RNase A, the esterification of RFP was bioreversible. Incubation with a HeLa cell extract followed by recovery with a Ni-affinity resin, yielded unmodified His_6_–RFP (Fig. S13†). Success with both FLAG-labeled RNase A and His_6_-labeled RFP indicates that our esterification method has a broad scope.

## Concluding remarks

We find that a diazo compound can effect the efficient *O*-alkylation of carboxylic acids in an aqueous environment near neutral pH, and that this reactivity extends to proteins. The esters in the ensuing protein can be hydrolysed by esterases that are endogenous to human cells, thereby recreating the wild-type protein and avoiding a compromise to function or the display of an epitope. By enabling the facile semisynthesis of “proproteins” containing transitory pharmacokinetic enhancing, cell-targeting, or cell-penetration moieties, we envision that *O*-alkylation of carboxylic acids by diazo compounds could broaden the utility of antibodies, enzymes, hormones, and other proteins in chemical biology and pharmacology.
