# Supplementary material for: Diazo compounds for the bioreversible esterification of proteins
Source: Chem Sci. 2014 Oct 1;6(1):752–5. doi: 10.1039/c4sc01768d (PMC4275067; doi:10.1039/c4sc01768d)
Supplement: Supplementary file 1 [file SC-006-C4SC01768D-s001.pdf]

Electronic Supplementary Material (ESI) for Chemical Science.
This journal is © The Royal Society of Chemistry 2014

Diazo compounds for the bioreversible esterification of proteins

Nicholas A. McGrath, Kristen A. Andersen, Amy K. F. Davis, Jo E. Lomax, and Ronald T. Raines*

Table of Contents

1. General Methods S1
2. Experimental Procedures and Characterization Data S2
   A. Esterification Reactions in Acetonitrile S2
      I. Diazoacetamide Reactions S2
      II. Diazofluorene Reactions S4
   B. Esterification Screening in Acetonitrile/Buffer Solution S6
   C. Thioester versus Thionoester Formation with Thioacetic Acid S7
3. RNase A-Esterification Experiments S8
4. Trypsin-Digestion and MS/MS Experiments to Identify Esterified Carboxyl Groups in RNase A S8
5. FLAG–RNase A-Esterification Experiments S9
6. His_6–RFP-Esterification Experiments S9
7. MALDI–TOF Mass Spectrometry Data for RNase A-Esterification Experiments S11
8. MS/MS Data for Trypsin-Digestion Experiments S12
9. MALDI–TOF Mass Spectrometry Data for FLAG–RNase A-Esterification Experiments S21
10. Ribonucleolytic Activity Assay Data S23
11. MALDI–TOF Mass Spectrometry Data for His_6–RFP-Esterification Experiments S24
12. References S24
13. NMR Spectra S25

1. General Methods

Reagent chemicals were obtained from commercial sources and used without further purification. All glassware was flame-dried under high vacuum, and reactions were performed under N_2(g) unless indicated otherwise. Dichloromethane, diethyl ether, tetrahydrofuran, and toluene were dried over a column of alumina. Dimethylformamide and triethylamine were dried over alumina and purified further by passage through an isocyanate scrubbing column. Flash chromatography was performed with columns of 40–63 Å silica gel, 230–400 mesh from Silicycle (Québec City, Canada). Thin-layer chromatography (TLC) was performed on plates of EMD 250-μm silica 60-F_{254}.

The phrase "concentrated under reduced pressure" refers to the removal of solvents and other volatile materials using a rotary evaporator at water aspirator pressure (<20 torr) while maintaining the water-bath temperature below 40 °C. Residual solvent was removed from samples at high vacuum (<0.1 torr). The term "high vacuum" refers to vacuum achieved by mechanical belt-drive oil pump.

All NMR spectra were acquired at ambient temperature with a DMX-400 Avance, Avance III 500i with cryoprobe, or Avance III 500ii with cryoprobe spectrometer from Bruker (Billerica, MA) at the National Magnetic Resonance Facility at Madison (NMRFAM), and were referenced to TMS or a residual protic solvent.

Electrospray ionization (ESI) mass spectrometry for small-molecule characterization was performed with a Micromass LCT at the Mass Spectrometry Facility in the Department of Chemistry at the University of Wisconsin–Madison. Matrix-assisted laser desorption ionization–time-of-flight (MALDI–TOF) mass spectrometry for protein characterization was performed with a Voyager DE-Pro instrument at the Biophysics Instrumentation Facility at the University of Wisconsin–Madison.

Cell culture. HeLa cells were obtained from ATCC (Manassas, VA). Cells were cultured in DMEM supplemented with FBS (10% v/v) and penicillin/streptomycin (1% w/v) at 37 °C in the presence of 5% CO_2(g).

2. Experimental Procedures and Characterization Data

A. Esterification Reactions in Acetonitrile
I. Diazobenzylacetamide Reactions

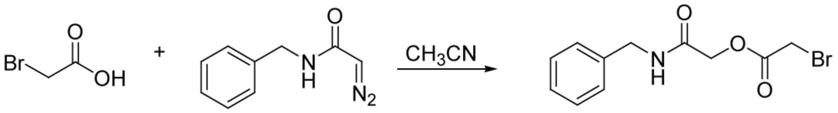

Diazobenzylacetamide (0.010 g, 0.057 mmol) was added to a solution of bromoacetic acid (0.008 g, 0.057 mmol) in anhydrous acetonitrile (0.57 mL), and the reaction mixture was stirred for 1 h, when the reaction was determined to be complete by thin-layer chromatography (R_f = 0.3 in 50% EtOAc, 50% hexanes). The reaction mixture was concentrated under reduced pressure and purified by silica gel chromatography to give *N*-benzyl-acetamido-bromoacetate (0.012 g, 74%).

^{1}H NMR (400 MHz, CDCl$_3$) δ 7.38–7.16 (m, 5H), 6.41 (bs, 1H), 4.70 (s, 2H), 4.50 (d, J = 5.9 Hz, 2H), 3.87 (s, 2H). **^{13}C NMR (126 MHz, CDCl$_3$)** δ 166.3, 165.9, 137.5, 129.1, 129.0, 128.0, 78.0, 64.1, 43.5, 25.2. **HRMS** (ESI) *m/z* 286.0074 [calc'd for $C_{11}H_{13}BrNO_3$ (M+H$^+$) 286.0074].

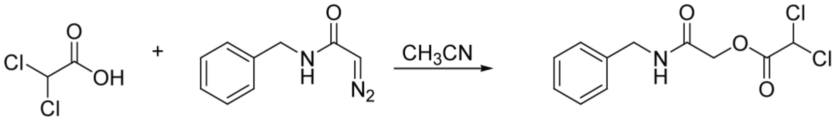

Diazobenzylacetamide (0.010 g, 0.057 mmol) was added to a solution of dichloroacetic acid (0.005 mL, 0.057 mmol) in anhydrous acetonitrile (0.57 mL), and the reaction mixture was stirred for 30 min, when the reaction was determined to be complete by thin-layer chromatography (R_f = 0.3 in 30% EtOAc, 70% hexanes). The reaction mixture was concentrated under reduced pressure and purified by silica gel chromatography to give benzyl-acetamido-dichloroacetate (0.008 g, 51%)

^{1}H NMR (500 MHz, CDCl$_3$) δ 7.43–7.29 (m, 5H), 6.38 (bs, 1H), 6.05 (s, 1H), 4.83 (s, 2H), 4.56 (d, J = 5.8 Hz, 2H). **^{13}C NMR (126 MHz, CDCl$_3$)** δ 165.5, 163.1, 137.3, 129.1, 128.1, 128.0, 64.9, 63.9, 43.6. **HRMS** (ESI) *m/z* 293.0459 [calc'd for $C_{11}H_{15}Cl_2N_2O_3$ (M+NH$_4^+$) 293.0455].

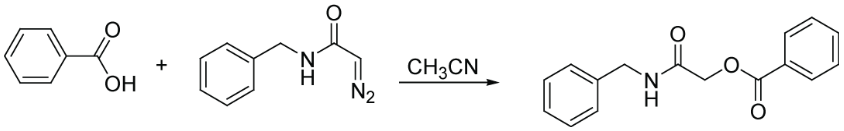

Diazobenzylacetamide (0.010 g, 0.057 mmol) was added to a solution of benzoic acid (0.007 g, 0.057 mmol) in anhydrous acetonitrile (0.57 mL), and the reaction mixure was stirred for 8 h, when the reaction was determined to be complete by thin-layer chromatography (R_f = 0.8 in 80% EtOAc, 20% hexanes). The reaction mixture was concentrated under reduced pressure and purified by silica gel chromatography to give benzyl-acetamido-benzoate (0.012 g, 78%).

^{1}H NMR (400 MHz, CDCl$_3$) δ 8.02 (d, J = 7.6 Hz, 2H), 7.59 (t, J = 7.3 Hz, 1H), 7.45 (t, J = 7.6 Hz, 2H), 7.39–7.21 (m, 5H), 6.42 (bs, 1H), 4.87 (s, 2H), 4.53 (d, J = 6.0 Hz, 2H). **^{13}C NMR (126 MHz, CDCl$_3$)** δ 167.3, 165.4, 137.8, 134.0, 134.0, 130.0, 129.0, 128.9, 127.9, 127.9, 63.7, 43.3. **HRMS** (ESI) *m/z* 270.1133 [calc'd for $C_{16}H_{16}NO_3$ (M+H$^+$) 270.1125].

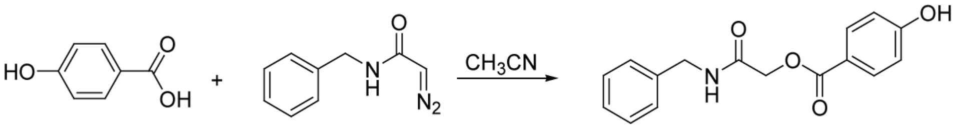

Diazobenzylacetamide (0.010 g, 0.057 mmol) was added to a solution of 4-hydroxybenzoic acid (0.008 g, 0.057 mmol) in anhydrous acetonitrile (0.57 mL), and the reaction mixture was stirred for 12 h, when the reaction was determined to be complete by thin-layer chromatography (R_f = 0.6 in 75% EtOAc, 25% hexanes). The reaction mixture was concentrated under reduced pressure and purified by silica gel chromatography to give benzyl-acetamido-4-hydroxybenzoate (0.011 g, 61%).

^{1}H NMR (700 MHz, $CDCl_3$) *δ* 7.98 (d, *J* = 8.7 Hz, 2H), 7.40–7.35 (m, 2H), 7.36–7.31 (m, 3H), 6.90 (d, *J* = 8.7 Hz, 2H), 6.45 (bs, 1H), 4.89 (s, 2H), 4.58 (d, *J* = 5.9 Hz, 2H). **^{13}C NMR (126 MHz, $CDCl_3$)** *δ* 167.6, 165.0, 160.5, 137.8, 132.4, 129.0, 127.9, 127.9, 121.6, 115.7, 63.5, 43.3. **HRMS** (ESI) *m/z* 286.1070 [calc'd for $C_{16}H_{16}NO_4$ ($M+H^+$) 286.1074].

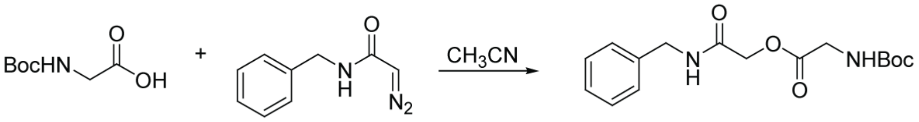

Diazobenzylacetamide (0.010 g, 0.057 mmol) was added to a solution of Boc-protected glycine (0.010 g, 0.057 mmol) in anhydrous acetonitrile (0.57 mL), and the reaction mixture was stirred for 3 h, when the reaction was determined to be complete by thin-layer chromatography (R_f = 0.7 in 75% EtOAc, 25% hexanes). The reaction mixture was concentrated under reduced pressure and purified by silica gel chromatography to give benzyl-acetamido-Boc-protected glycine (0.015 g, 82%).

^{1}H NMR (400 MHz, $CDCl_3$) *δ* 7.38–7.25 (m, 5H), 7.07 (bs, 1H), 5.10 (bs, 1H), 4.71 (s, 2H), 4.48 (d, *J* = 6.0 Hz, 2H), 3.90 (d, *J* = 5.9 Hz, 2H), 1.37 (s, 9H). **^{13}C NMR (126 MHz, $CDCl_3$)** *δ* 169.1, 166.8, 156.4, 137.8, 128.7, 127.8, 127.5, 80.8, 63.2, 43.1, 42.9, 28.2. **HRMS** (ESI) *m/z* 340.1873 [calc'd for $C_{16}H_{26}N_3O_5$ ($M+NH_4^+$) 340.1867].

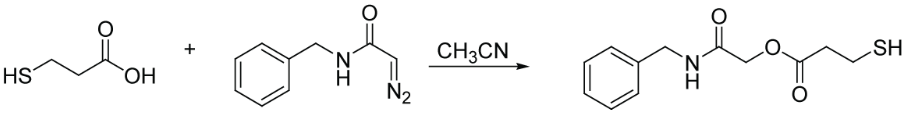

Diazobenzylacetamide (0.010 g, 0.057 mmol) was added to a solution of 3-mercaptopropanoic acid (0.005 mL, 0.057 mmol) in anhydrous acetonitrile (0.57 mL), and the reaction mixture was stirred for 12 h, when the reaction was determined to be complete by thin-layer chromatography (R_f = 0.6 in 70% EtOAc, 30% hexanes). The reaction mixture was concentrated under reduced pressure and purified by silica gel chromatography to give benzyl-acetamido-3-mercaptopropanoate (0.011 g, 76%).

^{1}H NMR (400 MHz, $CDCl_3$) *δ* 7.46–7.13 (m, 5H), 6.54 (bs, 1H), 4.69 (s, 2H), 4.50 (d, *J* = 5.7 Hz, 2H), 2.82–2.77 (m, 2H), 2.76–2.71 (m, 2H), 1.59 (t, *J* = 8.1 Hz, 1H). **^{13}C NMR (126 MHz, $CDCl_3$)** δ 170.5, 166.9, 137.7, 129.0, 128.1, 128.0, 63.4, 43.4, 38.3, 20.0. **HRMS** (ESI) *m/z* 271.1115 [calc'd for $C_{12}H_{19}N_2O_3S$ ($M+NH_4^+$) 271.1111].

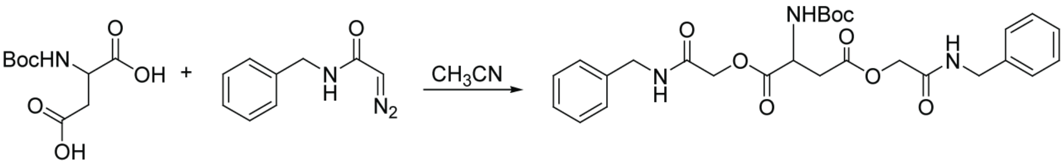

Diazobenzylacetamide (0.020 g, 0.114 mmol) was added to a solution of Boc-protected L-aspartic acid (0.013 g, 0.057 mmol) in anhydrous acetonitrile (0.57 mL), and the reaction mixture was stirred for 12 h, when the reaction was determined to be complete by thin-layer chromatography (R_f = 0.5 in 80% EtOAc, 20% hexanes). The reaction mixture was concentrated under reduced pressure and purified by silica gel chromatography to give bis-benzyl-acetamido-Boc-protected aspartate (0.019 g, 63%).

^{1}H NMR (400 MHz, $CDCl_3$) *δ* 7.38–7.20 (m, 10H), 7.04 (bs, 1H), 6.49 (bs, 1H), 5.53 (bs, 1H), 4.73–4.55 (m, 3H), 4.53–4.35 (m, 6H), 3.03 (dd, *J* = 16.9, 5.2 Hz, 1H), 2.94 (dd, *J* = 16.9, 5.2 Hz, 1H), 1.34 (s, 9H). **^{13}C NMR (126**

MHz, CDCl$_3$) δ 170.3, 170.3, 166.5, 166.2, 155.7, 137.7, 137.5, 128.8, 128.6, 127.9, 127.8, 127.8, 127.5, 81.1, 63.8, 63.4, 50.3, 43.2, 43.1, 36.4, 28.2. **HRMS** (ESI) *m/z* 545.2632 [calc'd for $C_{27}H_{37}N_4O_8$ ($M+NH_4^+$) 545.2606].

II. Diazofluorene Reactions

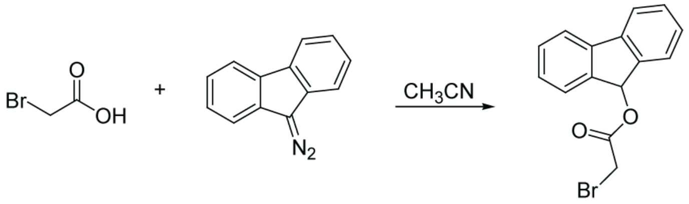

Diazofluorene (0.003 g, 0.016 mmol) was added to a solution of bromoacetic acid (0.002 g, 0.016 mmol) in anhydrous acetonitrile (0.16 mL), and the reaction mixture was stirred for 20 min, when the reaction was determined to be complete by thin-layer chromatography (R_f = 0.7 in 30% EtOAc, 70% hexanes). The reaction mixture was concentrated under reduced pressure and purified by silica gel chromatography to give fluorenyl-bromoacetate (0.004 g, 94%).

^{1}H NMR (400 MHz, CDCl$_3$) δ 7.66 (d, J = 7.5 Hz, 2H), 7.54 (d, J = 7.5 Hz, 2H), 7.41 (t, J = 7.5 Hz, 2H), 7.29 (t, J = 7.5 Hz, 2H), 6.80 (s, 1H), 3.92 (s, 2H). **^{13}C NMR (126 MHz, CDCl$_3$)** δ 168.4, 141.3, 141.3, 130.1, 128.2, 126.2, 120.4, 26.1. [Fluorenyl alkyl CH overlaps with a chloroform peak]. **^{13}C NMR (126 MHz, CD$_3$OD)** δ 170.1, 142.9, 142.5, 131.0, 129.2, 127.1, 121.3, 77.9, 26.7. **HRMS** (EI) *m/z* 301.9926 [calc'd for $C_{15}H_{11}BrO_2$ (M^+) 301.9937].

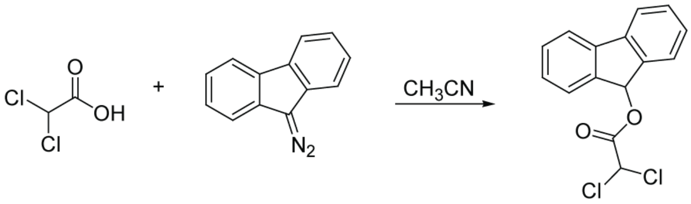

Diazofluorene (0.003 g, 0.016 mmol) was added to a solution of dichloroacetic acid (0.002 g, 0.016 mmol) in anhydrous acetonitrile (0.16 mL), and the reaction mixture was stirred for 1 min, when the reaction was determined to be complete by thin-layer chromatography (R_f = 0.7 in 30% EtOAc, 70% hexanes). The reaction mixture was concentrated under reduced pressure and purified by silica gel chromatography to give fluorenyl-dichloroacetate (0.004 g, 91%).

^{1}H NMR (400 MHz, CDCl$_3$) δ 7.68 (d, J = 7.5 Hz, 2H), 7.56 (d, J = 7.5 Hz, 2H), 7.45 (t, J = 7.5 Hz, 2H), 7.32 (t, J = 7.5 Hz, 2H), 6.83 (s, 1H), 6.03 (s, 1H). **^{13}C NMR (126 MHz, CDCl$_3$)** δ 165.8, 141.4, 140.6, 130.3, 128.4, 126.2, 120.5, 78.1, 64.5. **HRMS** (EI) *m/z* 292.0042 [calc'd for $C_{15}H_{10}Cl_2O_2$ (M^+) 292.0053].

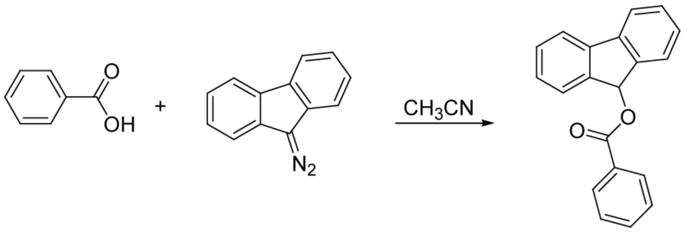

Diazofluorene (0.006 g, 0.031 mmol) was added to a solution of benzoic acid (0.004 g, 0.031 mmol) in anhydrous acetonitrile (0.31 mL), and the reaction mixture was stirred for 5 h, when the reaction was determined to be complete by thin-layer chromatography (R_f = 0.9 in 30% EtOAc, 70% hexanes). The reaction mixture was concentrated under reduced pressure and purified by silica gel chromatography to give fluorenyl-benzoate (0.008 g, 90%).

^{1}H NMR (400 MHz, $CDCl_3$) δ 8.09 (d, J = 7.5 Hz, 2H), 7.71 (d, J = 7.5 Hz, 2H), 7.63 (d, J = 7.5 Hz, 2H), 7.57 (t, J = 7.7Hz, 1H), 7.43 (t, J = 7.7 Hz, 4H), 7.31 (t, J = 7.5 Hz, 2H), 7.05 (s, 1H). **^{13}C NMR (101 MHz, $CDCl_3$)** δ 167.5, 142.4, 141.3, 133.4, 130.2, 130.2, 129.7, 128.6, 128.1, 126.3, 120.3, 75.8. **HRMS** (ESI) m/z 304.1338 [calc'd for $C_{20}H_{18}NO_2$ (M + NH_4^+) 304.1333].

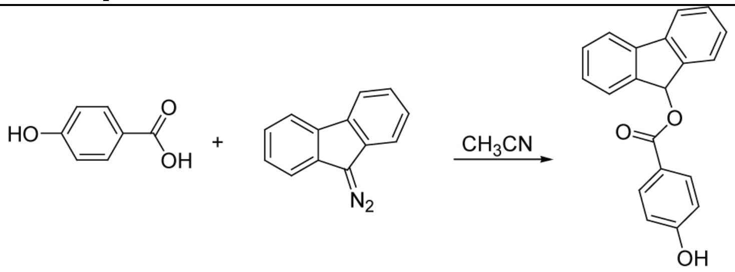

Diazofluorene (0.012 g, 0.063 mmol) was added to a solution of 4-hydroxybenzoic acid (0.009 g, 0.063 mmol) in anhydrous acetonitrile (0.60 mL), and the reaction mixture was stirred for 10 h before being concentrated under reduced pressure and the resulting residue was purified by silica gel chromatography to give fluorenyl-4-hydroxybenzoate (0.016 g, 85%).

^{1}H NMR (400 MHz, $CDCl_3$) δ 7.98 (d, J = 8.6 Hz, 2H), 7.68 (d, J = 7.6 Hz, 2H), 7.60 (d, J = 7.6 Hz, 2H), 7.41 (t, J = 7.6 Hz, 2H), 7.28 (t, J = 7.6 Hz, 2H), 7.00 (s, 1H), 6.82 (d, J = 8.6 Hz, 2H). **^{13}C NMR (126 MHz, $CDCl_3$)** δ 167.1, 160.0, 142.5, 141.3, 132.6, 129.7, 128.1, 126.3, 122.9, 120.3, 115.4, 75.6. **HRMS** (ESI) m/z 320.1293 [calc'd for $C_{20}H_{18}NO_3$ (M+NH_4^+) 320.1282].

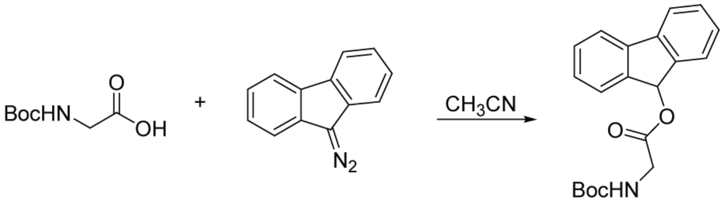

Diazofluorene (0.006 g, 0.031 mmol) was added to a solution of Boc-protected glycine (0.006 g, 0.031 mmol) in anhydrous acetonitrile (0.31 mL), and the reaction mixture was stirred for 2 h until determined to be complete by thin-layer chromatography (R_f = 0.6 in 30% EtOAc, 70% hexanes). The reaction mixture was concentrated under reduced pressure and purified by silica gel chromatography to give fluorenyl-Boc-protected glycine (0.009 g, 85%).

^{1}H NMR (400 MHz, $CDCl_3$) δ 7.65 (d, J = 7.5 Hz, 2H), 7.52 (d, J = 7.5 Hz, 2H), 7.40 (t, J = 7.5 Hz, 2H), 7.28 (t, J = 7.5 Hz, 2H), 6.81 (s, 1H), 5.03 (bs, 1H), 4.01 (d, J = 5.7 Hz, 2H), 1.44 (s, 9H). **^{13}C NMR (126 MHz, $CDCl_3$)** δ 171.4, 155.9, 141.6, 141.3, 130.0, 128.2, 126.2, 120.3, 80.4, 76.2, 43.0, 28.5. **HRMS** (ESI) m/z 340.1535 [calc'd for $C_{20}H_{22}NO_4$ (M+H^+) 340.1544].

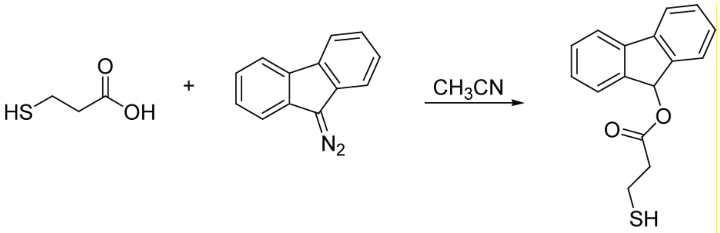

Diazofluorene (0.018 g, 0.094 mmol) was added to a solution of 3-mercaptopropanioc acid (0.010 g, 0.094 mmol) in anhydrous acetonitrile (0.94 mL), and the reaction mixture was stirred for 8 h before being concentrated under reduced pressure. The resulting residue was purified by silica gel chromatography to give fluorenyl-3-mercaptopropanoate (0.020 g, 80%).

^{1}H NMR (400 MHz, CDCl$_3$) δ 7.65 (d, *J* = 7.5 Hz, 2H), 7.53 (d, *J* = 7.5 Hz, 2H), 7.40 (t, *J* = 7.5 Hz, 2H), 7.28 (t, *J* = 7.5 Hz, 2H), 6.83 (s, 1H), 2.83 (dd, *J* = 8.2, 6.3 Hz, 2H), 2.76 (t, *J* = 6.3 Hz, 2H), 1.65 (t, *J* = 8.2 Hz, 1H). **^{13}C NMR (126 MHz, CDCl$_3$)** δ 172.6, 142.0, 141.3, 129.8, 128.1, 126.1, 120.3, 75.6, 39.0, 20.2. **HRMS** (ESI) *m*/*z* 288.1059 [calc'd for $C_{16}H_{18}NO_2S$ (M+NH_4^+) 288.1053].

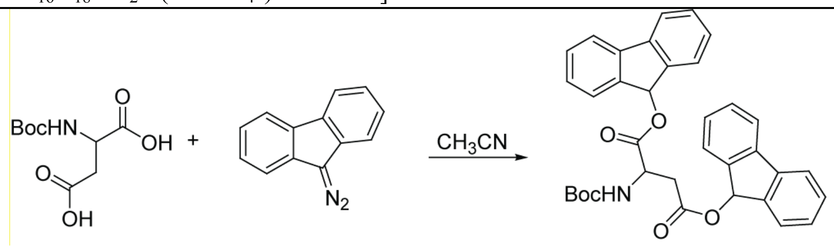

Diazofluorene (0.012 g, 0.063 mmol) was added to a solution of Boc-aspartic acid (0.007 g, 0.0315 mmol) in anhydrous acetonitrile (0.31 mL), and the reaction mixture was stirred for 5 h before being concentrated under reduced pressure and purified by silica gel chromatography to give bisfluorenyl-Boc-aspartate (0.019 g, 89%).

^{1}H NMR (500 MHz, CDCl$_3$) *δ* 7.74–7.64 (m, 4H), 7.59–7.51 (m, 4H), 7.50–7.37 (m, 4H), 7.36–7.15 (m, 4H), 6.90 (s, 1H), 6.77 (s, 1H), 5.70 (d, *J* = 8.6 Hz, 1H), 4.83–4.72 (m, 1H), 3.14 (dd, *J* = 17.1, 4.5 Hz, 1H), 3.00 (dd, *J* = 17.1, 4.7 Hz, 1H), 1.49 (s, 9H). **^{13}C NMR (126 MHz, CDCl$_3$)** *δ* 172.0, 172.0, 155.7, 141.6, 141.5, 141.3, 141.2, 129.9, 129.8, 128.2, 128.1, 126.4, 126.1, 120.3, 120.2, 80.5, 76.4, 75.9, 50.6, 37.2, 28.5. **HRMS** (ESI) *m*/*z* 579.2478 [calc'd for $C_{35}H_{35}N_2O_6$ (M+NH_4^+) 579.2490].

B. Esterification Screening in Acetonitrile/Buffer Solution

Representative Procedure: Each mixture was reacted for 6 h and was analyzed at that time.
Diazofluorene (0.0060 g, 0.0313 mmol) was added to a solution of bromoacetic acid (0.0044 g, 0.0313 mmol) in a mixture of acetonitrile:buffer (10 mM MES–NaOH, pH 5.5) (0.4 mL), and the reaction mixture was stirred for 6 h. The reaction mixture was concentrated under reduced pressure, and the ratio of products was determined by ^{1}H-NMR. The ester data was reported above for each compound and below are the data for the hydrolysis products used for comparison.

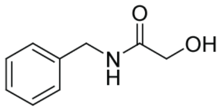

***N*-Benzylacetamidyl Hydrolysis Product:**
^{1}H NMR (400 MHz, CDCl$_3$) *δ* 7.38–7.28 (m, 5H), 4.52 (d, *J* = 5.9 Hz, 2H), 4.19 (d, *J* = 5.2 Hz, 2H), 2.24 (t, *J* = 5.2 Hz, 1H). **^{13}C NMR (126 MHz, CDCl$_3$)** δ 171.2, 138.0, 129.0, 128.1, 127.9, 62.5, 43.3. **HRMS** (ESI) *m*/*z* 166.0864 [calc'd for $C_9H_{12}NO_2$ (M+H^+) 166.0863].

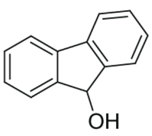

Fluorenyl Hydrolysis Product:
^{1}H NMR (400 MHz, CDCl$_3$) δ 7.66 (d, *J* = 7.6 Hz, 4H), 7.40 (t, *J* = 7.6 Hz, 2H), 7.33 (t, *J* = 7.6 Hz, 2H), 5.60 (bs, 1H). **^{13}C NMR (126 MHz, CDCl$_3$)** δ 145.8, 140.2, 129.3, 128.1, 125.4, 120.2, 75.5. **HRMS** (EI) *m*/*z* 182.0724 [calc'd for $C_{13}H_{10}O$ (M^+) 182.0727].

C. Thioester versus Thionoester Formation with Thioacetic Acid

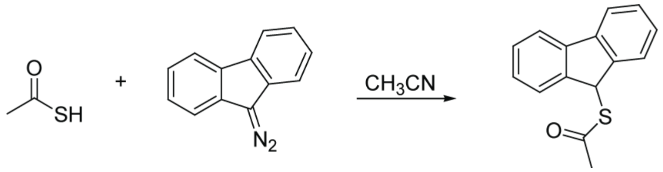

Diazofluorene (0.017 g, 0.089 mmol) was added to a solution of thioacetic acid (0.007 g, 0.089 mmol) in anhydrous acetonitrile (0.9 mL), and the reaction mixture was stirred for 1 min, when the reaction was determined to be complete by thin-layer chromatography (R_f = 0.8 in 30% EtOAc, 70% hexanes). The reaction mixture was concentrated under reduced pressure and purified by silica gel chromatography to give fluorenyl-thioacetate (0.020 g, 94%) in which sulfur was incorporated exclusively.

^{1}H NMR (400 MHz, $CDCl_3$) δ 7.73 (d, J = 7.5 Hz, 2H), 7.54 (d, J = 7.5 Hz, 2H), 7.40 (t, J = 7.5 Hz, 2H), 7.32 (t, J = 7.5 Hz, 2H), 5.88 (s, 1H), 2.52 (s, 3H). **^{13}C NMR (126 MHz, $CDCl_3$)** δ 196.3, 144.0, 140.9, 128.5, 127.8, 125.6, 120.2, 46.9, 30.7. **HRMS** (ESI) m/z 258.0953 [calc'd for $C_{15}H_{16}NOS$ ($M+NH_4^+$) 258.0948].

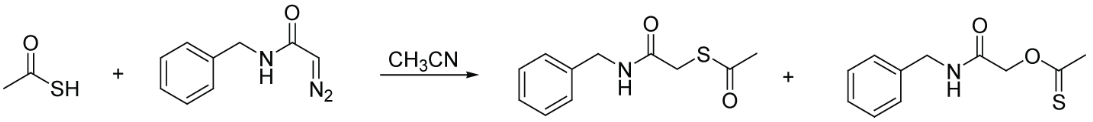

Diazobenzylacetamide (0.010 g, 0.057 mmol) was added to a solution of thioacetic acid (0.004 mL, 0.057 mmol) in anhydrous acetonitrile (0.57 mL), and the reaction mixture was stirred for 1 h, when the reaction was determined to be complete by thin-layer chromatography (R_f = 0.6, 0.7 in 70% EtOAc, 30% hexanes). The reaction mixture was concentrated under reduced pressure and purified by silica gel chromatography to give both benzyl acetamide-thioacetate (0.008 g, 62%) and benzyl-acetamido-thionoacetate (0.004 g, 31%).

Sulfur Attack (Benzyl-acetamide-thioacetate [R_f = 0.6]):

^{1}H NMR (400 MHz, $CDCl_3$) δ 7.40–7.17 (m, 5H), 6.47 (bs, 1H), 4.43 (d, J = 5.8 Hz, 2H), 3.59 (s, 2H), 2.39 (s, 3H). **^{13}C NMR (126 MHz, $CDCl_3$)** δ 195.9, 168.0, 137.8, 128.8, 127.6, 127.6, 43.8, 33.0, 30.3. **HRMS** (ESI) m/z 224.0746 [calc'd for $C_{11}H_{14}NO_2S$ (M+H+) 224.0740].

Oxygen Attack (Benzyl-acetamide-thionoacetate [R_f = 0.7]:

^{1}H NMR (400 MHz, $CDCl_3$) δ 7.44–7.25 (m, 5H), 6.39 (bs, 1H), 4.98 (s, 2H), 4.54 (d, J = 5.9 Hz, 2H), 2.64 (s, 3H). **^{13}C NMR (126 MHz, $CDCl_3$)** δ 217.5, 166.1, 137.5, 128.9, 127.9, 127.8, 69.8, 43.2, 34.2. **HRMS** (ESI) m/z 246.0552 [calc'd for $C_{11}H_{13}NO_2SNa$ ($M+Na^+$) 246.0560].

---

3. RNase A-Esterification Experiments

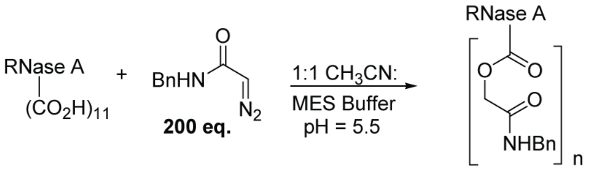

Ribonuclease A (0.001 g, 0.073 µmol) was dissolved in 100 µL of 10 mM MES–NaOH buffer, pH 5.5, and diazobenzylacetamide (**1**) (0.0026 g, 14.6 µmol) was dissolved in 100 µL of acetonitrile. The two solutions were combined, and the reaction mixture was stirred for 4 h at 37 °C. Any remaining diazo compound was then quenched by adding 100 µL of 100 mM acetic acid, and the reaction mixture was concentrated under reduced pressure. The extent of esterification was determined to be <1 esters per RNase A by MALDI–TOF mass spectrometry. (Note: When the same conditions were employed with only 10 equiv of diazo compound, no esterification was observed).

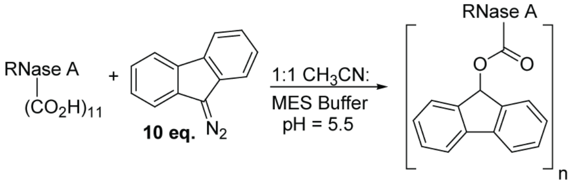

Ribonuclease A (0.001 g, 0.073 μmol) was dissolved in 100 μL of 10 mM MES–NaOH buffer, pH 5.5, and diazofluorene (**2**) (0.001 g, 5.2 μmol) was dissolved in 1.00 mL of acetonitrile. An aliquot (100 μL) of the latter solution was added to the former solution, and the reaction mixture was stirred for 4 h at 37 °C. Any remaining diazo compound was then quenched by adding 100 μL of 100 mM acetic acid, and the reaction mixture was concentrated under reduced pressure. The extent of esterification was determined to be ~3 esters per RNase A by MALDI–TOF mass spectrometry.

4. Trypsin-Digestion and MS/MS Experiments to Identify Esterified Carboxyl Groups in RNase A

Protein digestion and mass spectrometric analysis was performed at the Mass Spectrometry Facility of the Biotechnology Center at the University of Wisconsin–Madison. Briefly, crude RNase A (0.10 mg) was precipitated with three volumes of ice-cold acetone, incubated for 30 min at –20 °C, and subjected to centrifugation for 10 min at 16,000*g*. The pellet was washed once with ice-cold acetone, subjected to centrifugation again, and then washed once more with ice-cold methanol. The pelleted protein was re-solubilized and denatured in 15 μL of 8 M urea/50 mM NH_4HCO_3 (pH 8.5) for 10 min, then diluted with 60 μL containing 2.5 μL of 25 mM dithiothreitol (DTT), 5 μL MeOH, 0.2 μL of 1.0 M Tris–HCl buffer, pH 7.5, and 37.3 μL of 25 mM NH_4HCO_3 (pH 8.5). The resulting solution was incubated at 50 °C for 15 min, and then cooled with ice to room temperature. A 3-μL aliquot of 55 mM iodoacetamide was added, and the resulting solution was incubated in darkness at room temperature for 15 min before the reaction was quenched by adding 8 μL of 25 mM DTT. Subsequently were added 14 μL of 25 mM NH_4HCO_3, pH 8.5, and 15 μL of trypsin solution, which was 100 ng/μl Trypsin Gold from Promega (Madison, WI) in 25 mM NH_4HCO_3, to achieve a final volume of 100 μL. Digestion with trypsin was allowed to proceed for 1 h at 42 °C. Then, an additional 10 μL of trypsin solution was added such that the final enzyme/substrate w/w ratio was 1:40, and digestion was allowed to proceed overnight at 37 °C. Digestion was terminated by acidification with 2.5% w/v trifluoroacetic acid to a final concentration of 0.3% w/v, and 8 μL (~6.5 μg RNase A) was used for nanoLC-MS/MS analysis.

HPLC was performed using a 1100 series system from Agilent Technologies (Santa Clara, CA) equipped with an isocratic loading pump and nano-flow gradient pump at a flow rate of 15 μL/min. Samples of RNase A (~6.5 μg) were washed from the autosampler onto a 0.3 × 5 mm Stablebond C18 trapping cartridge. For elution, the nano-flow pump was switched into line with the trapping cartridge, and peptides were eluted onto an in-house–fabricated 15-cm resolving C18 column from Bruker–Michrom (Auburn, CA) with a laser-pulled tip (P-2000) from Sutter Instrument (Novato, CA). Peptides were eluted with solvents comprised of 0.1 M acetic acid in water (solvent A) and 0.1 M acetic acid/95% v/v acetonitrile in water (solvent B). The gradient consisted of a 20-min loading and desalting period with column equilibration at 1% solvent B, an increase to 40% B over 195 min, a ramp to 60% B over 20 min, an increase to 100% B in 5 min, and a hold for 3 min. The column was then re-equilibrated at 1% B for 30 min. The flow rate for peptide elution and re-equilibration was 200 nL/min.

Peptides were analyzed by nanoLC-MS/MS using the 1100 series system connected to an LTQ-Orbitrap XL hybrid linear ion trap-orbitrap mass spectrometer from Thermo Fisher Scientific (Waltham, MA) equipped with a nanoelectrospray ion source. Capillary HPLC was performed using an in-house–fabricated 15-cm C18 column packed with Jupiter 4-μm C12 particles from Phenomenex (Torrance, CA) and a laser-pulled tip (P-2000) from Sutter Instrument with 360 × 75 μm fused silica tubing. Sample loading (8 μL) and desalting were done at 10 μL/min using a trapping column in line with the autosampler (Zorbax 300SB-C18, 5 μM, 5 × 0.3 mm from Agilent Technologies). Peptide elution used solvents comprised of 0.1% v/v formic acid in water (solvent A) and 0.1% v/v formic acid/95% v/v acetonitrile in water (solvent B). The gradient consisted of a 20-min loading and desalting period with column equilibration at 1% solvent B, an increase to 40% B over 195 min, ramp to 60% B over 20 min, an increase to 100% B in 5 min, and a hold for 3 min. The column was then re-equilibrated at 1% B for 30 min. The flow rate for peptide elution and re-equilibration was 200 nL/min.

The LTQ-Orbitrap was set to acquire MS/MS spectra in data-dependent mode as follows. MS survey scans from *m*/*z* 300 to 2000 were collected in centroid mode at a resolving power of 100,000. MS/MS spectra were collected on the five most-abundant signals in each survey scan. Dynamic exclusion was employed to increase dynamic range and maximize peptide identifications. This feature excluded precursors up to 0.55 *m*/*z* below and 1.05 *m*/*z* above previously selected precursors. Precursors remained on the exclusion list for 40 s. Peptide mass tolerance was set at 20 ppm and fragment mass at 0.8 Da. Singly-charged ions and ions for which the charge state could not be assigned were rejected from consideration.

5. FLAG–RNase A-Esterification Experiments

Preparation of FLAG–RNase A

The FLAG sequence (DYKDDDDK) was inserted between the N-terminal methionine and Lys2 of RNase A in the pET22b(+) vector that directs the expression of wild-type RNase A in *Escherichia coli*. The protein was produced and purified by methods similar to those described previously.[1] The protein was characterized by SDS–PAGE and MALDI–TOF (*m*/*z* 14816) (Figure S11A). The purified protein was obtained at approximately 25 mg/L.

Esterification of FLAG–RNase A

The esterification of FLAG–RNase A with diazofluorene (**2**) was carried out as described in Section 3. Esterification was verified by MALDI–TOF, and determined to be of similar magnitude to that of native RNase A (Figure S11B and S11C).

Hydrolysis of Esterified Carboxyl Groups in FLAG–RNase A by a HeLa Cell Lysate

HeLa cells were grown to confluence in a 10-cm^2 dish before their collection and lysis with M-PER mammalian protein extraction reagent from Thermo Fisher Scientific. The presence of esterase activity in the lysate was verified by a colorimetric assay using *p*-nitrophenyl acetate. A solution of esterified FLAG–RNase A (10 μg) was added to 200 μL of HeLa cell lysate, and the reaction mixture was nutated at ambient temperature overnight. FLAG–RNase A was subsequently purified with Anti-FLAG® M2 Magnetic Beads (Sigma–Aldrich). The regeneration of native FLAG–RNase A was confirmed with MALDI–TOF mass spectrometry (Figure S12) and assays of ribonucleolytic activity (Figure S13).

Ribonucleolytic Activity Assays

The ribonucleolytic activity of RNase A, FLAG–RNase A, FLAG–RNase A esterified with diazofluorene (**2**) (10 or 200 equiv), and esterified FLAG–RNase A exposed to HeLa cell lysate was determined by quantifying the cleavage of 6-FAM–dArUdAdA–6-TAMRA, as described previously.[2] Assays were carried out in triplicate at ambient temperature in a black polystyrene 96-well plate in 200 μL of 0.10 M MES–NaOH buffer, pH 6.0, containing NaCl (0.10 M). The resulting fluorescence data were fitted to the equation: $k_{cat}/K_M = (\Delta I/\Delta t)/(I_f - I_0)[E]$, in which $\Delta I/\Delta t$ is the initial reaction velocity, I_0 is the fluorescence intensity before addition of any ribonuclease, I_f is the fluorescence intensity after complete substrate hydrolysis, and [E] is the total ribonuclease concentration.

6. His$_6$–RFP-Esterification Experiments

Preparation of His$_6$–RFP

A gene encoding an mCherry variant of the red fluorescent protein (RFP)[3] was inserted into a novel vector derived from the pET22b from Novagen (Madison, WI). The resulting vector encoded an N-terminal His$_6$ tag followed by a spacer region and a TEV protease recognition sequence. The vector also contained a T7 promoter and ampicillin-resistance gene, but not *lacI*. (As RFP is not toxic to *E. coli*, its leaky gene expression is not a concern.) The vector was modified to contain a *Stu*I site immediately after the TEV cleavage sequence, allowing for facile insertion of target genes with an N-terminal tag.

The expresson vector was transformed into electrocompetent BL21(DE3) *E. coli* cells from New England Biolabs (Ipswich MA), which were then plated on LB–agar containing ampicillin. On the following day, a single colony was used to inoculate 50 mL of LB medium, and the resulting culture was grown overnight at 37 °C in a shaking incubator. On the following day, 5 mL of starter culture was used to inoculate 1 L of Terrific Broth medium (Research Products International) prepared previously in a 3.8-L glass flask. Ampicillin was also added to each flask to a final concentration of 200 μg/mL. Flasks were shaken at 200 rpm at 37 °C in a large shaking incubator until cells reached log phase (*OD* 0.6–0.8 at 600 nm). The incubator temperature was then switched to 20 °C, and cells

were equilibrated at the new temperature for 20 min. The production of His_6–RFP was induced by adding IPTG from Research Products International (Mt. Prospect, IL) to 1 mM, and the cells were grown overnight at 20 °C in a shaking incubator.

Cells were harvested by centrifugation for 20 min at 5,000 rpm at 4 °C. Cell pellets, which were bright magenta in color, were collected and resuspended in a lysis buffer, which was 50 mM Tris–HCl buffer, pH 7.0, containing NaCl (100 mM), imidazole (30 mM), Triton X-100 (1% v/v), and sucrose (20% w/v). The buffer was filter-sterilized, but not autoclaved, and 15 mL of buffer was used for every 2 L of liquid growth. Cell pellets were vortexed and stored frozen at –20 °C overnight.

Cells were lysed with a TS Series cell disrupter from Constant Systems (Kennesaw, GA), and the lysate was subjected to centrifugation immediately for 1 h at 11,000 rpm at 4 °C. The supernatant was filtered with either 5-μM syringe filters from EMD Millipore (Darmstadt, Germany) or glass fiber pre-filters from Sartorius AG (Göttingen, Germany). Solid, pelleted material was discarded. Filtered supernatants were stored on ice and protected from light prior to protein purification.

Filtered cell lysates were purified by chromatography on Ni–NTA resin from GE Healthcare (Little Chalfont, UK) or Thermo Fisher Scientific, and elution using a linear gradient of imidazole. The binding (wash) buffer was 20 mM sodium phosphate buffer, pH 7.4, containing NaCl (500 mM) and imidazole (30 mM). The elution buffer was 20 mM sodium phosphate buffer, pH 7.4, containing NaCl (500 mM) and imidazole (500 mM). Eluted fractions were collected, pooled, and dialyzed against 4 L of 20 mM Tris–HCl buffer, pH 7.0, containing EDTA (1 mM). Dialyzed material was then purified again using anion-exchange chromatography on a hiTrap Q column. Protein was eluted by using a linear gradient of NaCl. The binding (wash) buffer was 20 mM Tris–HCl buffer, pH 7.0, containing EDTA (1 mM). The elution buffer was 20 mM Tris–HCl buffer, pH. 7.0, containing EDTA (1 mM), NaCl (1.0 M). Upon elution, colored fractions were pooled and concentrated if necessary. The yield of His_6–RFP was 55 mg per L of culture.

Esterification of His_6–RFP

RFP (0.015 g, 0.51 μmol) was dissolved in 1.70 mL of 10 mM MES–NaOH buffer, pH 5.5, and diazofluorene (**2**) (0.001 g, 5.21 μmol) was dissolved in 1.70 mL of acetonitrile. The two solutions were combined, and the reaction mixture was nutated for 12 h in the dark. Any remaining diazo compound was then quenched by adding 100 μL of 100 mM acetic acid. The extent of esterification was determined to be 1–3 esters per RFP by MALDI–TOF mass spectrometry.

Hydrolysis of Esterified His_6–RFP by a HeLa Cell Lysate

HeLa cells were grown to confluence in a 10-cm^2 dish before their collection and lysis with M-PER mammalian protein extraction reagent from Thermo Fisher Scientific. The presence of esterase activity in the lysate was verified by a colorimetric assay using *p*-nitrophenyl acetate. A solution of esterified His_6–RFP (10 μg) was added to 200 μL of HeLa cell lysate, and the reaction mixture was nutated at ambient temperature overnight. His_6–RFP was subsequently purified with HisPur Ni–NTA magnetic beads from Thermo Fisher Scientific. The regeneration of native His_6–RFP was confirmed with MALDI–TOF mass spectrometry (Figure S14).

7. MALDI–TOF Mass Spectrometry Data for RNase A-Esterification Experiments

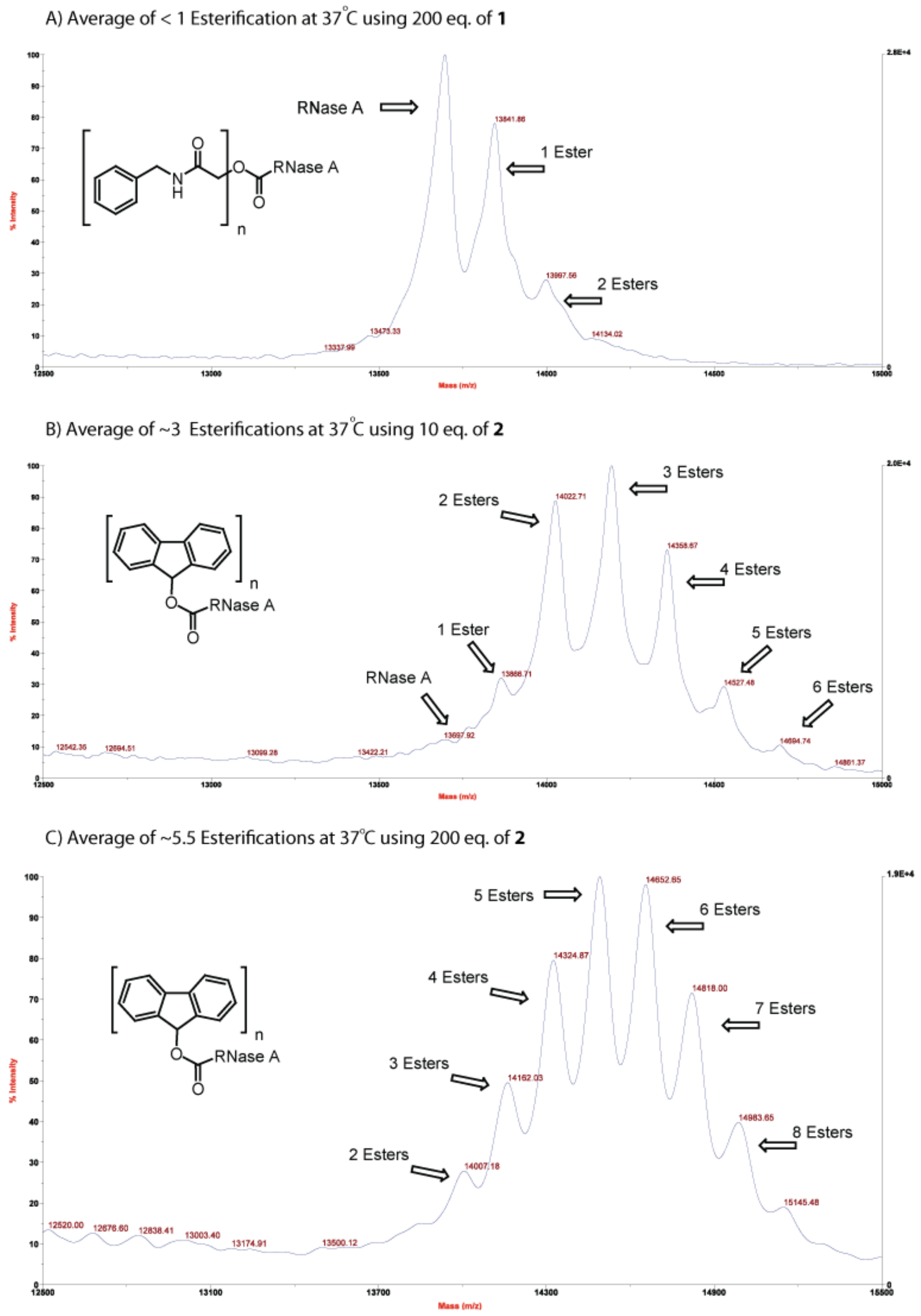

Figure S1. MALDI MS of Labeled RNase A

8. MS/MS Data for Trypsin-Digestion Experiments

MATRIX SCIENCE Mascot Search Results

Peptide View

MS/MS Fragmentation of FERQHMDSSTSAASSSNYCNQMMK
Found in gi|111141, RNase A 27-150

Match to Query 3128: 2943.197704 from(736.806702,4+) intensity(27277.8000)
Title: NAM_100-20.01662.01662.4
Data file NAM_100-20.mgf

Click mouse within plot area to zoom in by factor of two about that point
Or, Plot from 200 to 1700 Da Full range

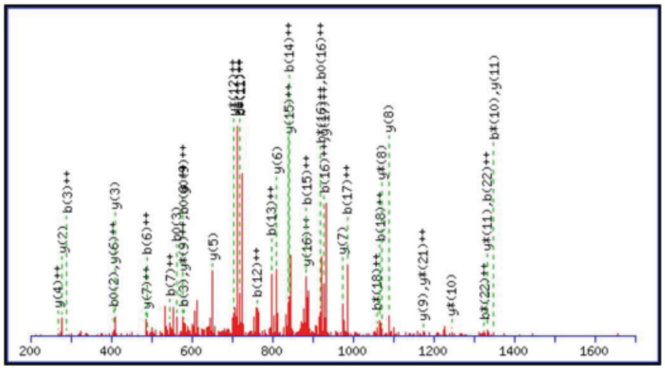

Monoisotopic mass of neutral peptide Mr(calc): 2943.2044
Fixed modifications: Carbamidomethyl (C)
Variable modifications:
E2 : Raines_147 (DE)
Ions Score: 28 Expect: 0.00022
Matches (Bold Red): 43/254 fragment ions using 103 most intense peaks

#	b	b^{++}	b^*	b^{*++}	b^0	b^{0++}	Seq.	y	y^{++}	y^*	y^{*++}	y^0	y^{0++}	#
1	148.0757	74.5415					F							24
2	424.1867	212.5970			406.1761	203.5917	E	2797.1433	1399.0753	2780.1168	1390.5620	2779.1327	1390.0700	23
3	580.2878	290.6475	563.2613	282.1343	562.2772	281.6423	R	2521.0323	1261.0198	2504.0057	1252.5065	2503.0217	1252.0145	22
4	708.3464	354.6768	691.3198	346.1636	690.3358	345.6716	Q	2364.9312	1182.9692	2347.9046	1174.4560	2346.9206	1173.9639	21
5	845.4053	423.2063	828.3788	414.6930	827.3947	414.2010	H	2236.8726	1118.9399	2219.8460	1110.4267	2218.8620	1109.9347	20
6	976.4458	488.7265	959.4192	480.2133	958.4352	479.7213	M	2099.8137	1050.4105	2082.7871	1041.8972	2081.8031	1041.4052	19
7	1091.4727	546.2400	1074.4462	537.7267	1073.4622	537.2347	D	1968.7732	984.8902	1951.7467	976.3770	1950.7626	975.8850	18
8	1178.5048	589.7560	1161.4782	581.2427	1160.4942	580.7507	S	1853.7463	927.3768	1836.7197	918.8635	1835.7357	918.3715	17
9	1265.5368	633.2720	1248.5102	624.7588	1247.5262	624.2667	S	1766.7142	883.8608	1749.6877	875.3475	1748.7037	874.8555	16
10	1366.5845	683.7959	1349.5579	675.2826	1348.5739	674.7906	T	1679.6822	840.3447	1662.6557	831.8315	1661.6716	831.3395	15
11	1453.6165	727.3119	1436.5899	718.7986	1435.6059	718.3066	S	1578.6345	789.8209	1561.6080	781.3076	1560.6240	780.8156	14
12	1524.6536	762.8304	1507.6271	754.3172	1506.6430	753.8252	A	1491.6025	746.3049	1474.5759	737.7916	1473.5919	737.2996	13
13	1595.6907	798.3490	1578.6642	789.8357	1577.6802	789.3437	A	1420.5654	710.7863	1403.5388	702.2731	1402.5548	701.7810	12
14	1682.7228	841.8650	1665.6962	833.3517	1664.7122	832.8597	S	1349.5283	675.2678	1332.5017	666.7545	1331.5177	666.2625	11
15	1769.7548	885.3810	1752.7282	876.8678	1751.7442	876.3757	S	1262.4962	631.7518	1245.4697	623.2385	1244.4857	622.7465	10
16	1856.7868	928.8970	1839.7603	920.3838	1838.7762	919.8918	S	1175.4642	588.2357	1158.4377	579.7225	1157.4536	579.2305	9
17	1970.8297	985.9185	1953.8032	977.4052	1952.8192	976.9132	N	1088.4322	544.7197	1071.4056	536.2065			8
18	2133.8931	1067.4502	2116.8665	1058.9369	2115.8825	1058.4449	Y	974.3893	487.6983	957.3627	479.1850			7
19	2293.9237	1147.4655	2276.8972	1138.9522	2275.9131	1138.4602	C	811.3259	406.1666	794.2994	397.6533			6
20	2407.9666	1204.4870	2390.9401	1195.9737	2389.9561	1195.4817	N	651.2953	326.1513	634.2687	317.6380			5
21	2536.0252	1268.5162	2518.9987	1260.0030	2518.0147	1259.5110	Q	537.2524	269.1298	520.2258	260.6165			4
22	2667.0657	1334.0365	2650.0392	1325.5232	2649.0551	1325.0312	M	409.1938	205.1005	392.1672	196.5873			3
23	2798.1062	1399.5567	2781.0796	1391.0435	2780.0956	1390.5514	M	278.1533	139.5803	261.1267	131.0670			2
24							K	147.1128	74.0600	130.0863	65.5468			1

Figure S2. Benzylacetamidyl ester of glutamic acid 9 of RNase A.

Mascot Search Results

Peptide View

MS/MS Fragmentation of QHMDSSTSAASSSNYCNQMMK
Found in gi|111141, RNase A 27-150

Match to Query 2547: 2510.978172 from(838.000000,3+) intensity(109235.0000)
Title: NAM_100-20.01711.01711.3
Data file NAM_100-20.mgf

Click mouse within plot area to zoom in by factor of two about that point
Or, Plot from 200 to 1800 Da Full range

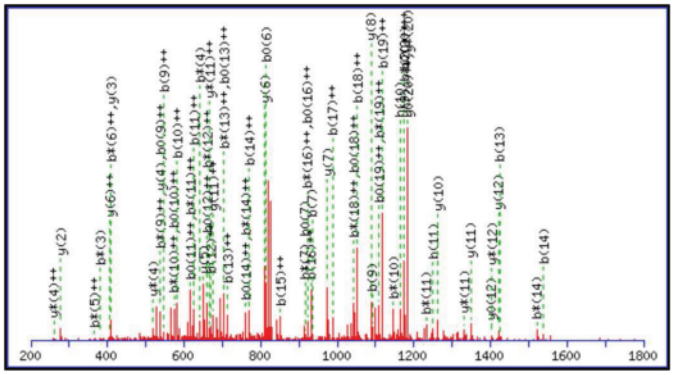

Monoisotopic mass of neutral peptide Mr(calc): 2510.9923
Fixed modifications: Carbamidomethyl (C)
Variable modifications:
D4 : Raines_147 (DE)
Ions Score: 49 Expect: 5.7e-007
Matches (Bold Red): 69/218 fragment ions using 120 most intense peaks

#	b	b^{++}	b^{*}	b^{*++}	b^{0}	b^{0++}	Seq.	y	y^{++}	y^{*}	y^{*++}	y^{0}	y^{0++}	#
1	129.0659	65.0366	112.0393	56.5233			Q							21
2	266.1248	133.5660	249.0982	125.0527			H	2383.9410	1192.4741	2366.9145	1183.9609	2365.9305	1183.4689	20
3	397.1653	199.0863	380.1387	190.5730			M	2246.8821	1123.9447	2229.8556	1115.4314	2228.8715	1114.9394	19
4	659.2606	330.1339	642.2341	321.6207	641.2500	321.1287	D	2115.8416	1058.4244	2098.8151	1049.9112	2097.8311	1049.4192	18
5	746.2926	373.6500	729.2661	365.1367	728.2821	364.6447	S	1853.7463	927.3768	1836.7197	918.8635	1835.7357	918.3715	17
6	833.3247	417.1660	816.2981	408.6527	815.3141	408.1607	S	1766.7142	883.8608	1749.6877	875.3475	1748.7037	874.8555	16
7	934.3723	467.6898	917.3458	459.1765	916.3618	458.6845	T	1679.6822	840.3447	1662.6557	831.8315	1661.6716	831.3395	15
8	1021.4044	511.2058	1004.3778	502.6926	1003.3938	502.2005	S	1578.6345	789.8209	1561.6080	781.3076	1560.6240	780.8156	14
9	1092.4415	546.7244	1075.4149	538.2111	1074.4309	537.7191	A	1491.6025	746.3049	1474.5759	737.7916	1473.5919	737.2996	13
10	1163.4786	582.2429	1146.4521	573.7297	1145.4680	573.2377	A	1420.5654	710.7863	1403.5388	702.2731	1402.5548	701.7810	12
11	1250.5106	625.7590	1233.4841	617.2457	1232.5001	616.7537	S	1349.5283	675.2678	1332.5017	666.7545	1331.5177	666.2625	11
12	1337.5427	669.2750	1320.5161	660.7617	1319.5321	660.2697	S	1262.4962	631.7518	1245.4697	623.2385	1244.4857	622.7465	10
13	1424.5747	712.7910	1407.5481	704.2777	1406.5641	703.7857	S	1175.4642	588.2357	1158.4377	579.7225	1157.4536	579.2305	9
14	1538.6176	769.8124	1521.5911	761.2992	1520.6071	760.8072	N	1088.4322	544.7197	1071.4056	536.2065			8
15	1701.6809	851.3441	1684.6544	842.8308	1683.6704	842.3388	Y	974.3893	487.6983	957.3627	479.1850			7
16	1861.7116	931.3594	1844.6850	922.8462	1843.7010	922.3542	C	811.3259	406.1666	794.2994	397.6533			6
17	1975.7545	988.3809	1958.7280	979.8676	1957.7440	979.3756	N	651.2953	326.1513	634.2687	317.6380			5
18	2103.8131	1052.4102	2086.7865	1043.8969	2085.8025	1043.4049	Q	537.2524	269.1298	520.2258	260.6165			4
19	2234.8536	1117.9304	2217.8270	1109.4172	2216.8430	1108.9251	M	409.1938	205.1005	392.1672	196.5873			3
20	2365.8941	1183.4507	2348.8675	1174.9374	2347.8835	1174.4454	M	278.1533	139.5803	261.1267	131.0670			2
21							K	147.1128	74.0600	130.0863	65.5468			1

Figure S3. Benzylacetamidyl ester of aspartic acid 14 of RNase A.

Mascot Search Results

Peptide View

MS/MS Fragmentation of CKPVNTFVHESLADVQAVCSQK
Found in gi|111141, RNase A 27-150

Match to Query 2846: 2663.284386 from(888.768738,3+) intensity(452129.0000)
Title: NAM_100-20.02562.02562.3
Data file NAM_100-20.mgf

Click mouse within plot area to zoom in by factor of two about that point
Or, Plot from 200 to 2000 Da Full range

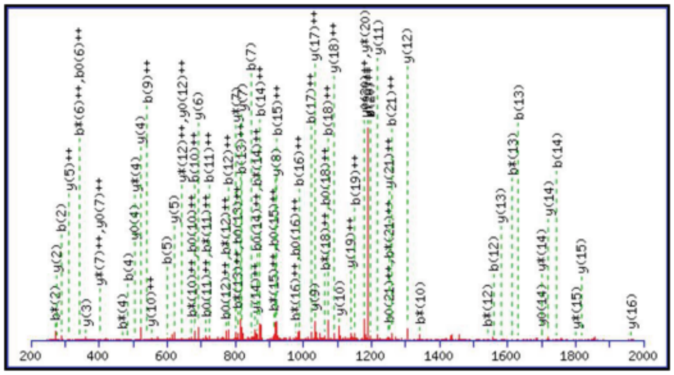

Monoisotopic mass of neutral peptide Mr(calc): 2663.2836
Fixed modifications: Carbamidomethyl (C)
Variable modifications:
E10 : Raines_147 (DE)
Ions Score: 62 Expect: 1.4e-006
Matches (Bold Red): 80/236 fragment ions using 179 most intense peaks

#	b	b^{++}	b*	b^{*++}	b^0	b^{0++}	Seq.	y	y^{++}	y*	y^{*++}	y^0	y^{0++}	#
1	161.0379	81.0226					C							22
2	289.1329	145.0701	272.1063	136.5568			K	2504.2602	1252.6338	2487.2337	1244.1205	2486.2497	1243.6285	21
3	386.1857	193.5965	369.1591	185.0832			P	2376.1653	1188.5863	2359.1387	1180.0730	2358.1547	1179.5810	20
4	485.2541	243.1307	468.2275	234.6174			V	2279.1125	1140.0599	2262.0860	1131.5466	2261.1019	1131.0546	19
5	599.2970	300.1521	582.2704	291.6389			N	2180.0441	1090.5257	2163.0175	1082.0124	2162.0335	1081.5204	18
6	700.3447	350.6760	683.3181	342.1627	682.3341	341.6707	T	2066.0012	1033.5042	2048.9746	1024.9909	2047.9906	1024.4989	17
7	847.4131	424.2102	830.3865	415.6969	829.4025	415.2049	F	1964.9535	982.9804	1947.9269	974.4671	1946.9429	973.9751	16
8	946.4815	473.7444	929.4550	465.2311	928.4709	464.7391	V	1817.8851	909.4462	1800.8585	900.9329	1799.8745	900.4409	15
9	1083.5404	542.2738	1066.5139	533.7606	1065.5298	533.2686	H	1718.8167	859.9120	1701.7901	851.3987	1700.8061	850.9067	14
10	1359.6514	680.3294	1342.6249	671.8161	1341.6409	671.3241	E	1581.7577	791.3825	1564.7312	782.8692	1563.7472	782.3772	13
11	1446.6835	723.8454	1429.6569	715.3321	1428.6729	714.8401	S	1305.6467	653.3270	1288.6202	644.8137	1287.6362	644.3217	12
12	1559.7675	780.3874	1542.7410	771.8741	1541.7570	771.3821	L	1218.6147	609.8110	1201.5882	601.2977	1200.6041	600.8057	11
13	1630.8046	815.9060	1613.7781	807.3927	1612.7941	806.9007	A	1105.5306	553.2690	1088.5041	544.7557	1087.5201	544.2637	10
14	1745.8316	873.4194	1728.8050	864.9062	1727.8210	864.4141	D	1034.4935	517.7504	1017.4670	509.2371	1016.4830	508.7451	9
15	1844.9000	922.9536	1827.8734	914.4404	1826.8894	913.9483	V	919.4666	460.2369	902.4400	451.7237	901.4560	451.2316	8
16	1972.9586	986.9829	1955.9320	978.4696	1954.9480	977.9776	Q	820.3982	410.7027	803.3716	402.1894	802.3876	401.6974	7
17	2043.9957	1022.5015	2026.9691	1013.9882	2025.9851	1013.4962	A	692.3396	346.6734	675.3130	338.1602	674.3290	337.6681	6
18	2143.0641	1072.0357	2126.0375	1063.5224	2125.0535	1063.0304	V	621.3025	311.1549	604.2759	302.6416	603.2919	302.1496	5
19	2303.0947	1152.0510	2286.0682	1143.5377	2285.0842	1143.0457	C	522.2341	261.6207	505.2075	253.1074	504.2235	252.6154	4
20	2390.1268	1195.5670	2373.1002	1187.0537	2372.1162	1186.5617	S	362.2034	181.6053	345.1769	173.0921	344.1928	172.6001	3
21	2518.1853	1259.5963	2501.1588	1251.0830	2500.1748	1250.5910	Q	275.1714	138.0893	258.1448	129.5761			2
22							K	147.1128	74.0600	130.0863	65.5468			1

Figure S4. Benzylacetamidyl ester of glutamic acid 49 of RNase A.

Mascot Search Results

Peptide View

MS/MS Fragmentation of HIIVACEGNPYVPVHFDASV
Found in gi|111141, RNase A 27-150

Match to Query 2249: 2370.148278 from(791.056702,3+) intensity(68902.3000)
Title: NAM_100-20.02716.02716.3
Data file NAM_100-20.mgf

Click mouse within plot area to zoom in by factor of two about that point
Or, Plot from 200 to 1900 Da Full range

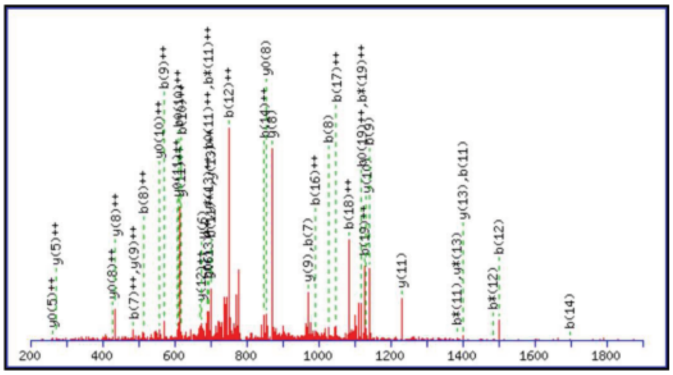

Monoisotopic mass of neutral peptide Mr(calc): 2370.1467
Fixed modifications: Carbamidomethyl (C)
Variable modifications:
E7 : Raines_147 (DE)
Ions Score: 27 Expect: 0.0045
Matches (Bold Red): 45/176 fragment ions using 134 most intense peaks

#	b	b^{++}	b*	b^{*++}	b^0	b^{0++}	Seq.	y	y^{++}	y*	y^{*++}	y^0	y^{0++}	#
1	138.0662	69.5367					H							20
2	251.1503	126.0788					I	2234.0951	1117.5512	2217.0685	1109.0379	2216.0845	1108.5459	19
3	364.2343	182.6208					I	2121.0110	1061.0091	2103.9844	1052.4959	2103.0004	1052.0039	18
4	463.3027	232.1550					V	2007.9269	1004.4671	1990.9004	995.9538	1989.9164	995.4618	17
5	534.3398	267.6736					A	1908.8585	954.9329	1891.8320	946.4196	1890.8480	945.9276	16
6	694.3705	347.6889					C	1837.8214	919.4143	1820.7949	910.9011	1819.8108	910.4091	15
7	970.4815	485.7444			952.4709	476.7391	E	1677.7908	839.3990	1660.7642	830.8857	1659.7802	830.3937	14
8	1027.5030	514.2551			1009.4924	505.2498	G	1401.6797	701.3435	1384.6532	692.8302	1383.6692	692.3382	13
9	1141.5459	571.2766	1124.5193	562.7633	1123.5353	562.2713	N	1344.6583	672.8328	1327.6317	664.3195	1326.6477	663.8275	12
10	1238.5987	619.8030	1221.5721	611.2897	1220.5881	610.7977	P	1230.6154	615.8113			1212.6048	606.8060	11
11	1401.6620	701.3346	1384.6354	692.8214	1383.6514	692.3294	Y	1133.5626	567.2849			1115.5520	558.2796	10
12	1500.7304	750.8688	1483.7039	742.3556	1482.7198	741.8636	V	970.4993	485.7533			952.4887	476.7480	9
13	1597.7832	799.3952	1580.7566	790.8819	1579.7726	790.3899	P	871.4308	436.2191			853.4203	427.2138	8
14	1696.8516	848.9294	1679.8250	840.4162	1678.8410	839.9241	V	774.3781	387.6927			756.3675	378.6874	7
15	1833.9105	917.4589	1816.8839	908.9456	1815.8999	908.4536	H	675.3097	338.1585			657.2991	329.1532	6
16	1980.9789	990.9931	1963.9524	982.4798	1962.9683	981.9878	F	538.2508	269.6290			520.2402	260.6237	5
17	2096.0059	1048.5066	2078.9793	1039.9933	2077.9953	1039.5013	D	391.1823	196.0948			373.1718	187.0895	4
18	2167.0430	1084.0251	2150.0164	1075.5118	2149.0324	1075.0198	A	276.1554	138.5813			258.1448	129.5761	3
19	2254.0750	1127.5411	2237.0484	1119.0279	2236.0644	1118.5359	S	205.1183	103.0628			187.1077	94.0575	2
20							V	118.0863	59.5468					1

Figure S5. Benzylacetamidyl ester of glutamic acid 111 of RNase A.

Mascot Search Results

Peptide View

MS/MS Fragmentation of HIIVACEGNPYVPVHFDASV
Found in gi|111141, RNase A 27-150

Match to Query 2252: 2370.152121 from(791.057983,3+) intensity(3214070.0000)
Title: NAM_100-20.02893.02893.3
Data file NAM_100-20.mgf

Click mouse within plot area to zoom in by factor of two about that point
Or, Plot from 200 to 1800 Da Full range

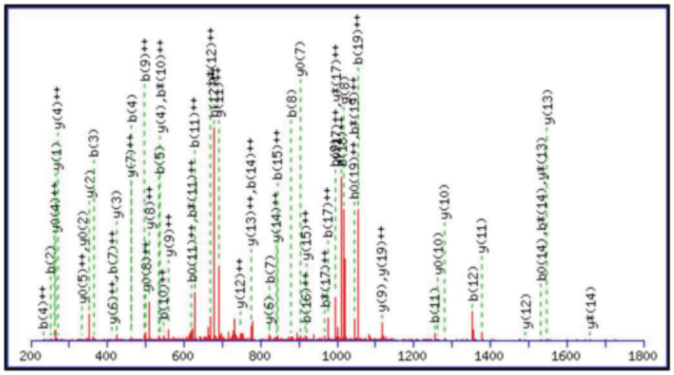

Monoisotopic mass of neutral peptide Mr(calc): 2370.1467
Fixed modifications: Carbamidomethyl (C)
Variable modifications:
C-term : Raines_147_term (Protein C-term)
Ions Score: 56 Expect: 5.9e-006
Matches (Bold Red): 62/176 fragment ions using 135 most intense peaks

#	b	b++	b*	b*++	b0	b0++	Seq.	y	y++	y*	y*++	y0	y0++	#
1	138.0662	69.5367					H							20
2	251.1503	126.0788					I	2234.0950	1117.5512	2217.0685	1109.0379	2216.0845	1108.5459	19
3	364.2343	182.6208					I	2121.0110	1061.0091	2103.9844	1052.4959	2103.0004	1052.0038	18
4	463.3027	232.1550					V	2007.9269	1004.4671	1990.9004	995.9538	1989.9164	995.4618	17
5	534.3398	267.6736					A	1908.8585	954.9329	1891.8320	946.4196	1890.8479	945.9276	16
6	694.3705	347.6889					C	1837.8214	919.4143	1820.7948	910.9011	1819.8108	910.4091	15
7	823.4131	412.2102			805.4025	403.2049	E	1677.7907	839.3990	1660.7642	830.8857	1659.7802	830.3937	14
8	880.4346	440.7209			862.4240	431.7156	G	1548.7481	774.8777	1531.7216	766.3644	1530.7376	765.8724	13
9	994.4775	497.7424	977.4509	489.2291	976.4669	488.7371	N	1491.7267	746.3670	1474.7001	737.8537	1473.7161	737.3617	12
10	1091.5302	546.2688	1074.5037	537.7555	1073.5197	537.2635	P	1377.6838	689.3455			1359.6732	680.3402	11
11	1254.5936	627.8004	1237.5670	619.2871	1236.5830	618.7951	Y	1280.6310	640.8191			1262.6204	631.8139	10
12	1353.6620	677.3346	1336.6354	668.8214	1335.6514	668.3293	V	1117.5677	559.2875			1099.5571	550.2822	9
13	1450.7147	725.8610	1433.6882	717.3477	1432.7042	716.8557	P	1018.4992	509.7533			1000.4887	500.7480	8
14	1549.7832	775.3952	1532.7566	766.8819	1531.7726	766.3899	V	921.4465	461.2269			903.4359	452.2216	7
15	1686.8421	843.9247	1669.8155	835.4114	1668.8315	834.9194	H	822.3781	411.6927			804.3675	402.6874	6
16	1833.9105	917.4589	1816.8839	908.9456	1815.8999	908.4536	F	685.3192	343.1632			667.3086	334.1579	5
17	1948.9374	974.9724	1931.9109	966.4591	1930.9269	965.9671	D	538.2507	269.6290			520.2402	260.6237	4
18	2019.9745	1010.4909	2002.9480	1001.9776	2001.9640	1001.4856	A	423.2238	212.1155			405.2132	203.1103	3
19	2107.0066	1054.0069	2089.9800	1045.4936	2088.9960	1045.0016	S	352.1867	176.5970			334.1761	167.5917	2
20							V	265.1547	133.0810					1

Figure S6. Benzylacetamidyl ester of valine 124 at the C terminus of RNase A.

Mascot Search Results

Peptide View

MS/MS Fragmentation of QHMDSSTSAASSSNYCNQMMK
Found in gi|111141, RNase A 27-150

Match to Query 3621: 2527.980063 from(843.667297,3+) intensity(103558.0000)
Title: NAM_FL-5-7.02017.02017.3
Data file NAM_FL-5-7.mgf

Click mouse within plot area to zoom in by factor of two about that point
Or, Plot from 200 to 2000 Da Full range

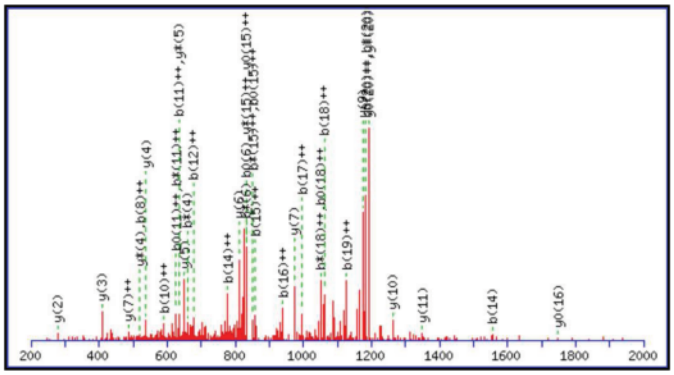

Monoisotopic mass of neutral peptide Mr(calc): 2527.9865
Fixed modifications: Carbamidomethyl (C)
Variable modifications:
D4 : Fluorene (DE)
Ions Score: 41 Expect: 4.3e-006
Matches (**Bold Red**): 40/218 fragment ions using 56 most intense peaks

#	b	b++	b*	b*++	b0	b0++	Seq.	y	y++	y*	y*++	y0	y0++	#
1	129.0659	65.0366	112.0393	56.5233			Q							21
2	266.1248	133.5660	249.0982	125.0527			H	2400.9352	1200.9712	2383.9087	**1192.4580**	2382.9246	**1191.9660**	20
3	397.1653	199.0863	380.1387	190.5730			M	2263.8763	1132.4418	2246.8497	1123.9285	2245.8657	1123.4365	19
4	676.2548	338.6310	**659.2283**	330.1178	658.2442	329.6258	D	2132.8358	1066.9215	2115.8093	1058.4083	2114.8252	1057.9163	18
5	763.2868	382.1471	746.2603	373.6338	745.2763	373.1418	S	1853.7463	927.3768	1836.7197	918.8635	1835.7357	918.3715	17
6	850.3189	425.6631	**833.2923**	417.1498	**832.3083**	416.6578	S	1766.7142	883.8608	1749.6877	875.3475	**1748.7037**	874.8555	16
7	951.3665	476.1869	934.3400	467.6736	933.3560	467.1816	T	1679.6822	840.3447	1662.6557	**831.8315**	1661.6716	**831.3395**	15
8	1038.3986	**519.7029**	1021.3720	511.1896	1020.3880	510.6976	S	1578.6345	789.8209	1561.6080	781.3076	1560.6240	780.8156	14
9	1109.4357	555.2215	1092.4091	546.7082	1091.4251	546.2162	A	1491.6025	746.3049	1474.5759	737.7916	1473.5919	737.2996	13
10	1180.4728	**590.7400**	1163.4462	582.2268	1162.4622	581.7348	A	1420.5654	710.7863	1403.5388	702.2731	1402.5548	701.7810	12
11	1267.5048	**634.2560**	1250.4783	**625.7428**	1249.4943	**625.2508**	S	**1349.5283**	675.2678	1332.5017	666.7545	1331.5177	666.2625	11
12	1354.5368	**677.7721**	1337.5103	669.2588	1336.5263	668.7668	S	**1262.4962**	631.7518	1245.4697	623.2385	1244.4857	622.7465	10
13	1441.5689	721.2881	1424.5423	712.7748	1423.5583	712.2828	S	**1175.4642**	588.2357	1158.4377	579.7225	1157.4536	579.2305	9
14	**1555.6118**	**778.3095**	1538.5853	769.7963	1537.6012	769.3043	N	1088.4322	544.7197	1071.4056	536.2065			8
15	1718.6751	**859.8412**	1701.6486	**851.3279**	1700.6646	**850.8359**	Y	**974.3893**	**487.6983**	957.3627	479.1850			7
16	1878.7058	**939.8565**	1861.6792	931.3433	1860.6952	930.8512	C	**811.3259**	406.1666	794.2994	397.6533			6
17	1992.7487	**996.8780**	1975.7222	988.3647	1974.7381	987.8727	N	**651.2953**	326.1513	**634.2687**	317.6380			5
18	2120.8073	**1060.9073**	2103.7807	**1052.3940**	2102.7967	**1051.9020**	Q	**537.2524**	269.1298	**520.2258**	260.6165			4
19	2251.8478	**1126.4275**	2234.8212	1117.9142	2233.8372	1117.4222	M	**409.1938**	205.1005	392.1672	196.5873			3
20	2382.8883	**1191.9478**	2365.8617	**1183.4345**	2364.8777	**1182.9425**	M	**278.1533**	139.5803	261.1267	131.0670			2
21							K	147.1128	74.0600	130.0863	65.5468			1

Figure S7. Fluorenyl ester of aspartic acid 14 of RNase A.

Mascot Search Results

Peptide View

MS/MS Fragmentation of CKPVNTFVHESLADVQAVCSQK
Found in gi|111141, RNase A 27-150

Match to Query 3883: 2680.274742 from(894.432190,3+) intensity(128761.0000)
Title: NAM_FL-5-7.03167.03167.3
Data file NAM_FL-5-7.mgf

Click mouse within plot area to zoom in by factor of two about that point
Or, Plot from 200 to 2000 Da Full range

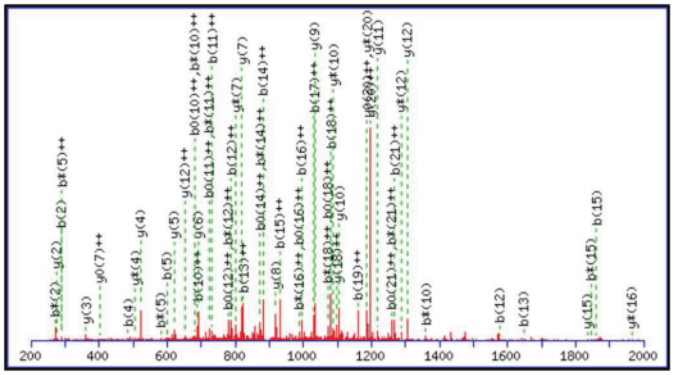

Monoisotopic mass of neutral peptide Mr(calc): 2680.2778
Fixed modifications: Carbamidomethyl (C)
Variable modifications:
E10 : Fluorene (DE)
Ions Score: 40 Expect: 0.00014
Matches (Bold Red): 59/236 fragment ions using 121 most intense peaks

#	b	b^{++}	b^{*}	b^{*++}	b^{0}	b^{0++}	Seq.	y	y^{++}	y^{*}	y^{*++}	y^{0}	y^{0++}	#
1	161.0379	81.0226					C							22
2	289.1329	145.0701	272.1063	136.5568			K	2521.2544	1261.1308	2504.2279	1252.6176	2503.2438	1252.1256	21
3	386.1857	193.5965	369.1591	185.0832			P	2393.1595	1197.0834	2376.1329	1188.5701	2375.1489	1188.0781	20
4	485.2541	243.1307	468.2275	234.6174			V	2296.1067	1148.5570	2279.0801	1140.0437	2278.0961	1139.5517	19
5	599.2970	300.1521	582.2704	291.6389			N	2197.0383	1099.0228	2180.0117	1090.5095	2179.0277	1090.0175	18
6	700.3447	350.6760	683.3181	342.1627	682.3341	341.6707	T	2082.9953	1042.0013	2065.9688	1033.4880	2064.9848	1032.9960	17
7	847.4131	424.2102	830.3865	415.6969	829.4025	415.2049	F	1981.9477	991.4775	1964.9211	982.9642	1963.9371	982.4722	16
8	946.4815	473.7444	929.4550	465.2311	928.4709	464.7391	V	1834.8793	917.9433	1817.8527	909.4300	1816.8687	908.9380	15
9	1083.5404	542.2738	1066.5139	533.7606	1065.5298	533.2686	H	1735.8108	868.4091	1718.7843	859.8958	1717.8003	859.4038	14
10	1376.6456	688.8264	1359.6191	680.3132	1358.6350	679.8212	E	1598.7519	799.8796	1581.7254	791.3663	1580.7414	790.8743	13
11	1463.6776	732.3425	1446.6511	723.8292	1445.6671	723.3372	S	1305.6467	653.3270	1288.6202	644.8137	1287.6362	644.3217	12
12	1576.7617	788.8845	1559.7352	780.3712	1558.7511	779.8792	L	1218.6147	609.8110	1201.5882	601.2977	1200.6041	600.8057	11
13	1647.7988	824.4030	1630.7723	815.8898	1629.7883	815.3978	A	1105.5306	553.2690	1088.5041	544.7557	1087.5201	544.2637	10
14	1762.8258	881.9165	1745.7992	873.4032	1744.8152	872.9112	D	1034.4935	517.7504	1017.4670	509.2371	1016.4830	508.7451	9
15	1861.8942	931.4507	1844.8676	922.9375	1843.8836	922.4454	V	919.4666	460.2369	902.4400	451.7237	901.4560	451.2316	8
16	1989.9528	995.4800	1972.9262	986.9667	1971.9422	986.4747	Q	820.3982	410.7027	803.3716	402.1894	802.3876	401.6974	7
17	2060.9899	1030.9986	2043.9633	1022.4853	2042.9793	1021.9933	A	692.3396	346.6734	675.3130	338.1602	674.3290	337.6681	6
18	2160.0583	1080.5328	2143.0317	1072.0195	2142.0477	1071.5275	V	621.3025	311.1549	604.2759	302.6416	603.2919	302.1496	5
19	2320.0889	1160.5481	2303.0624	1152.0348	2302.0784	1151.5428	C	522.2341	261.6207	505.2075	253.1074	504.2235	252.6154	4
20	2407.1210	1204.0641	2390.0944	1195.5508	2389.1104	1195.0588	S	362.2034	181.6053	345.1769	173.0921	344.1928	172.6001	3
21	2535.1795	1268.0934	2518.1530	1259.5801	2517.1690	1259.0881	Q	275.1714	138.0893	258.1448	129.5761			2
22							K	147.1128	74.0600	130.0863	65.5468			1

Figure S8. Fluorenyl ester of glutamic acid 49 of RNase A.

Mascot Search Results

Peptide View

MS/MS Fragmentation of HIIVACEGNPYVPVHFDASV
Found in gi|111141, RNase A 27-150

Match to Query 3066: 2387.140830 from(796.720886,3+) intensity(37095.2000)
Title: NAM_FL-5-7.03000.03000.3
Data file NAM_FL-5-7.mgf

Click mouse within plot area to zoom in by factor of two about that point
Or, Plot from 200 to 2000 Da Full range

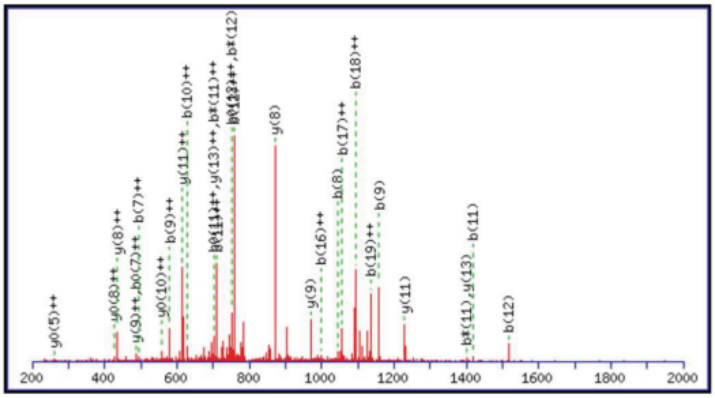

Monoisotopic mass of neutral peptide Mr(calc): 2387.1409
Fixed modifications: Carbamidomethyl (C)
Variable modifications:
E7 : Fluorene (DE)
Ions Score: 33 Expect: 0.001
Matches (Bold Red): 30/176 fragment ions using 68 most intense peaks

#	b	b^{++}	b*	b^{*++}	b^0	b^{0++}	Seq.	y	y^{++}	y*	y^{*++}	y^0	y^{0++}	#
1	138.0662	69.5367					H							20
2	251.1503	126.0788					I	2251.0892	1126.0483	2234.0627	1117.5350	2233.0787	1117.0430	19
3	364.2343	182.6208					I	2138.0052	1069.5062	2120.9786	1060.9930	2119.9946	1060.5009	18
4	463.3027	232.1550					V	2024.9211	1012.9642	2007.8946	1004.4509	2006.9106	1003.9589	17
5	534.3398	267.6736					A	1925.8527	963.4300	1908.8262	954.9167	1907.8421	954.4247	16
6	694.3705	347.6889					C	1854.8156	927.9114	1837.7890	919.3982	1836.8050	918.9062	15
7	987.4757	**494.2415**			969.4651	**485.2362**	E	1694.7849	847.8961	1677.7584	839.3828	1676.7744	838.8908	14
8	**1044.4972**	522.7522			1026.4866	513.7469	G	**1401.6797**	**701.3435**	1384.6532	692.8302	1383.6692	692.3382	13
9	**1158.5401**	**579.7737**	1141.5135	571.2604	1140.5295	570.7684	N	1344.6583	672.8328	1327.6317	664.3195	1326.6477	663.8275	12
10	1255.5928	**628.3001**	1238.5663	619.7868	1237.5823	619.2948	P	**1230.6154**	**615.8113**			1212.6048	606.8060	11
11	**1418.6562**	**709.8317**	**1401.6296**	**701.3185**	1400.6456	**700.8264**	Y	1133.5626	567.2849			1115.5520	**558.2796**	10
12	**1517.7246**	**759.3659**	1500.6980	**750.8527**	1499.7140	**750.3607**	V	**970.4993**	**485.7533**			952.4887	476.7480	9
13	1614.7774	807.8923	1597.7508	799.3790	1596.7668	798.8870	P	**871.4308**	**436.2191**			853.4203	**427.2138**	8
14	1713.8458	857.4265	1696.8192	848.9132	1695.8352	848.4212	V	774.3781	387.6927			756.3675	378.6874	7
15	1850.9047	925.9560	1833.8781	917.4427	1832.8941	916.9507	H	675.3097	338.1585			657.2991	329.1532	6
16	1997.9731	**999.4902**	1980.9465	990.9769	1979.9625	990.4849	F	538.2508	269.6290			520.2402	**260.6237**	5
17	2113.0000	**1057.0037**	2095.9735	1048.4904	2094.9895	1047.9984	D	391.1823	196.0948			373.1718	187.0895	4
18	2184.0372	**1092.5222**	2167.0106	1084.0089	2166.0266	1083.5169	A	276.1554	138.5813			258.1448	129.5761	3
19	2271.0692	**1136.0382**	2254.0426	1127.5250	2253.0586	1127.0329	S	205.1183	103.0628			187.1077	94.0575	2
20							V	118.0863	59.5468					1

Figure S9. Fluorenyl ester of glutamic acid 111 of RNase A.

Mascot Search Results

Peptide View

MS/MS Fragmentation of HIIVACEGNPYVPVHFDASV
Found in gi|111141, RNase A 27-150

Match to Query 3060: 2387.138817 from(796.720215,3+) intensity(54131.1000)
Title: NAM_FL-5-7.03201.03201.3
Data file NAM_FL-5-7.mgf

Click mouse within plot area to zoom in by factor of two about that point
Or, Plot from 200 to 2000 Da Full range

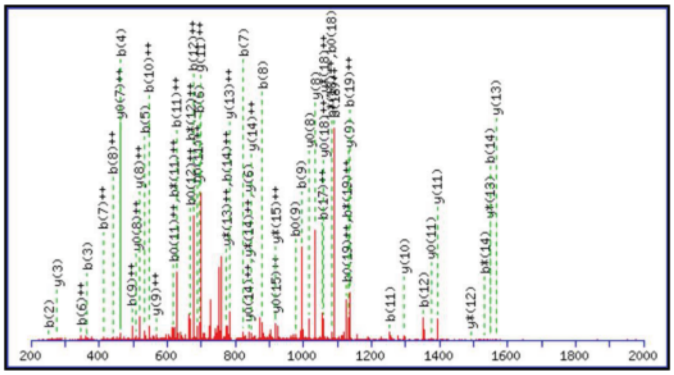

Monoisotopic mass of neutral peptide Mr(calc): 2387.1409
Fixed modifications: Carbamidomethyl (C)
Variable modifications:
D17 : Fluorene (DE)
Ions Score: 47 Expect: 3.9e-005
Matches (Bold Red): 58/176 fragment ions using 132 most intense peaks

#	b	b^{++}	b*	b^{*++}	b^0	b^{0++}	Seq.	y	y^{++}	y*	y^{*++}	y^0	y^{0++}	#
1	138.0662	69.5367					H							20
2	251.1503	126.0788					I	2251.0892	1126.0483	2234.0627	1117.5350	2233.0787	1117.0430	19
3	364.2343	182.6208					I	2138.0052	1069.5062	2120.9786	1060.9930	2119.9946	1060.5009	18
4	463.3027	232.1550					V	2024.9211	1012.9642	2007.8946	1004.4509	2006.9106	1003.9589	17
5	534.3398	267.6736					A	1925.8527	963.4300	1908.8262	954.9167	1907.8421	954.4247	16
6	694.3705	347.6889					C	1854.8156	927.9114	1837.7890	919.3982	1836.8050	918.9062	15
7	823.4131	412.2102			805.4025	403.2049	E	1694.7849	847.8961	1677.7584	839.3828	1676.7744	838.8908	14
8	880.4346	440.7209			862.4240	431.7156	G	1565.7423	783.3748	1548.7158	774.8615	1547.7318	774.3695	13
9	994.4775	497.7424	977.4509	489.2291	976.4669	488.7371	N	1508.7209	754.8641	1491.6943	746.3508	1490.7103	745.8588	12
10	1091.5302	546.2688	1074.5037	537.7555	1073.5197	537.2635	P	1394.6780	697.8426			1376.6674	688.8373	11
11	1254.5936	627.8004	1237.5670	619.2871	1236.5830	618.7951	Y	1297.6252	649.3162			1279.6146	640.3110	10
12	1353.6620	677.3346	1336.6354	668.8214	1335.6514	668.3293	V	1134.5619	567.7846			1116.5513	558.7793	9
13	1450.7147	725.8610	1433.6882	717.3477	1432.7042	716.8557	P	1035.4935	518.2504			1017.4829	509.2451	8
14	1549.7832	775.3952	1532.7566	766.8819	1531.7726	766.3899	V	938.4407	469.7240			920.4301	460.7187	7
15	1686.8421	843.9247	1669.8155	835.4114	1668.8315	834.9194	H	839.3723	420.1898			821.3617	411.1845	6
16	1833.9105	917.4589	1816.8839	908.9456	1815.8999	908.4536	F	702.3134	351.6603			684.3028	342.6550	5
17	2113.0000	1057.0037	2095.9735	1048.4904	2094.9895	1047.9984	D	555.2449	278.1261			537.2344	269.1208	4
18	2184.0372	1092.5222	2167.0106	1084.0089	2166.0266	1083.5169	A	276.1554	138.5813			258.1448	129.5761	3
19	2271.0692	1136.0382	2254.0426	1127.5250	2253.0586	1127.0329	S	205.1183	103.0628			187.1077	94.0575	2
20							V	118.0863	59.5468					1

Figure S10. Fluorenyl ester of aspartic acid 121 of RNase A.

9. MALDI–TOF Mass Spectrometry Data for FLAG–RNase A-Esterification Experiments

A. Untreated FLAG–RNase A (expected *m*/*z* 14816)

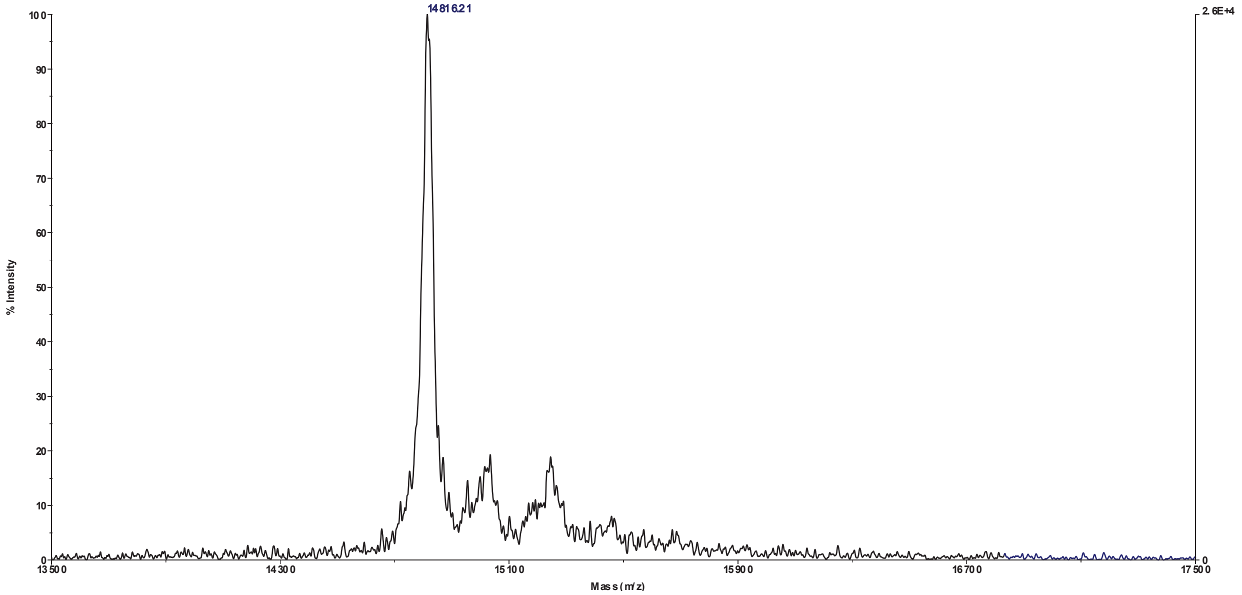

B. FLAG–RNase A esterified with diazofluorene (**2**) (10 equiv) (expected *m*/*z* 14816 + 164n)

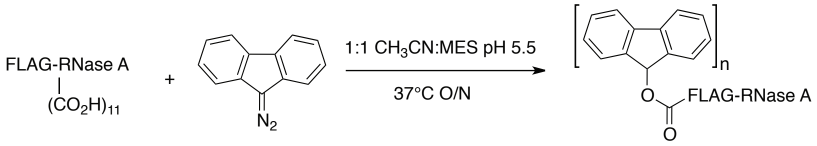

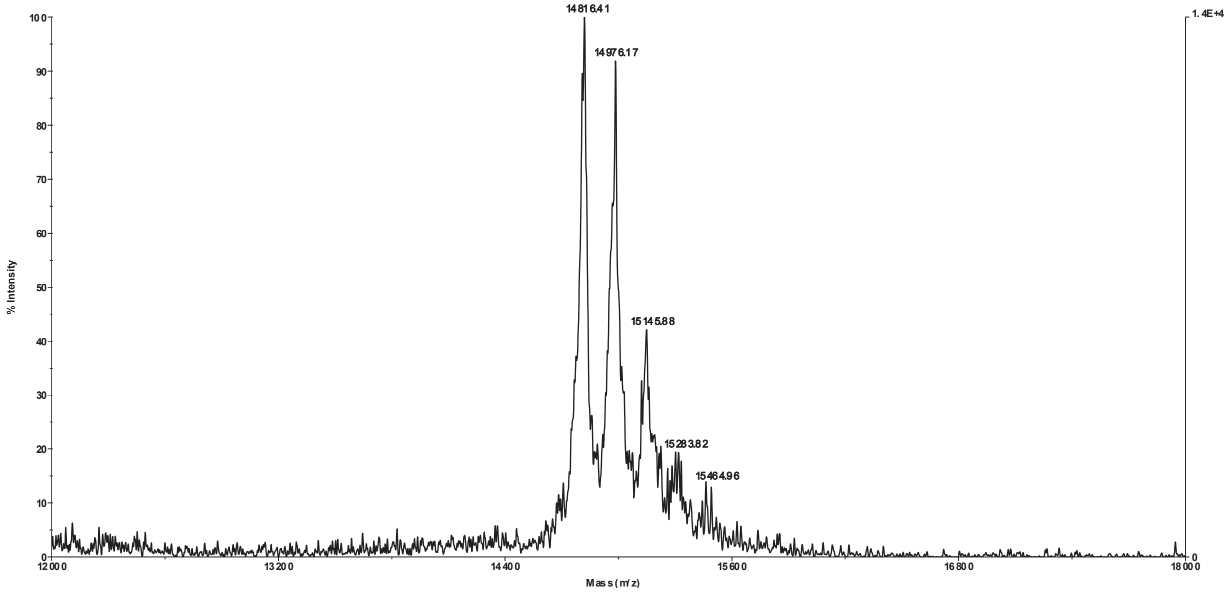

C. FLAG–RNase A-esterification with diazofluorene (**2**) (200 equiv) (expected *m*/*z* 14816 + 164n)

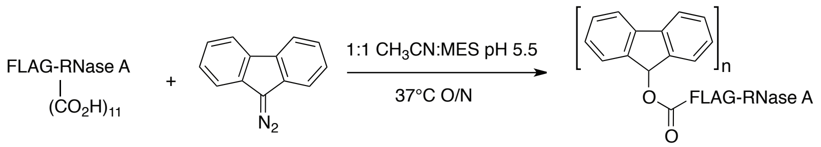

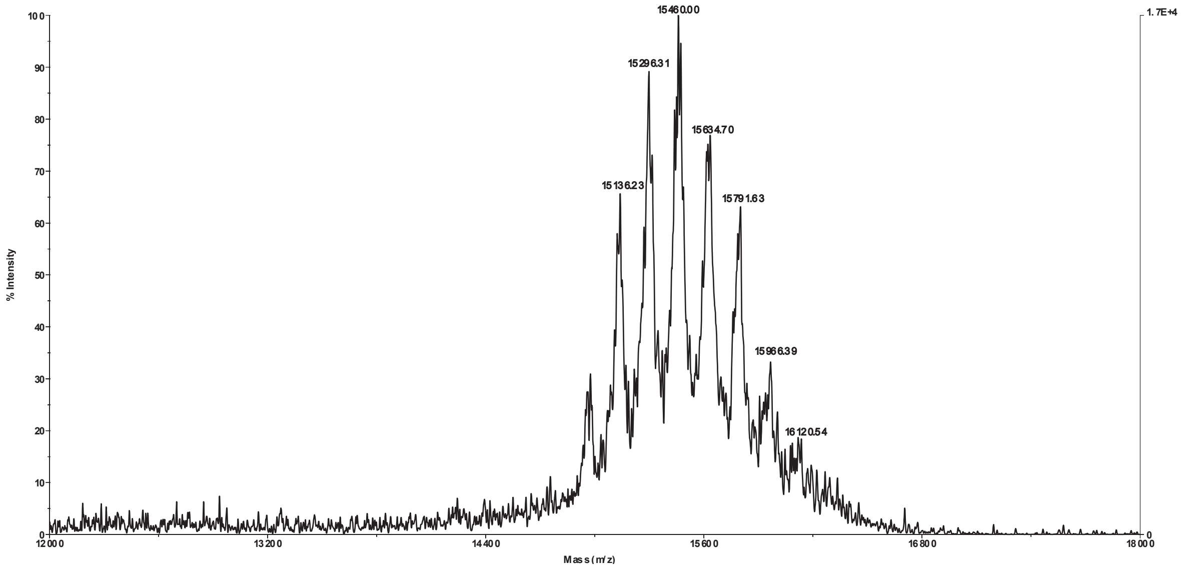

Figure S11. MALDI–TOF mass spectra of untreated FLAG–RNase A and FLAG–RNase A treated with diazofluorene (**2**).

A. HeLa cell lysate-treated FLAG–RNase A esterified with diazofluorene (**2**) (10 equiv) (expected *m*/*z* 14816)

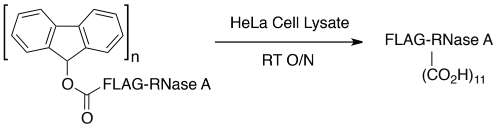

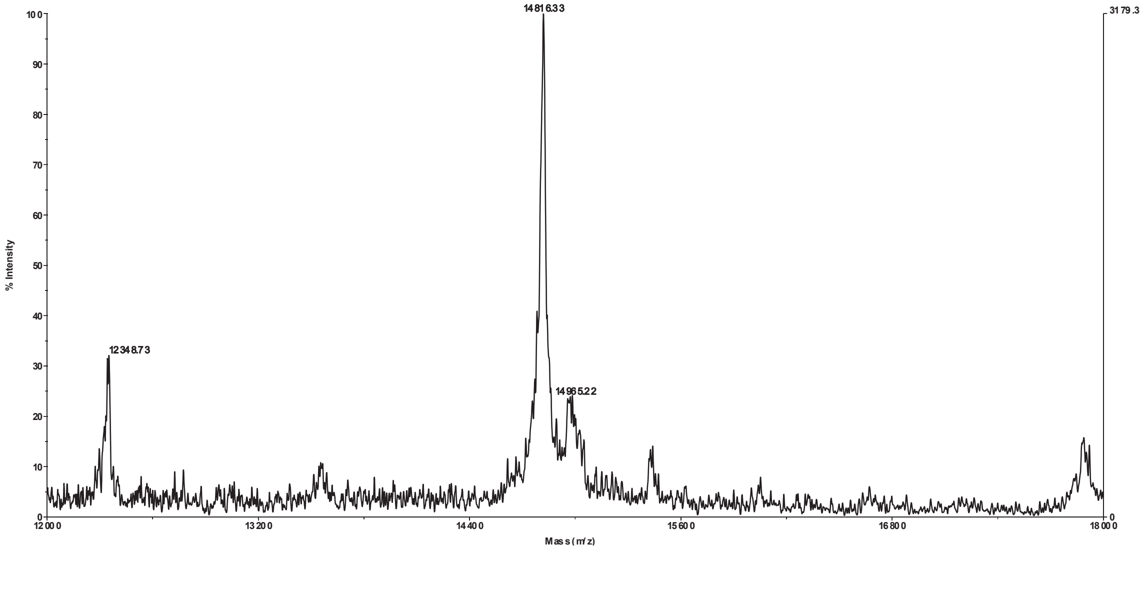

B. HeLa cell lysate-treated FLAG–RNase A esterified with diazofluorene (**2**) (200 equiv) (expected *m*/*z* 14816)

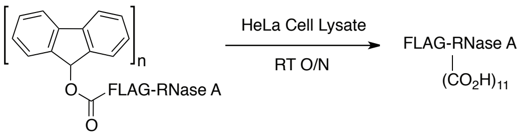

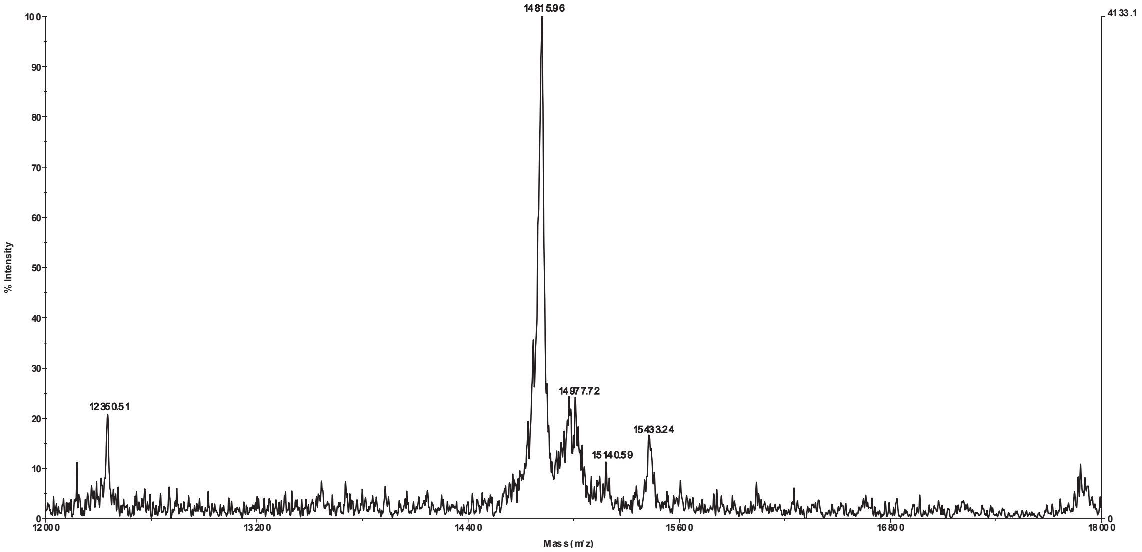

Figure S12. MALDI–TOF mass spectra of HeLa cell lysate-treated esterified FLAG–RNase A showing hydrolysis of all esters.

10. Ribonucleolytic Activity Assay Data

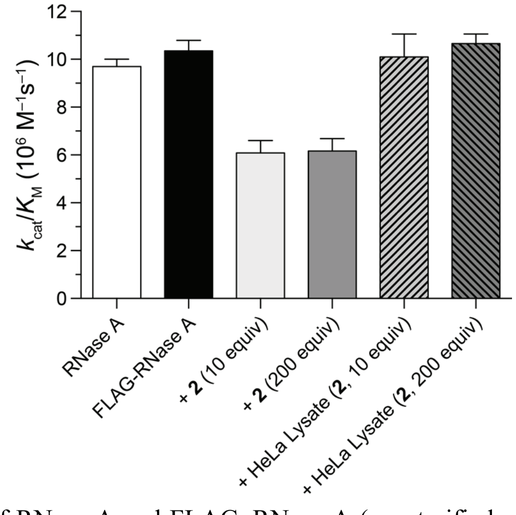

Figure S13. Enzymatic activity of RNase A and FLAG–RNase A (unesterified or esterified). When esterified with either a 10- or 200-fold molar excess of diazofluorene (**2**), the ribonucleolytic activity of FLAG–RNase A is decreased by ~50%. Upon exposure to a HeLa cell lysate and subsequent purification via the FLAG tag, the enzymatic activity is restored to original levels.

11. MALDI–TOF Mass Spectrometry Data for His_6–RFP-Esterification Experiments

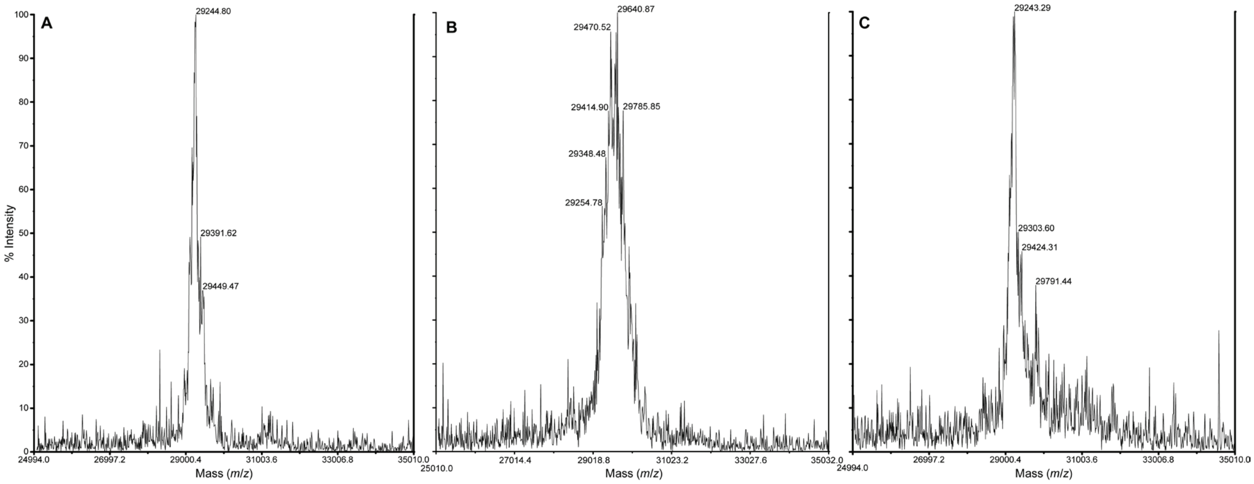

Figure S14. MALDI–TOF mass spectra. (A) Untreated His_6–RFP (expected *m*/*z* 29244). (B) His_6–RFP treated with diazofluorene (**2**) (10 equiv) (expected *m*/*z* 29244 + 164n). (C) HeLa cell lysate-treated esterified His_6–RFP (expected *m*/*z* 29244) showing hydrolysis of all esters.

12. References

1. P. A. Leland, L. W. Schultz, B.-M. Kim and R. T. Raines, *Proc. Natl. Acad. Sci. USA*, 1998, **95**, 10407-10412.
2. B. R. Kelemen, T. A. Klink, M. A. Behlke, S. R. Eubanks, P. A. Leland and R. T. Raines, *Nucleic Acids Res.*, 1999, **27**, 3696-3701.
3. N. C. Shaner, R. E. Campbell, P. A. Steinbach, B. N. G. Giepmans, A. E. Palmer and R. Y. Tsien, *Nat. Biotechnol.*, **22**, 1567-1572.

13. NMR Spectra (All compounds were dissolved in $CDCl_3$ unless indicated otherwise.)

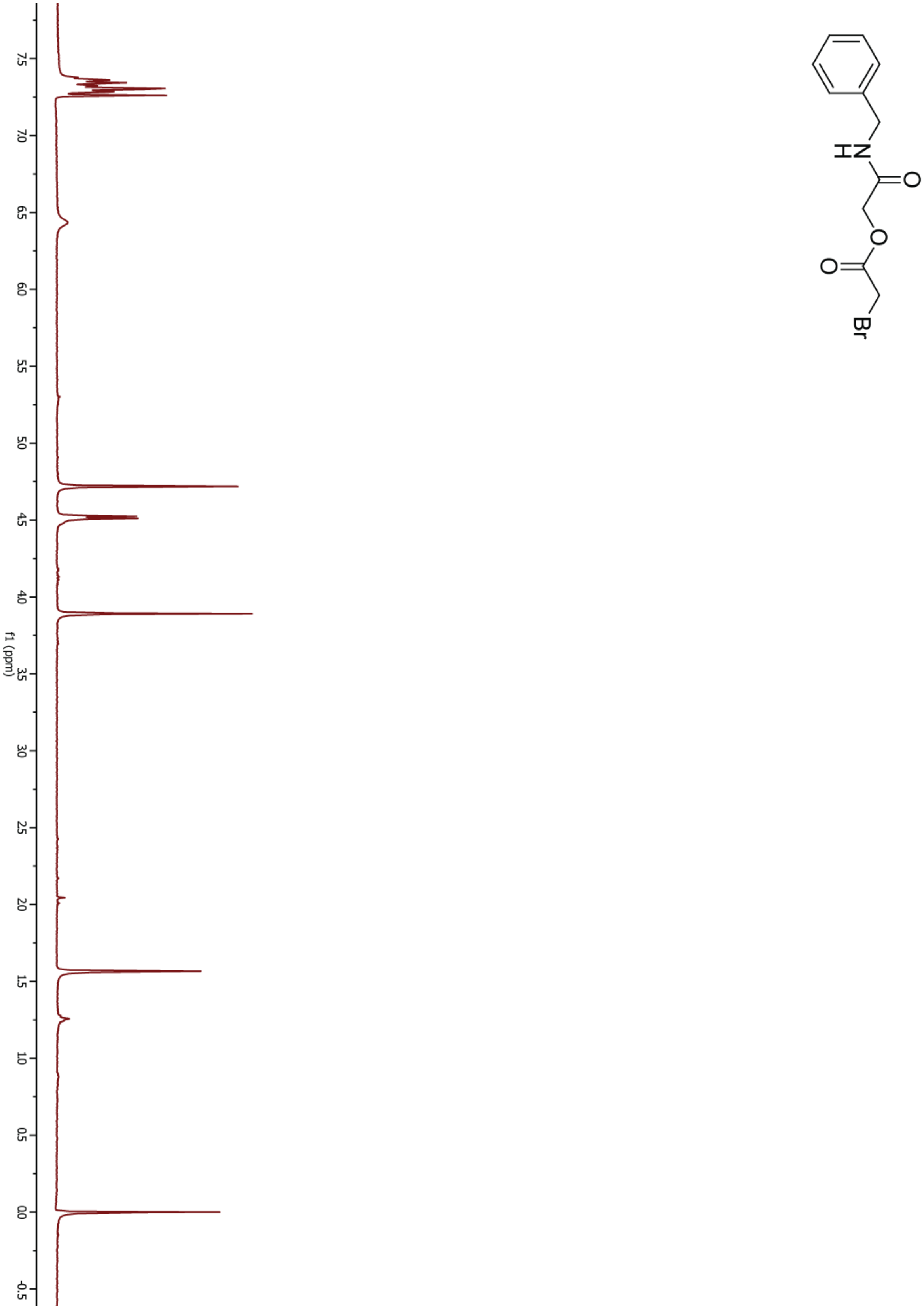

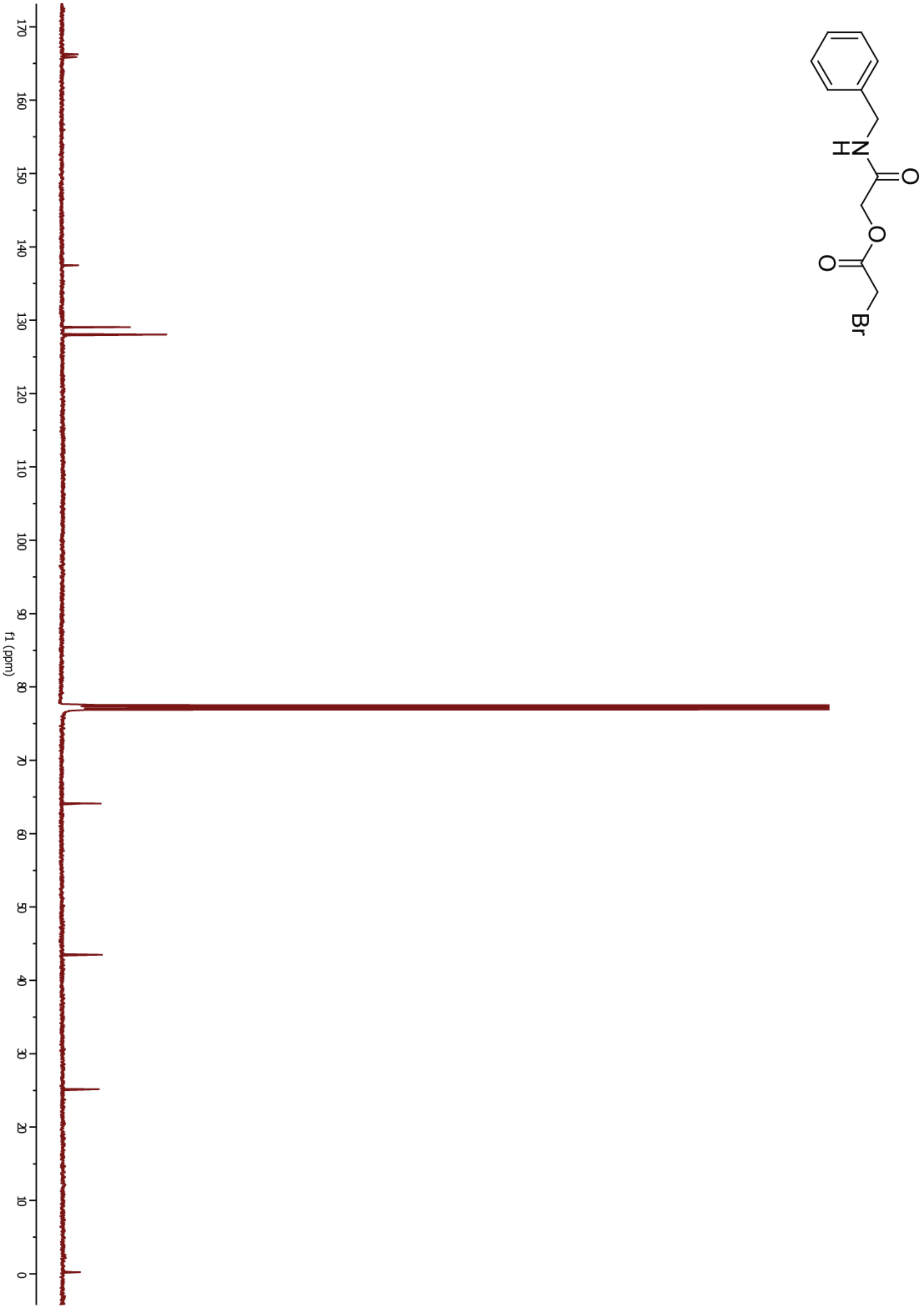
170
160
150
140
130
120
110
100
90
80
70
60
50
40
30
20
10
0
f1 (ppm)
N
H
O
O
O
Br

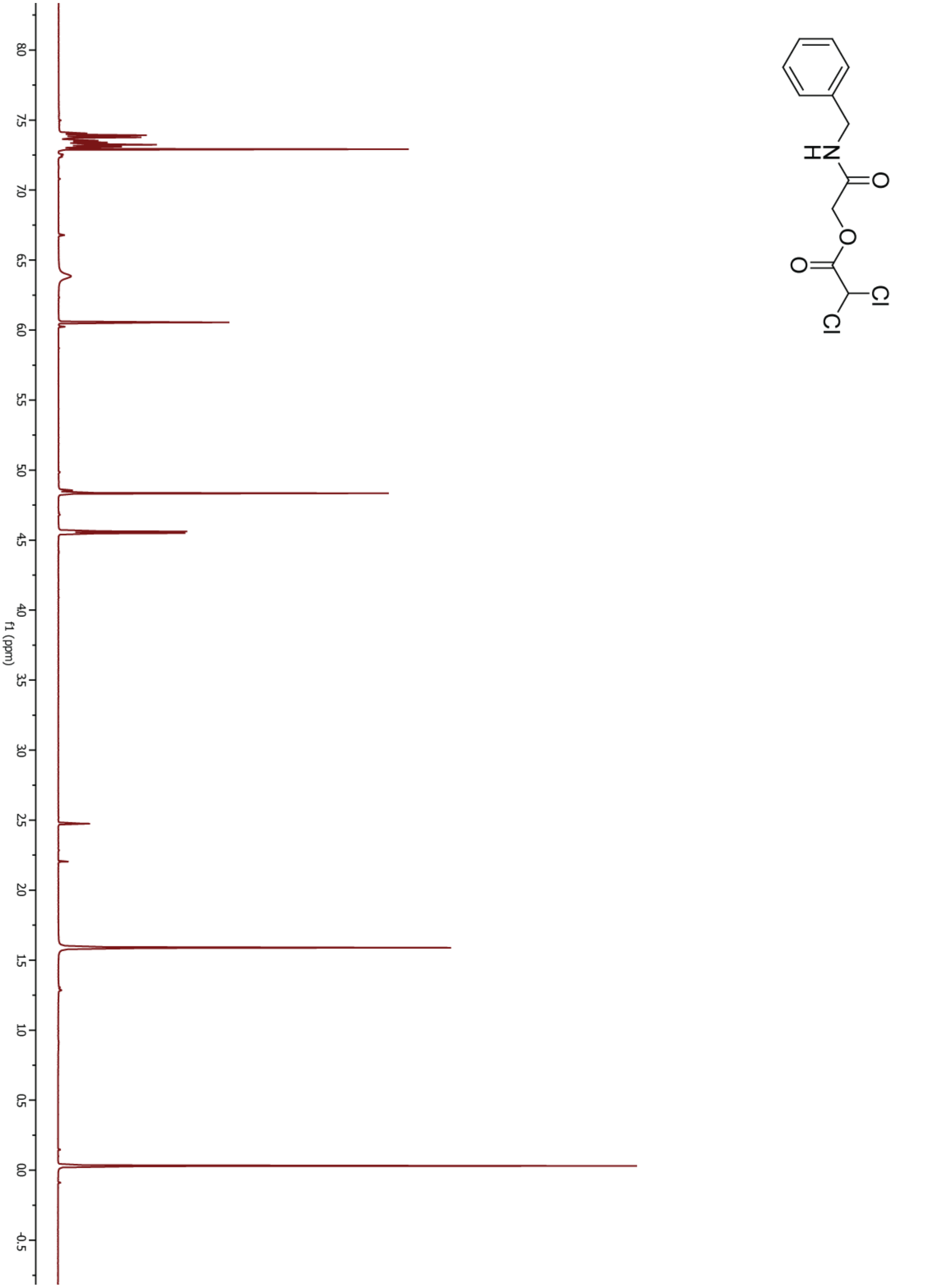
f1 (ppm)
8.0
7.5
7.0
6.5
6.0
5.5
5.0
4.5
4.0
3.5
3.0
2.5
2.0
1.5
1.0
0.5
0.0
-0.5
NH
O
O
O
Cl
Cl

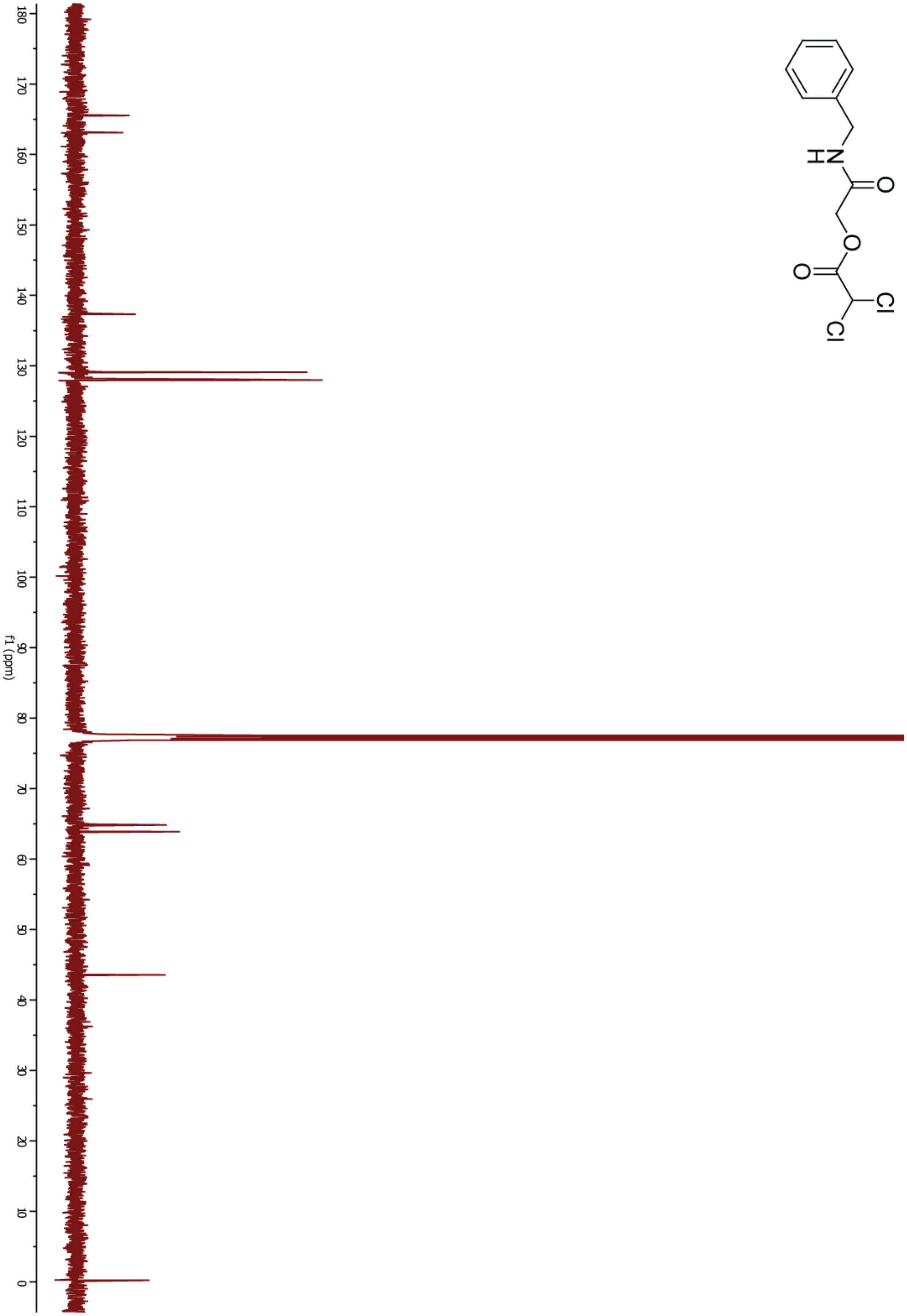
180
170
160
150
140
130
120
110
100
90
80
70
60
50
40
30
20
10
0
f1 (ppm)
N
H
O
O
O
Cl
Cl

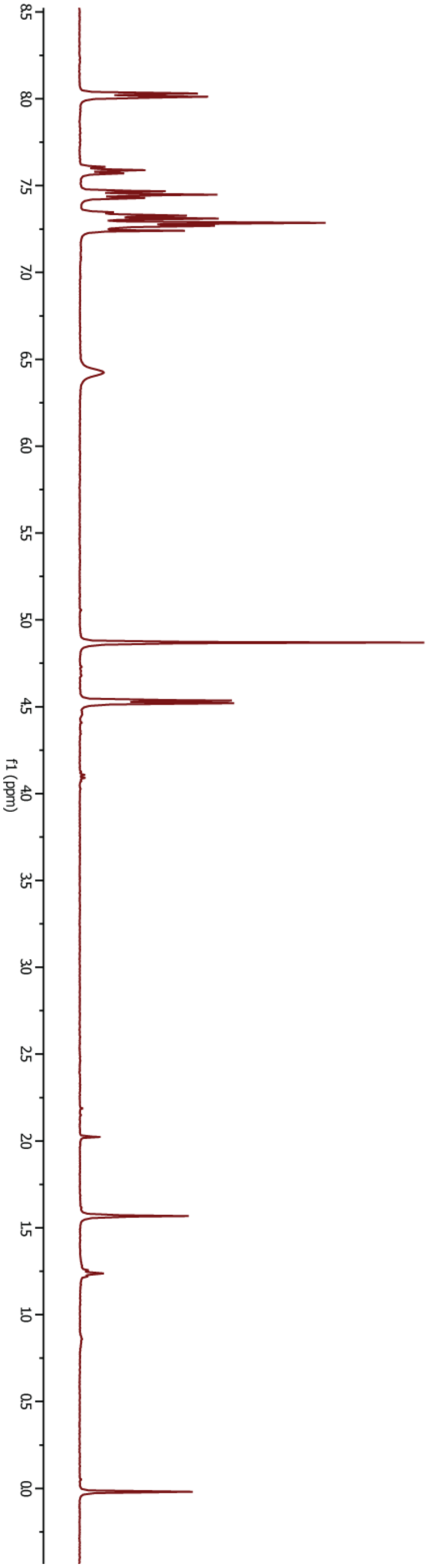
8.5
8.0
7.5
7.0
6.5
6.0
5.5
5.0
4.5
4.0
3.5
3.0
2.5
2.0
1.5
1.0
0.5
0.0
f1 (ppm)

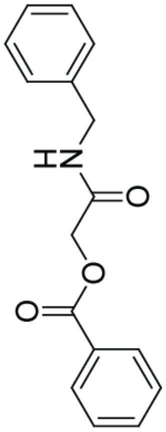
HN
O
O
O

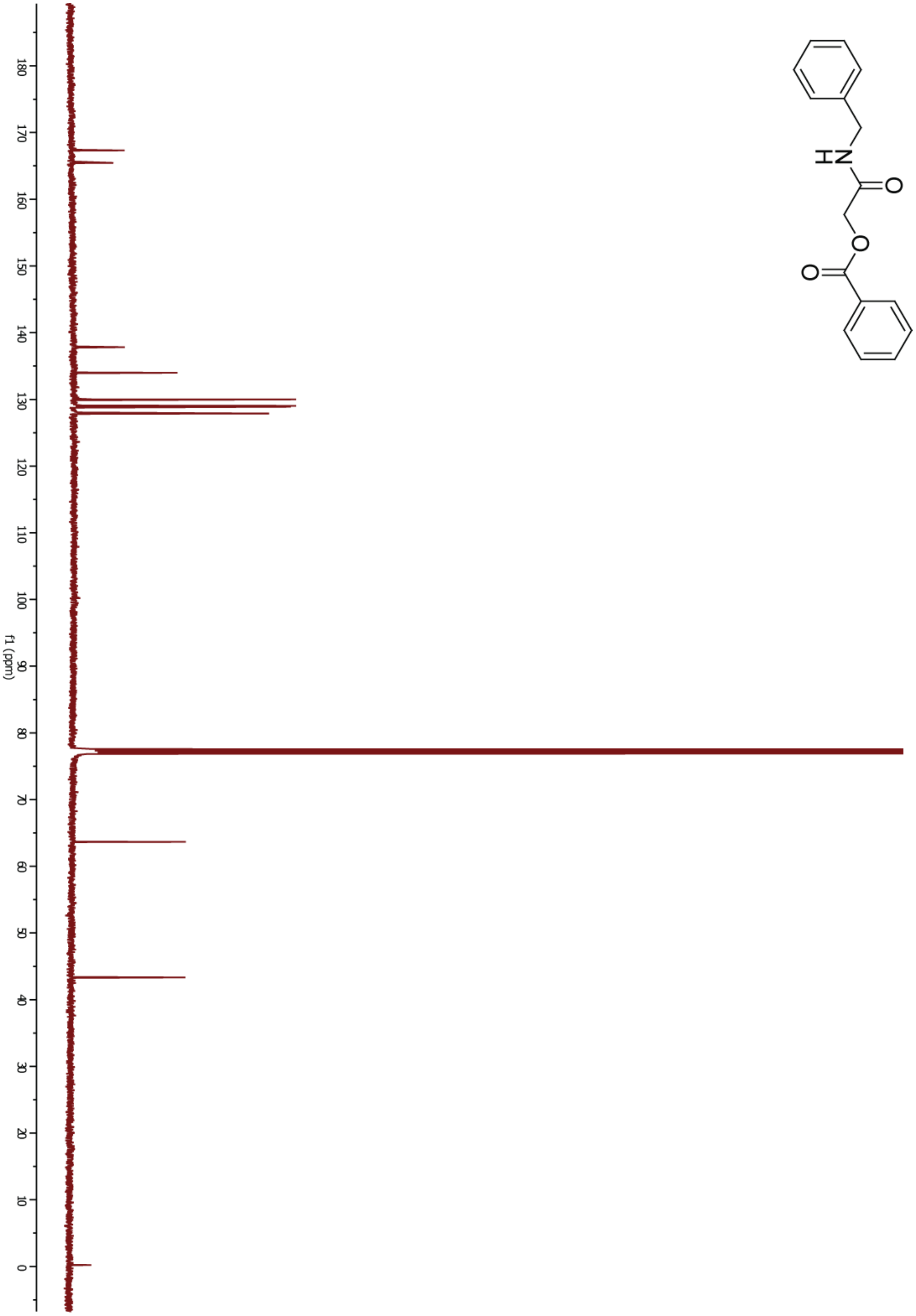
180
170
160
150
140
130
120
110
100
90
80
70
60
50
40
30
20
10
0
f1 (ppm)
HN
O
O
O

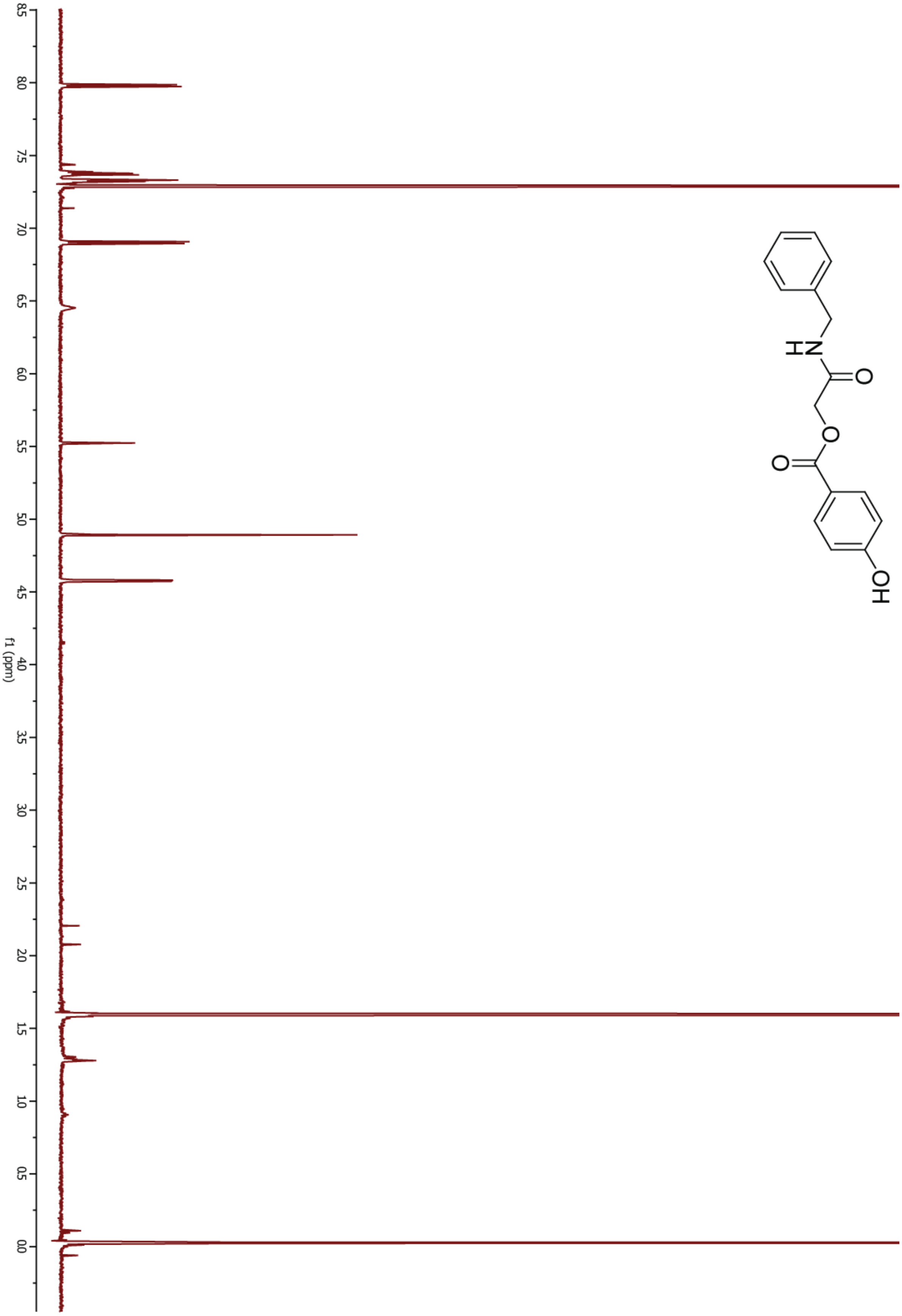
f1 (ppm)
OH
O
O
NH
O

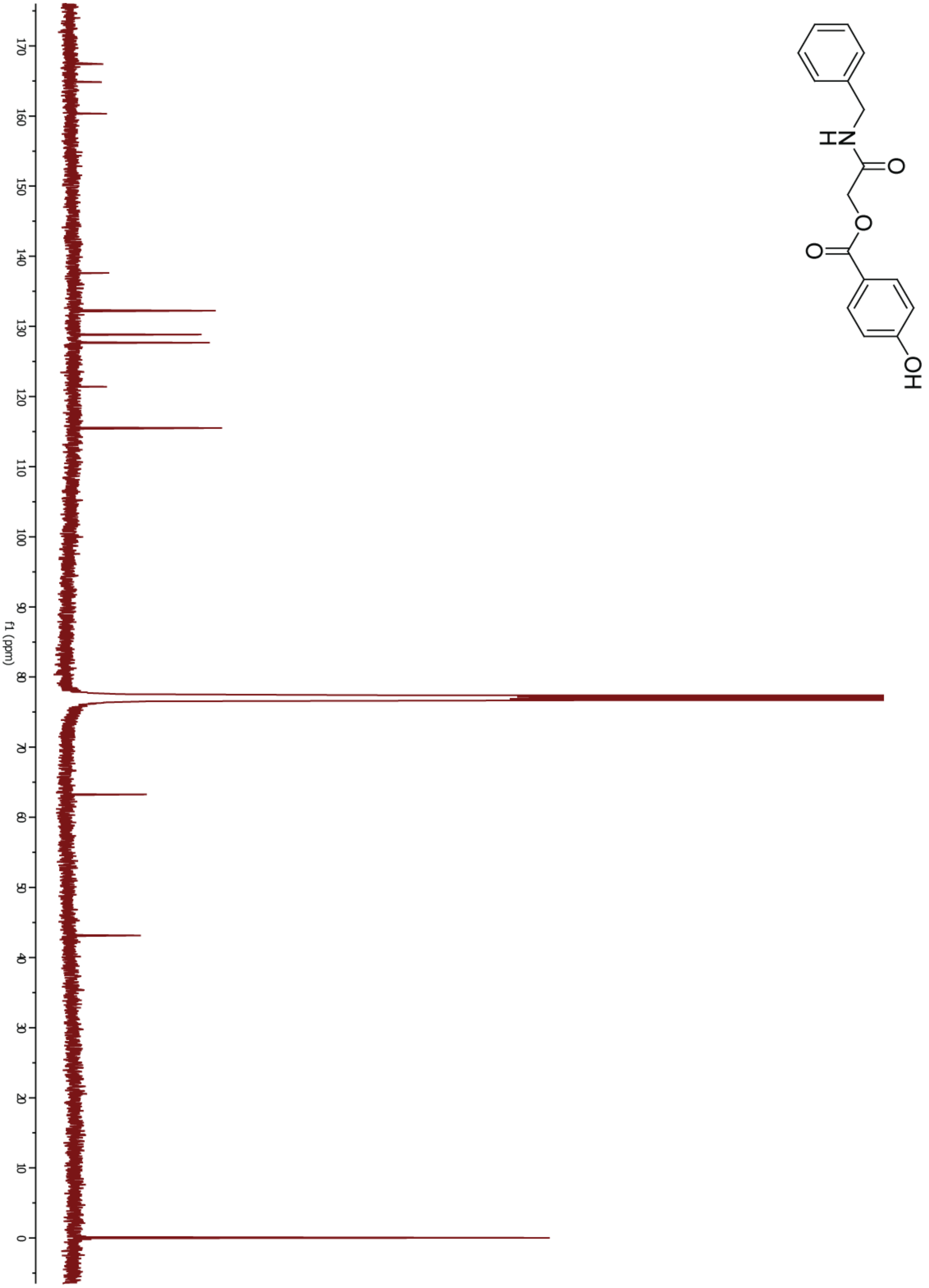
170
160
150
140
130
120
110
100
90
f1 (ppm)
80
70
60
50
40
30
20
10
0
HN
O
O
O
OH

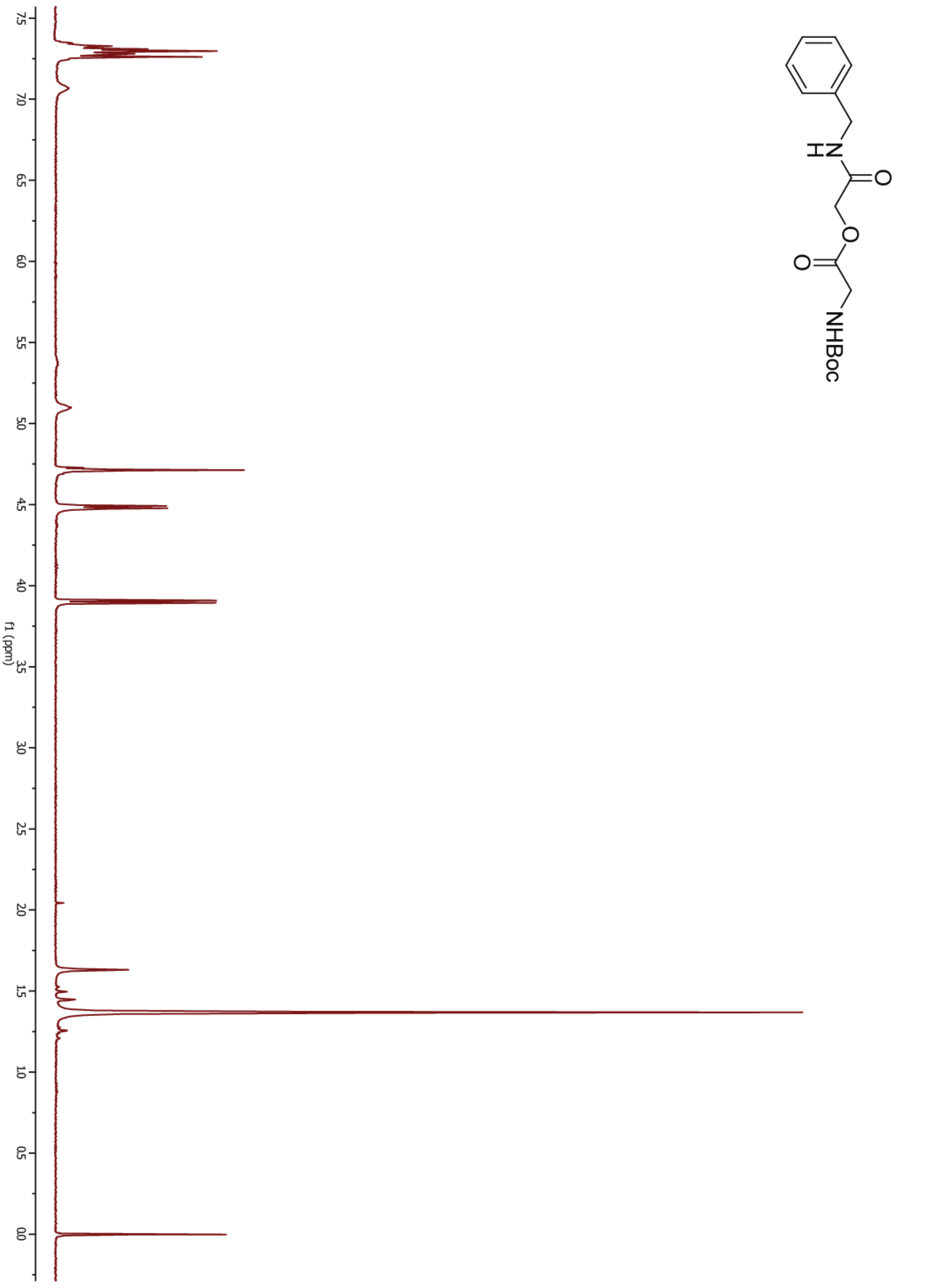
f1 (ppm)
NHBoc

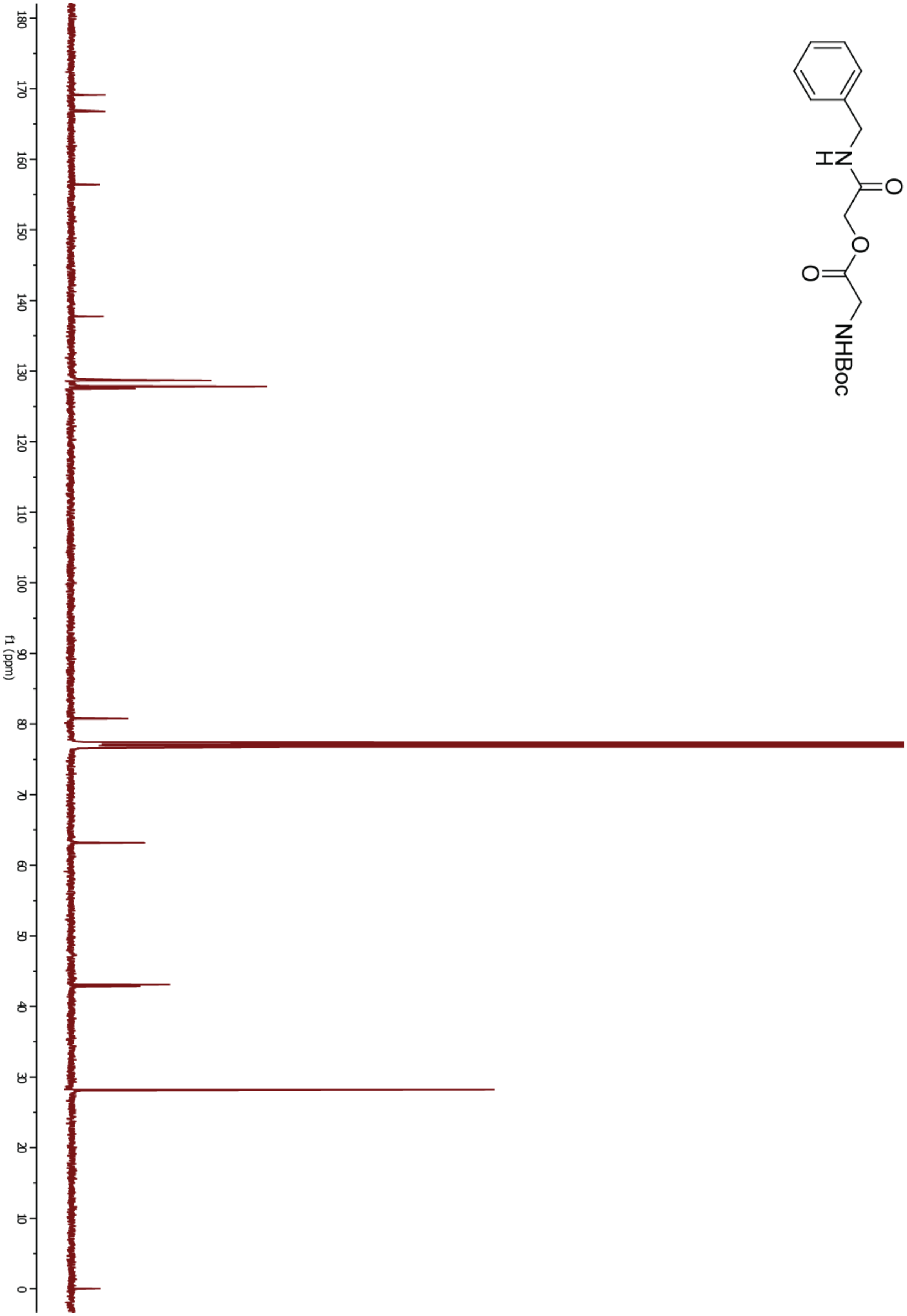
NHBoc
f1 (ppm)
180
170
160
150
140
130
120
110
100
90
80
70
60
50
40
30
20
10
0

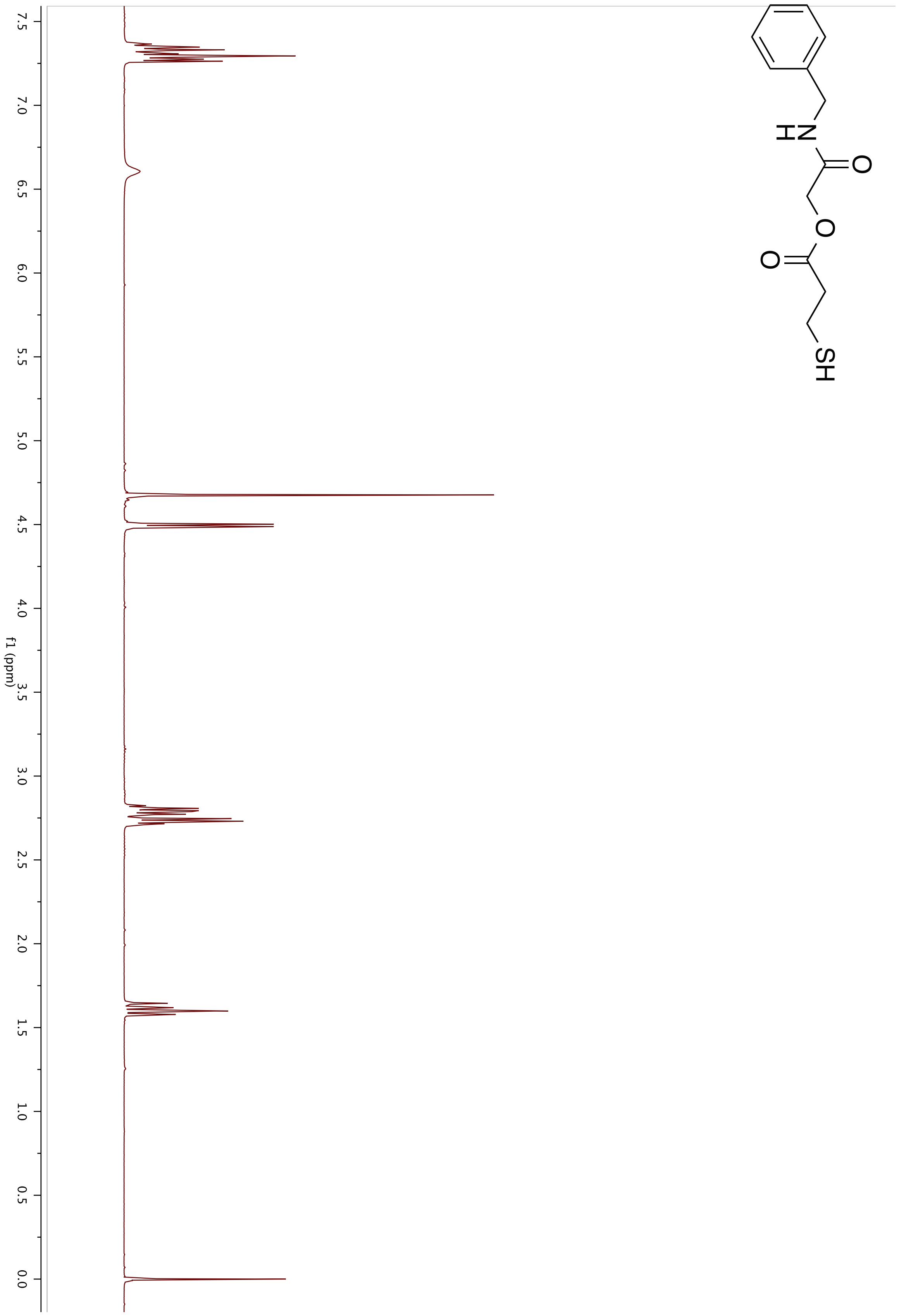
f1 (ppm)
SH
HN
O
O
O

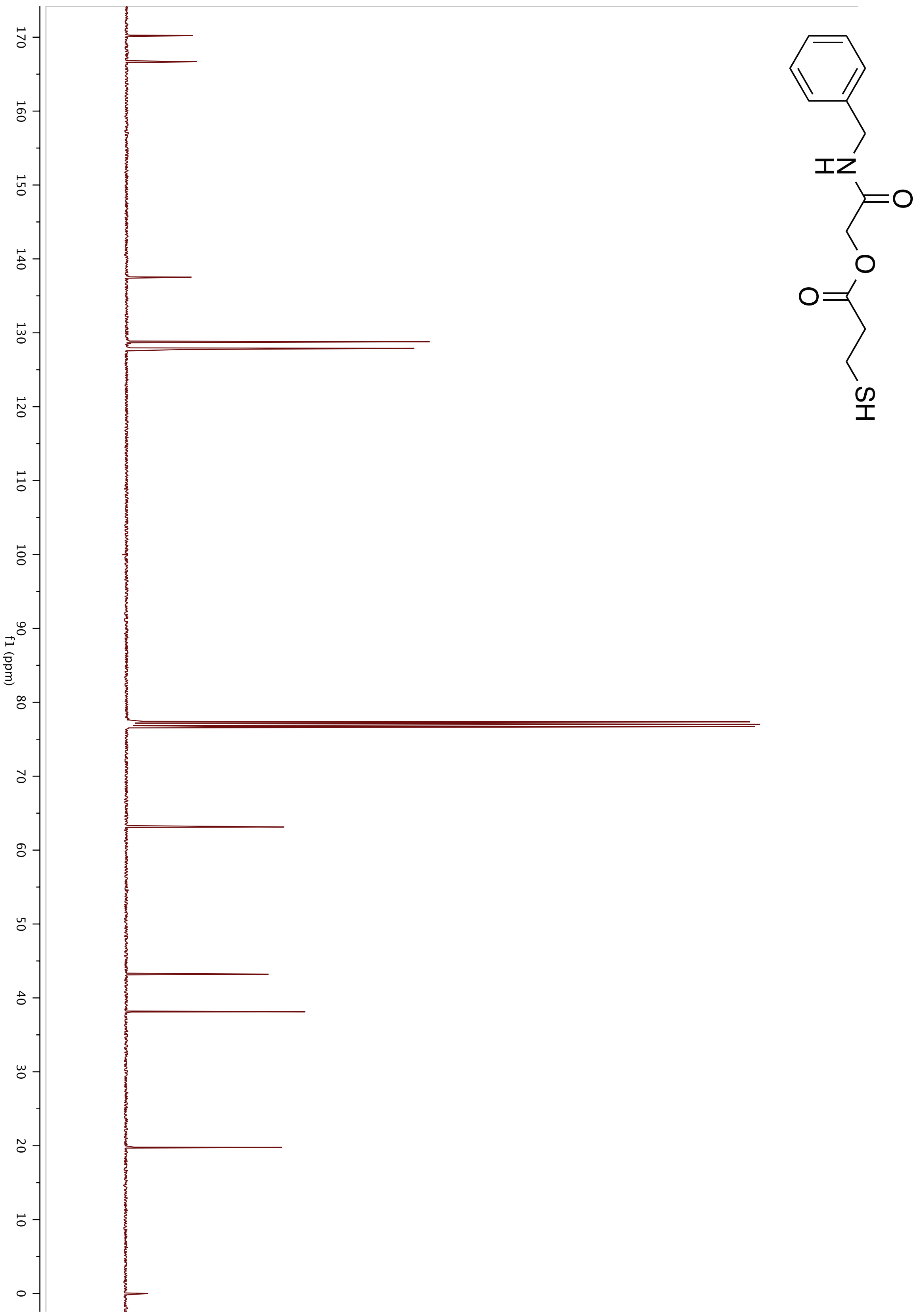

170
160
150
140
130
120
110
100
90
f1 (ppm)
80
70
60
50
40
30
20
10
0

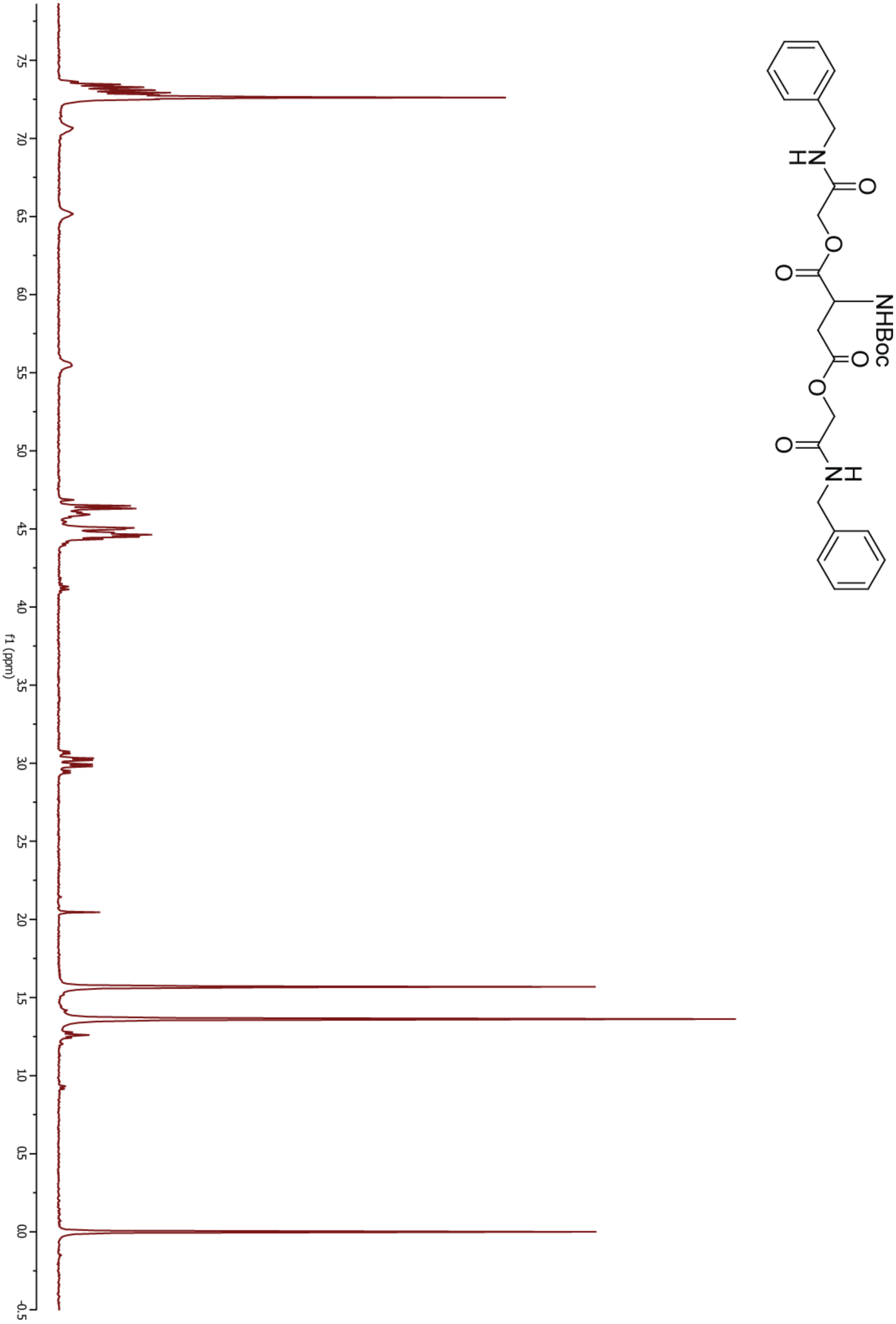

f1 (ppm)
7.5
7.0
6.5
6.0
5.5
5.0
4.5
4.0
3.5
3.0
2.5
2.0
1.5
1.0
0.5
0.0
-0.5
NHBoc

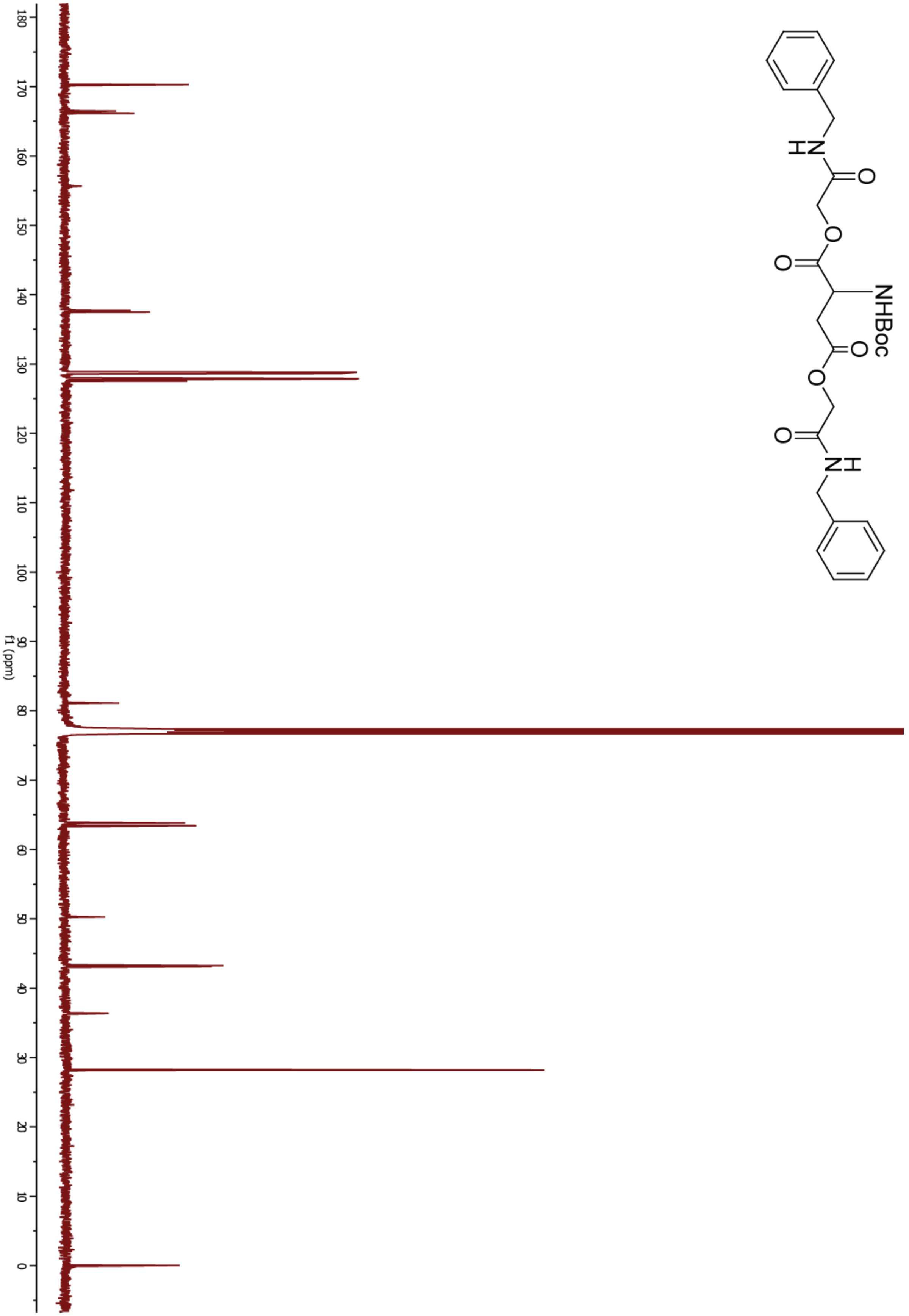
NHBoc
f1 (ppm)
180
170
160
150
140
130
120
110
100
90
80
70
60
50
40
30
20
10
0

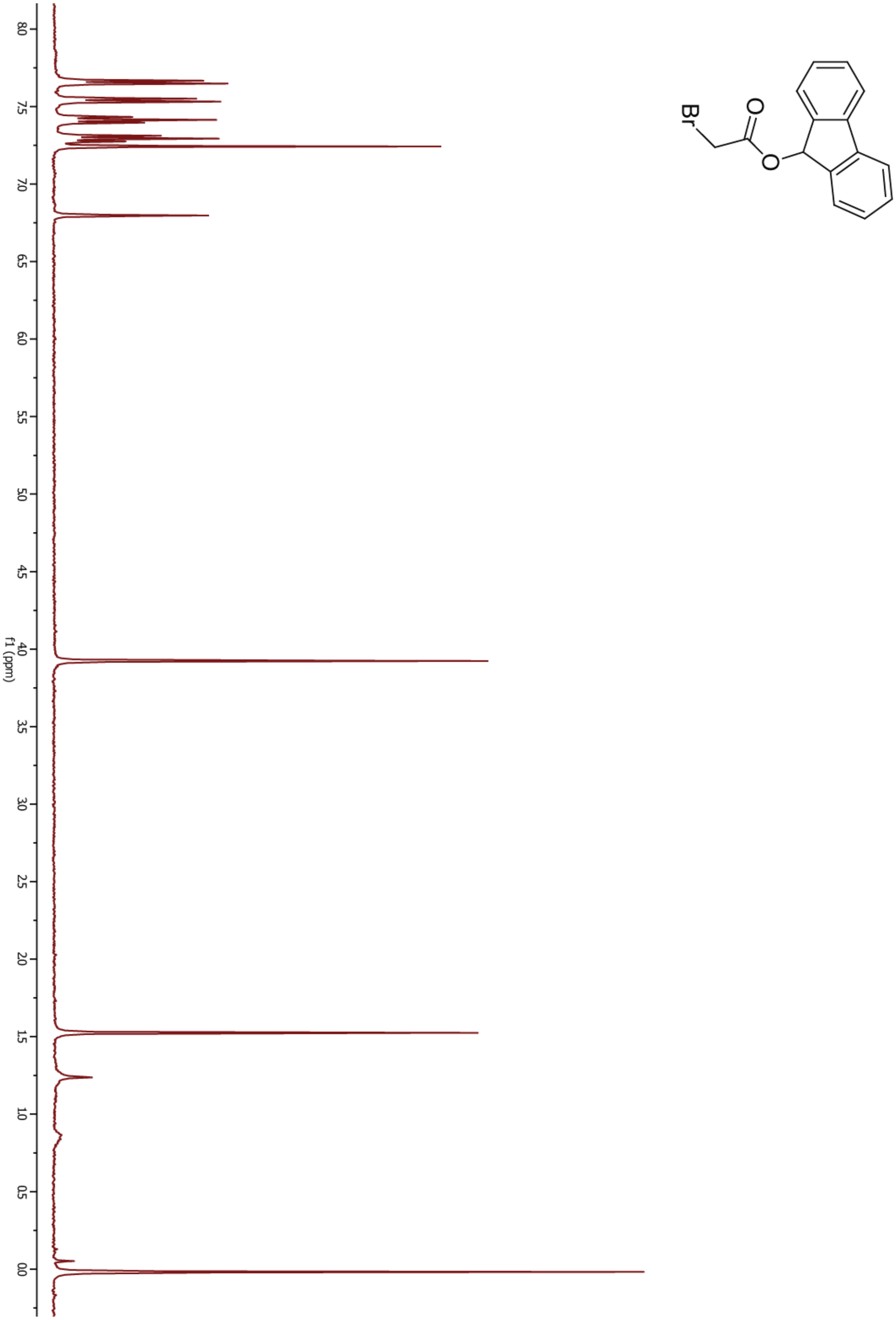
8.0
7.5
7.0
6.5
6.0
5.5
5.0
4.5
4.0
f1 (ppm)
3.5
3.0
2.5
2.0
1.5
1.0
0.5
0.0
Br
O
O

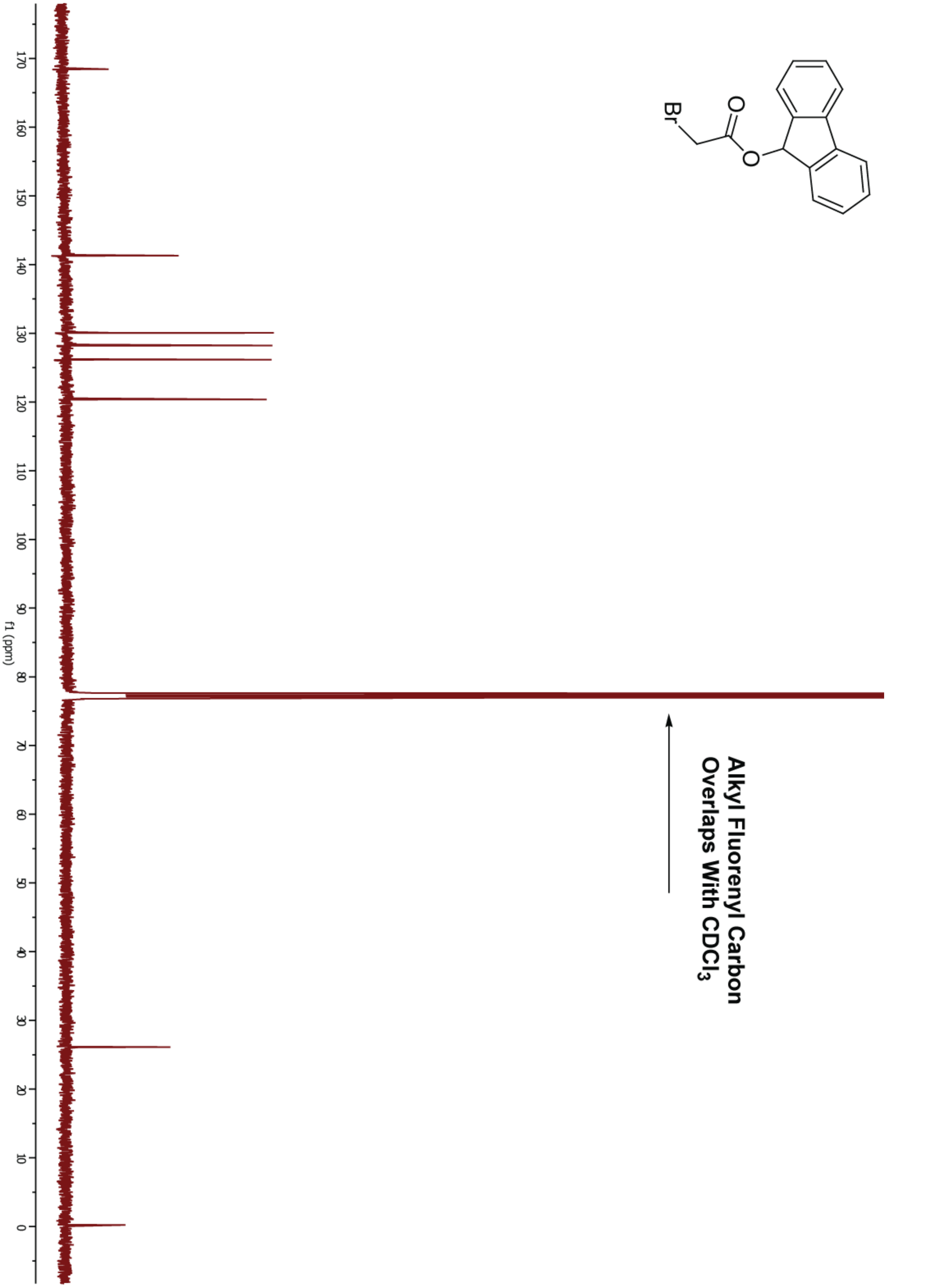
Br
O
O
Alkyl Fluorenyl Carbon Overlaps With CDCl3
f1 (ppm)
170
160
150
140
130
120
110
100
90
80
70
60
50
40
30
20
10
0

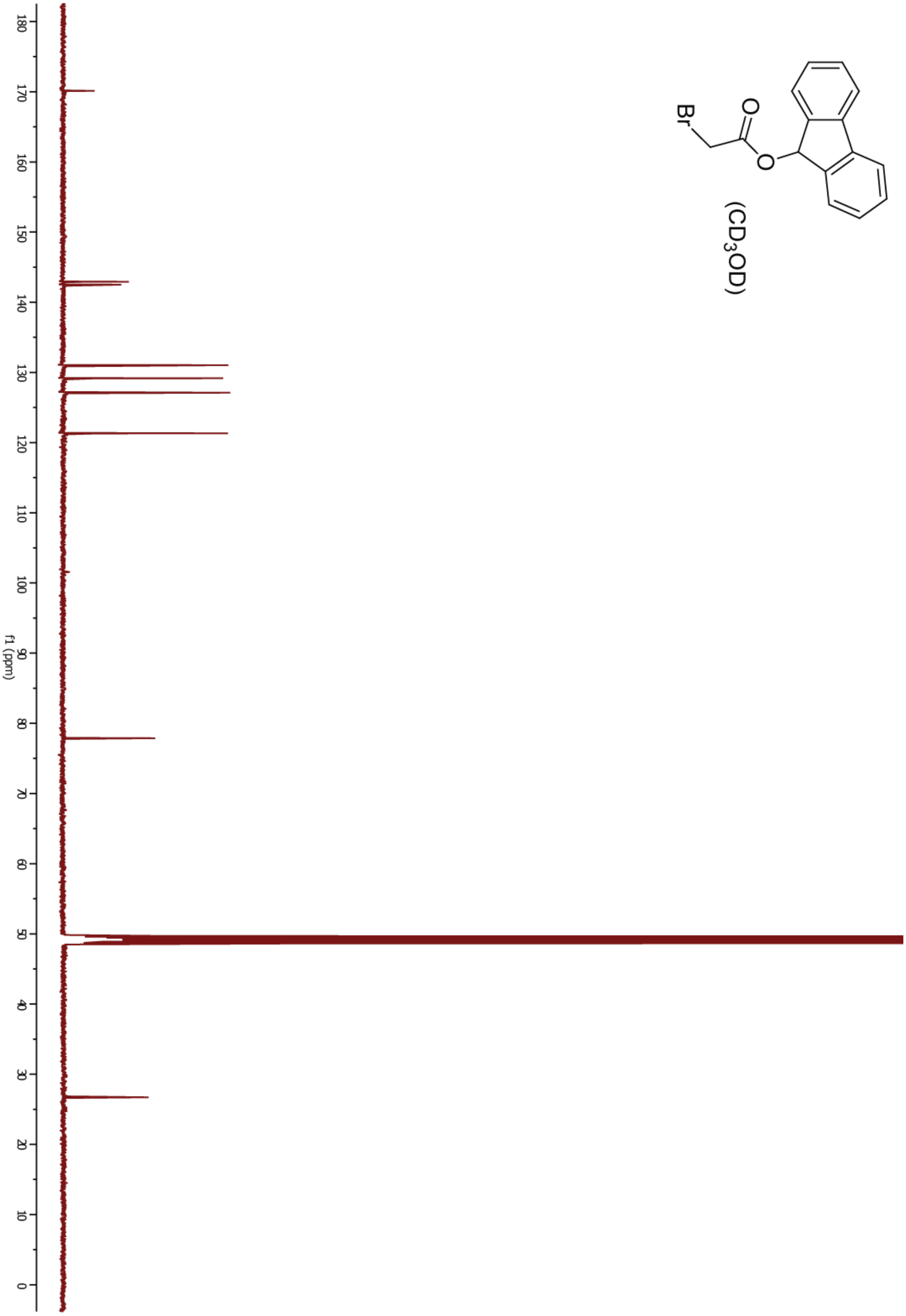
Br
O
O
(CD3OD)
f1 (ppm)
180
170
160
150
140
130
120
110
100
90
80
70
60
50
40
30
20
10
0

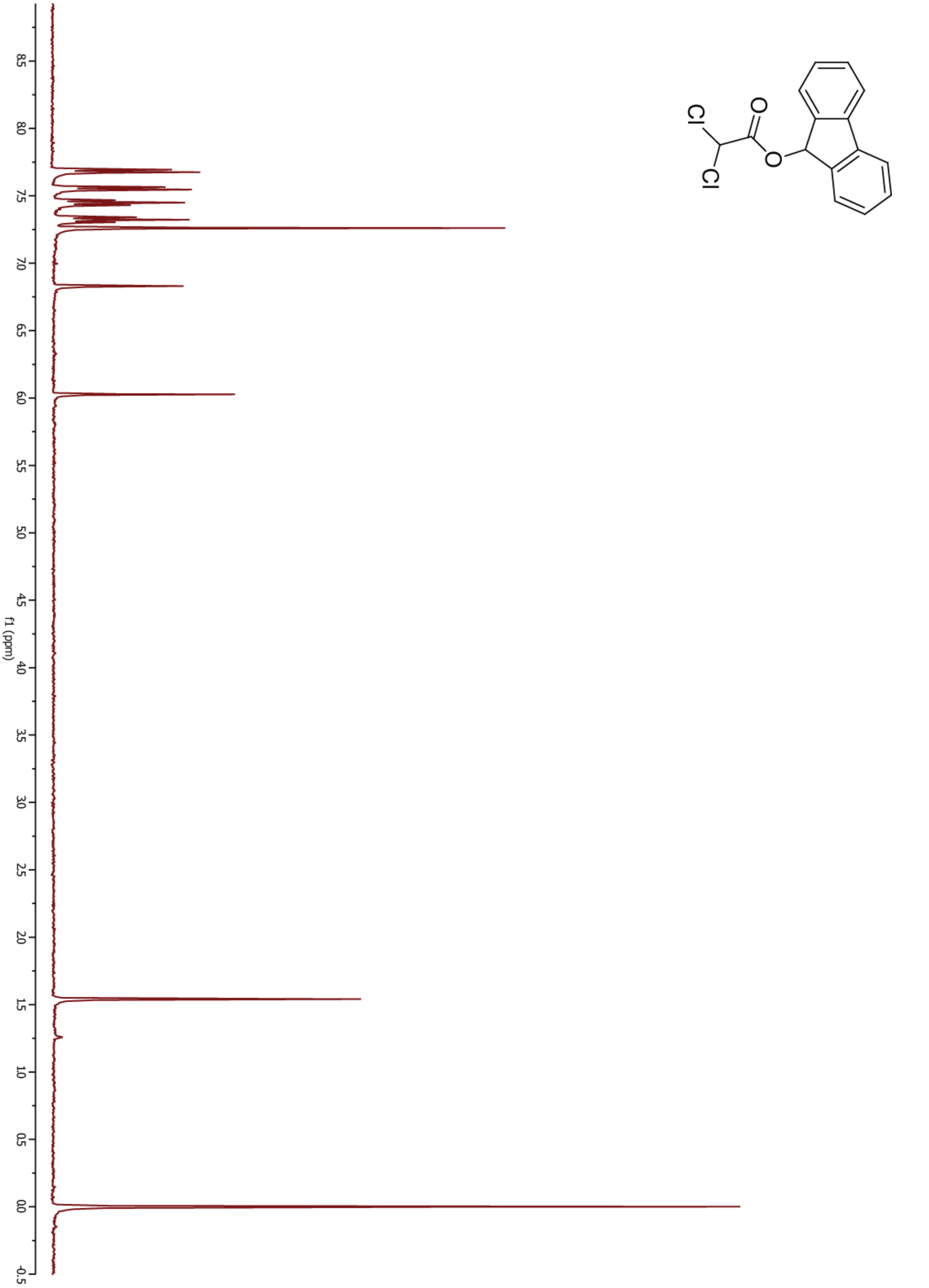
f1 (ppm)

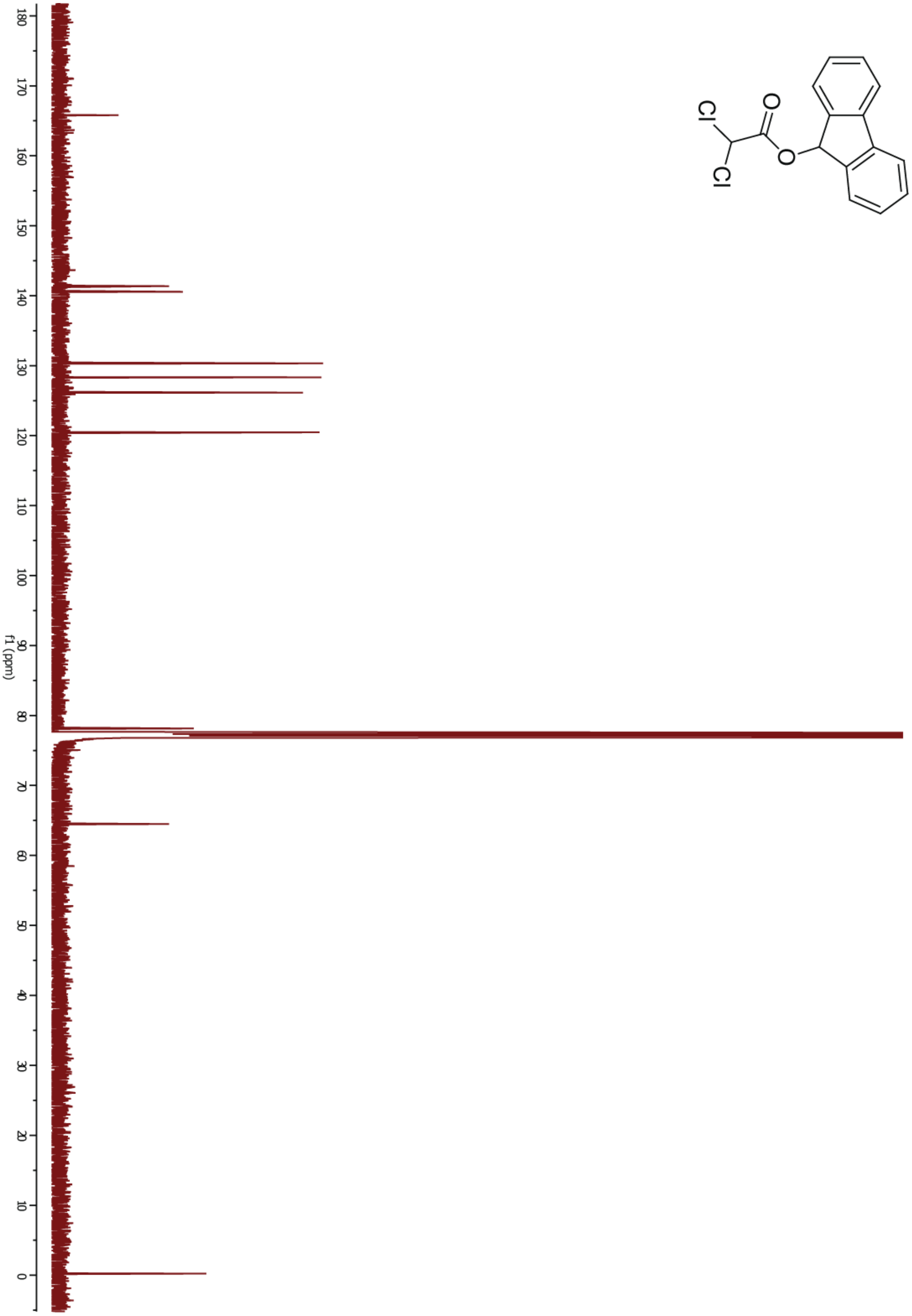
f1 (ppm)
Cl
Cl
O
O

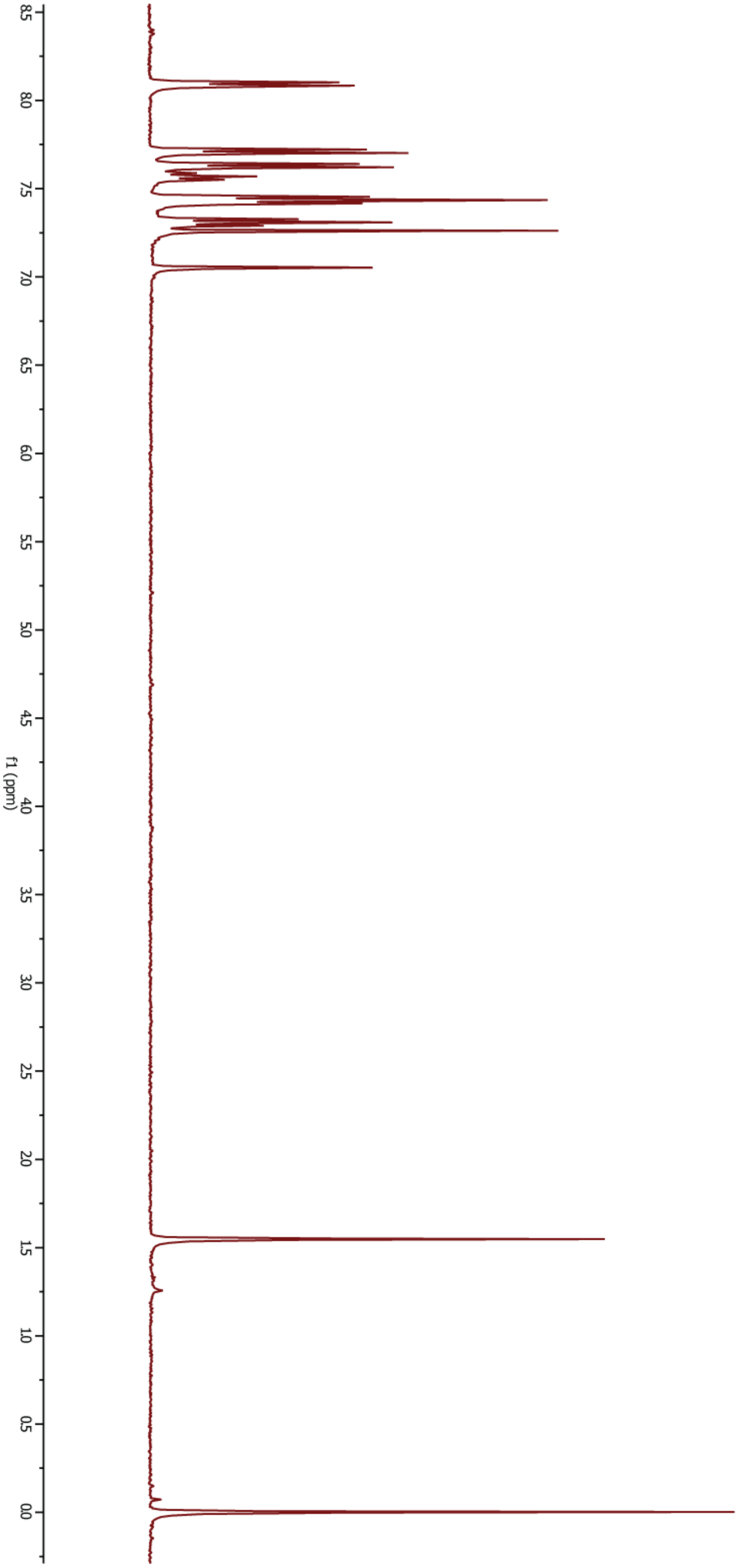
8.5
8.0
7.5
7.0
6.5
6.0
5.5
5.0
4.5
4.0
f1 (ppm)
3.5
3.0
2.5
2.0
1.5
1.0
0.5
0.0

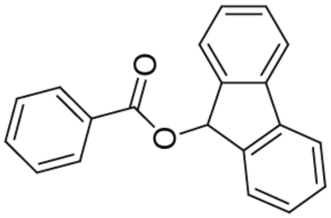

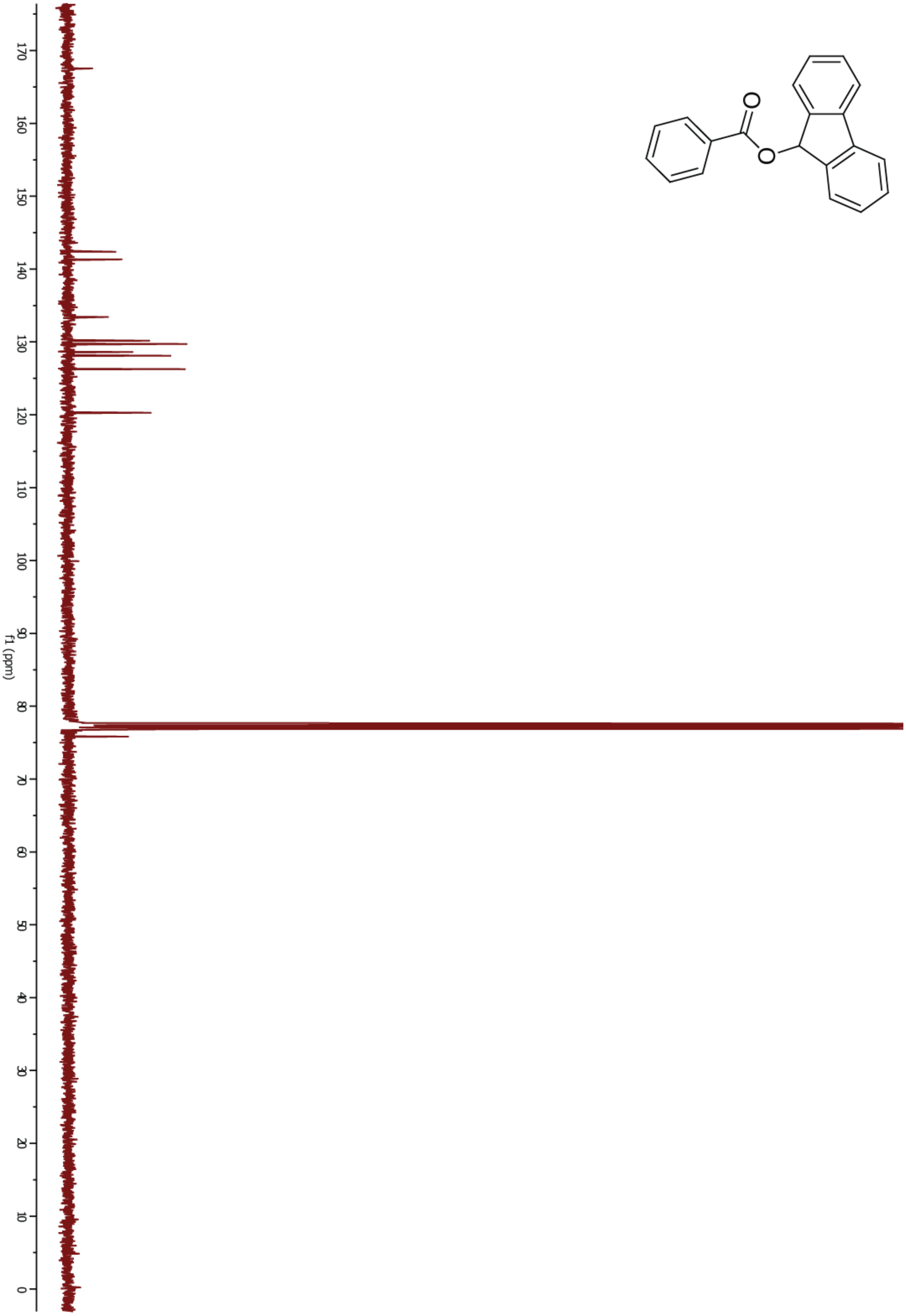
170
160
150
140
130
120
110
100
90
f1 (ppm)
80
70
60
50
40
30
20
10
0

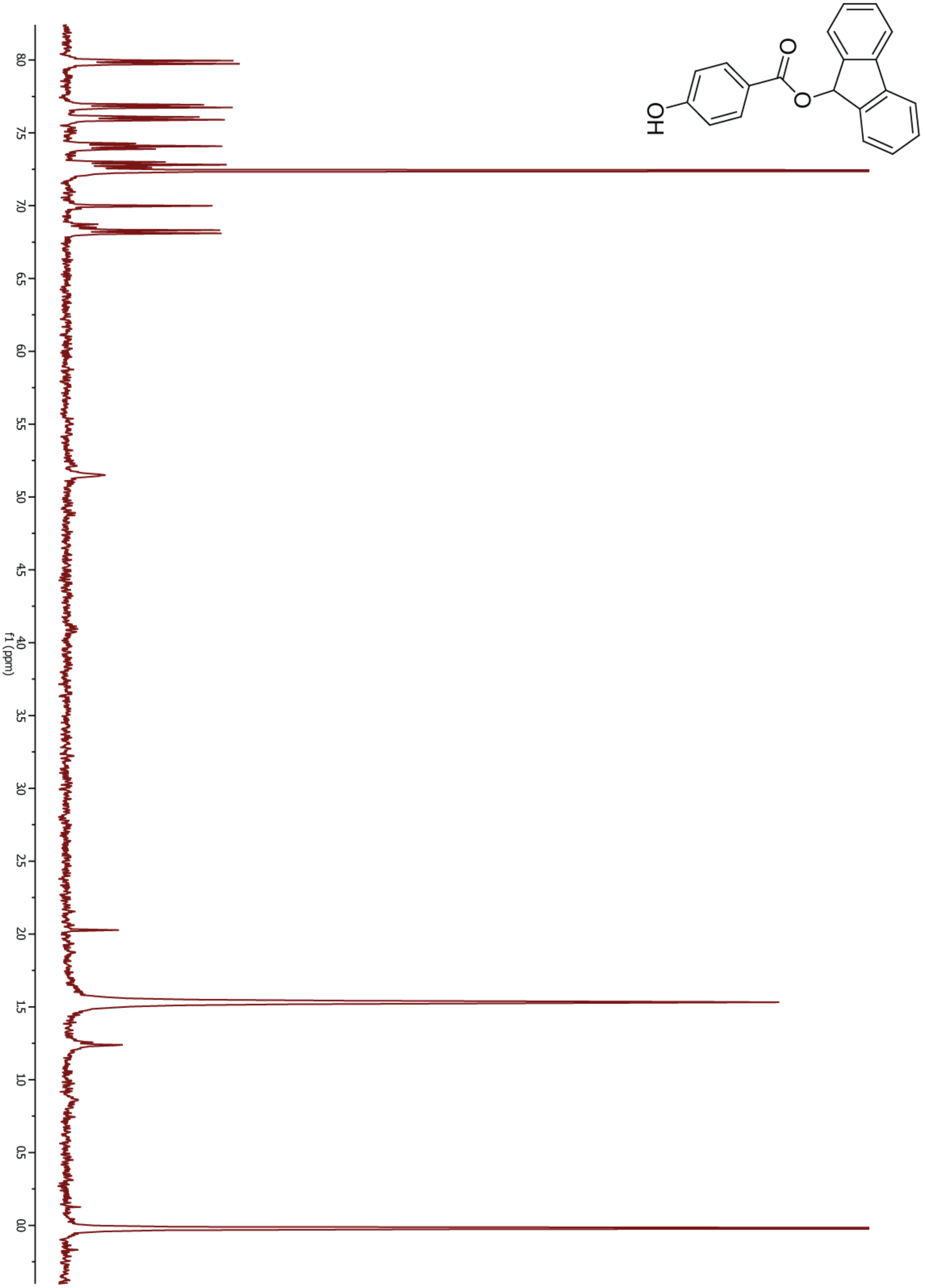
f1 (ppm)
HO

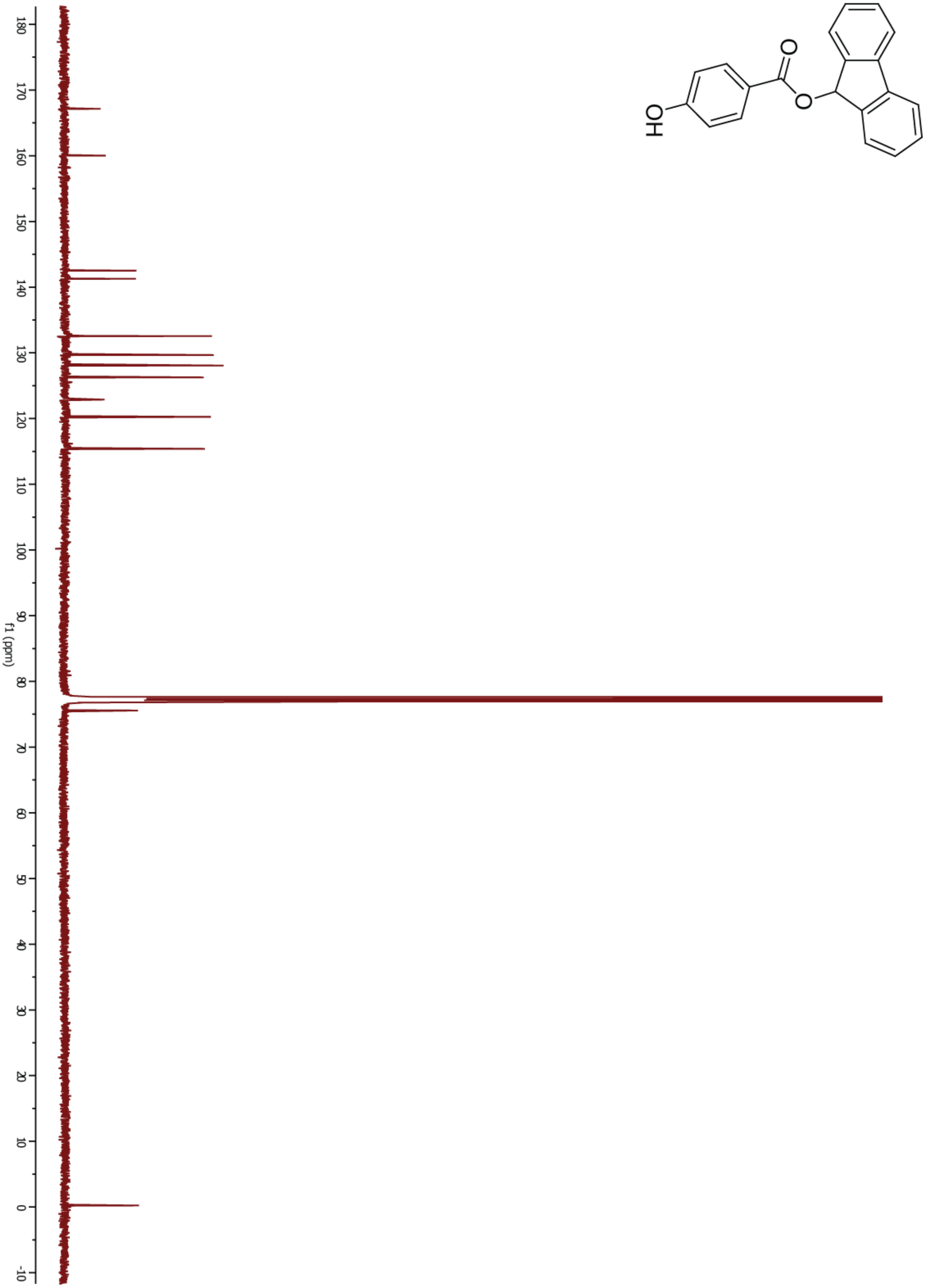
HO
O
O
f1 (ppm)
180
170
160
150
140
130
120
110
100
90
80
70
60
50
40
30
20
10
0
-10

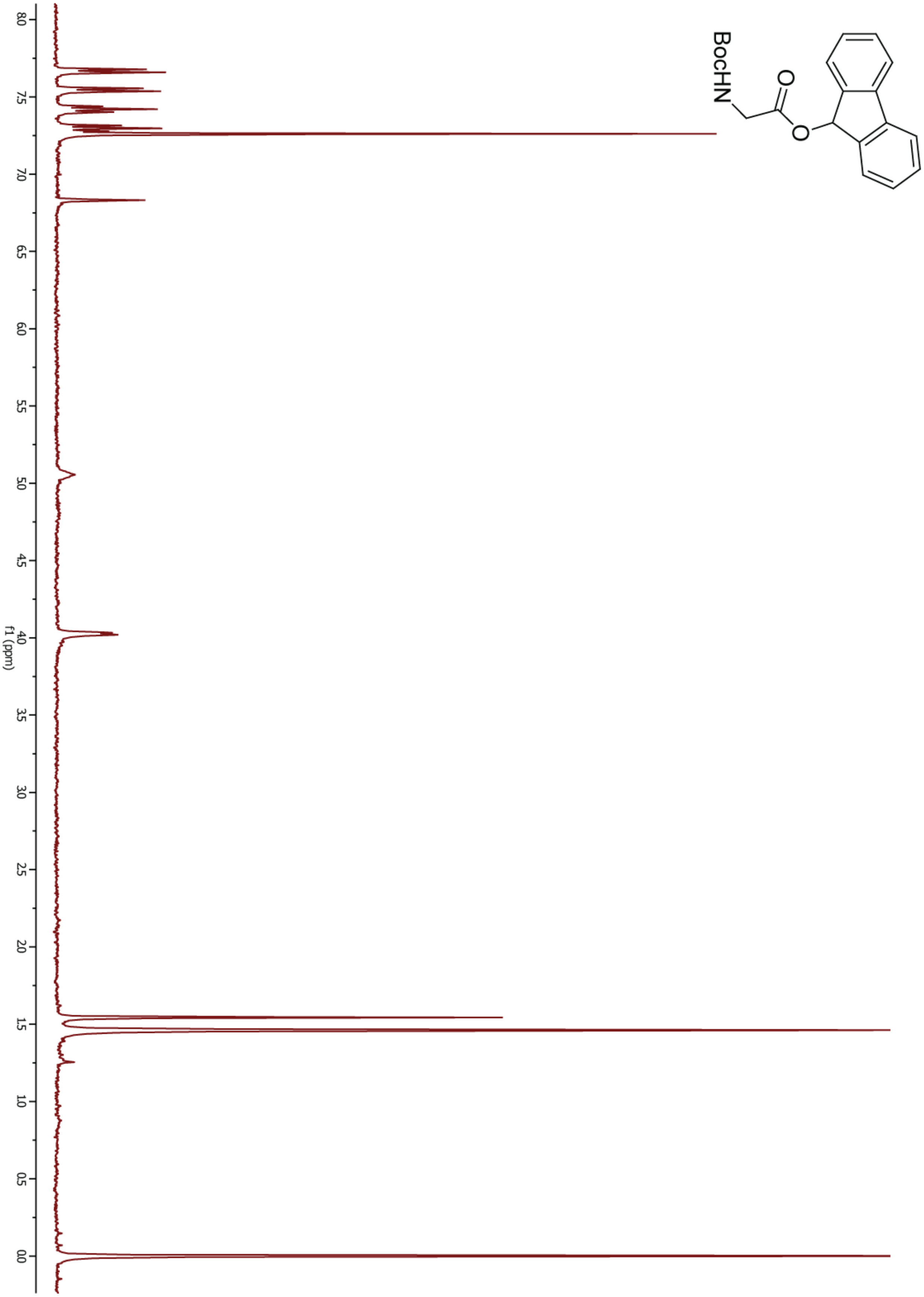
BocHN
O
O
f1 (ppm)

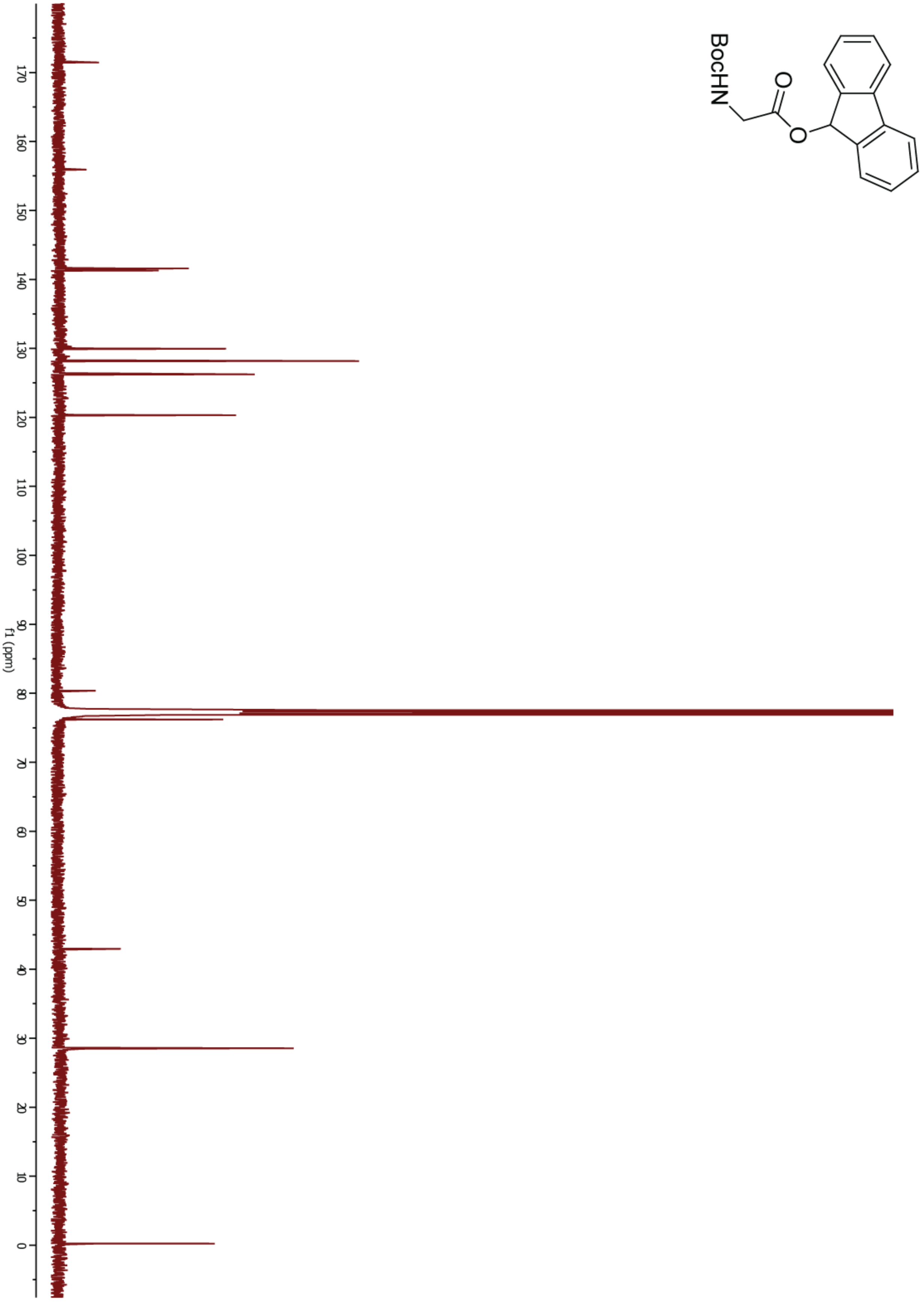
BocHN
O
O
f1 (ppm)
170
160
150
140
130
120
110
100
90
80
70
60
50
40
30
20
10
0

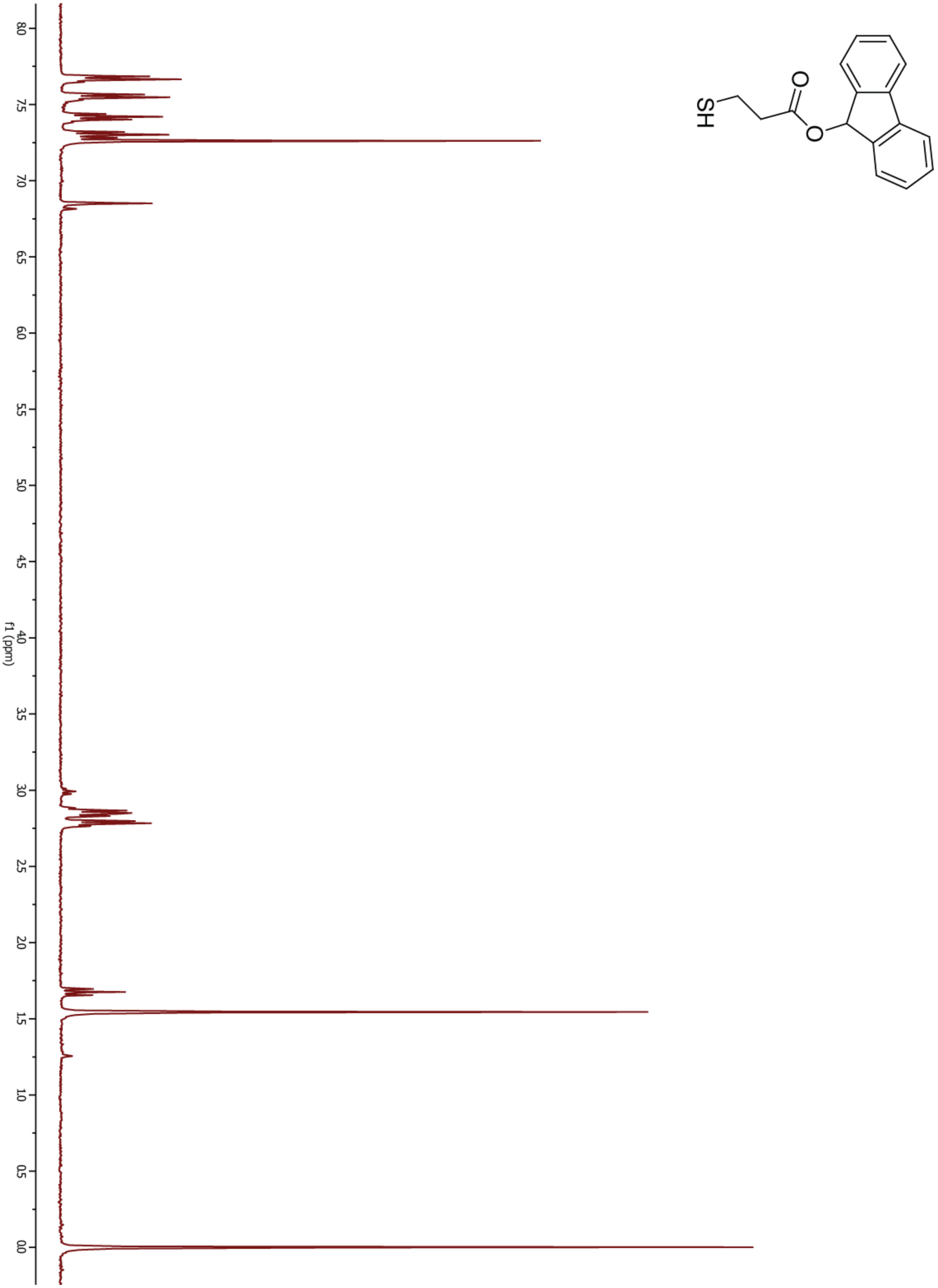
SH
O
O
f1 (ppm)

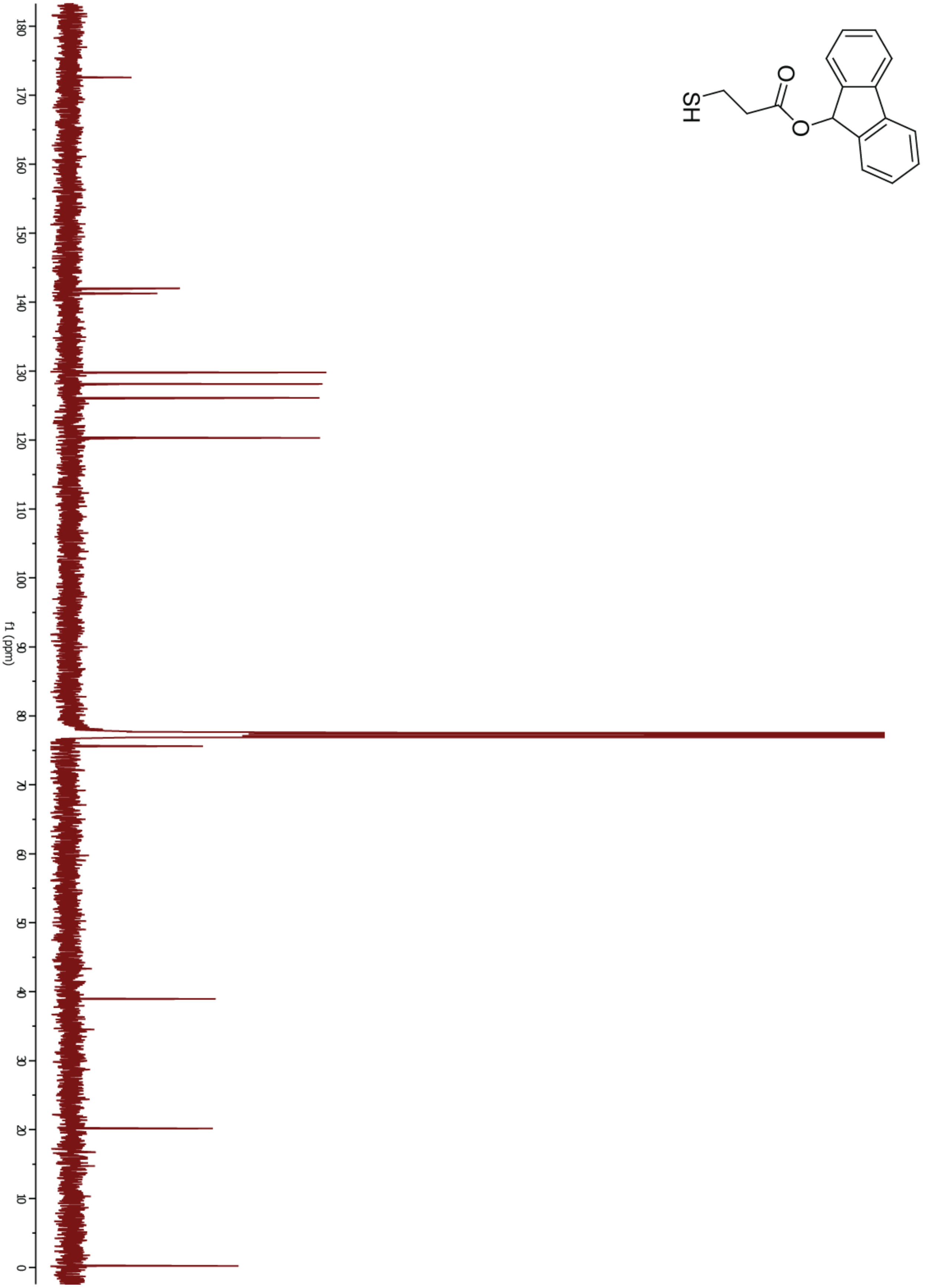
SH
O
O
f1 (ppm)
180
170
160
150
140
130
120
110
100
90
80
70
60
50
40
30
20
10
0

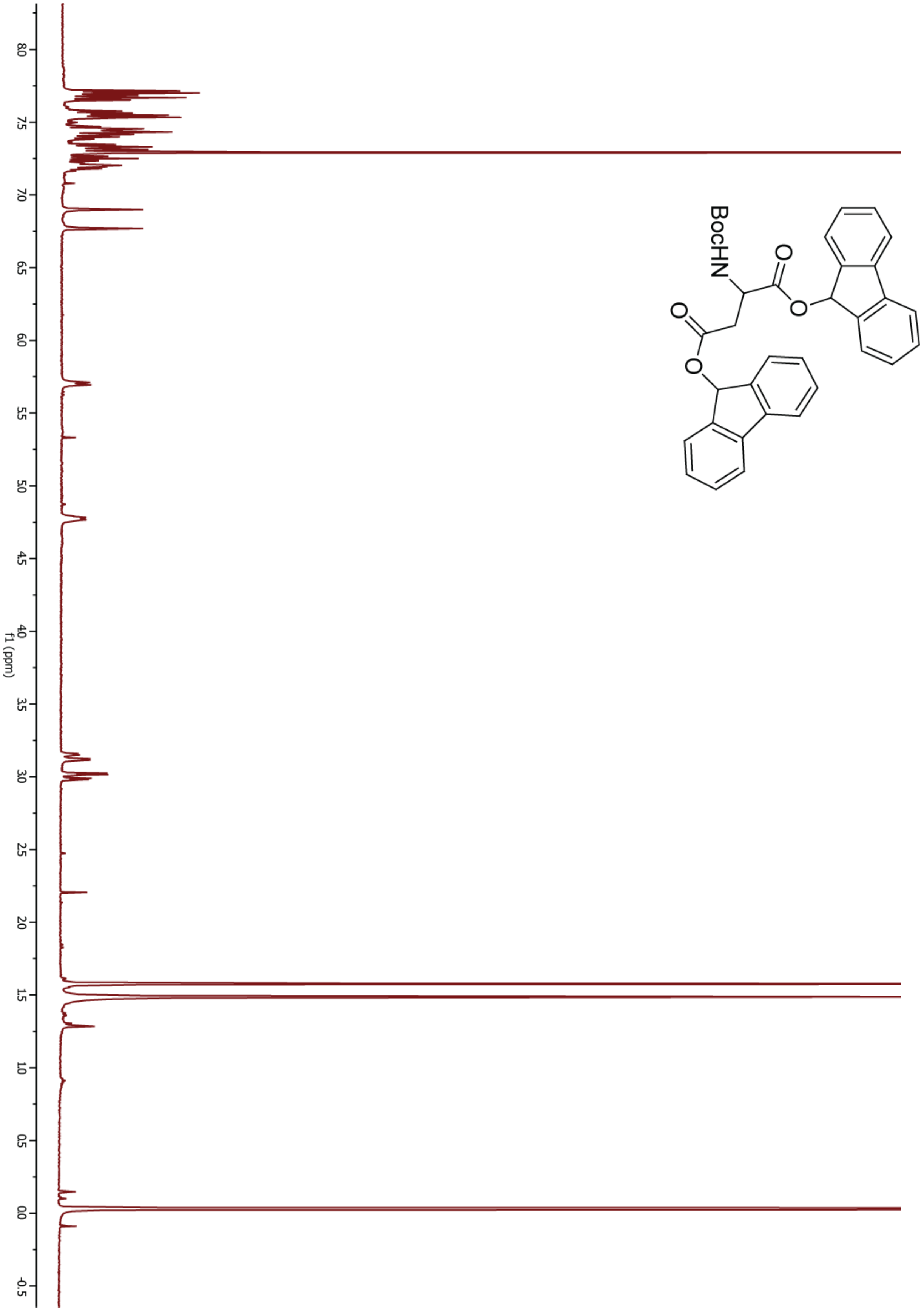
BocHN
O
O
O
O
8.0
7.5
7.0
6.5
6.0
5.5
5.0
4.5
4.0
3.5
3.0
2.5
2.0
1.5
1.0
0.5
0.0
-0.5
f1 (ppm)

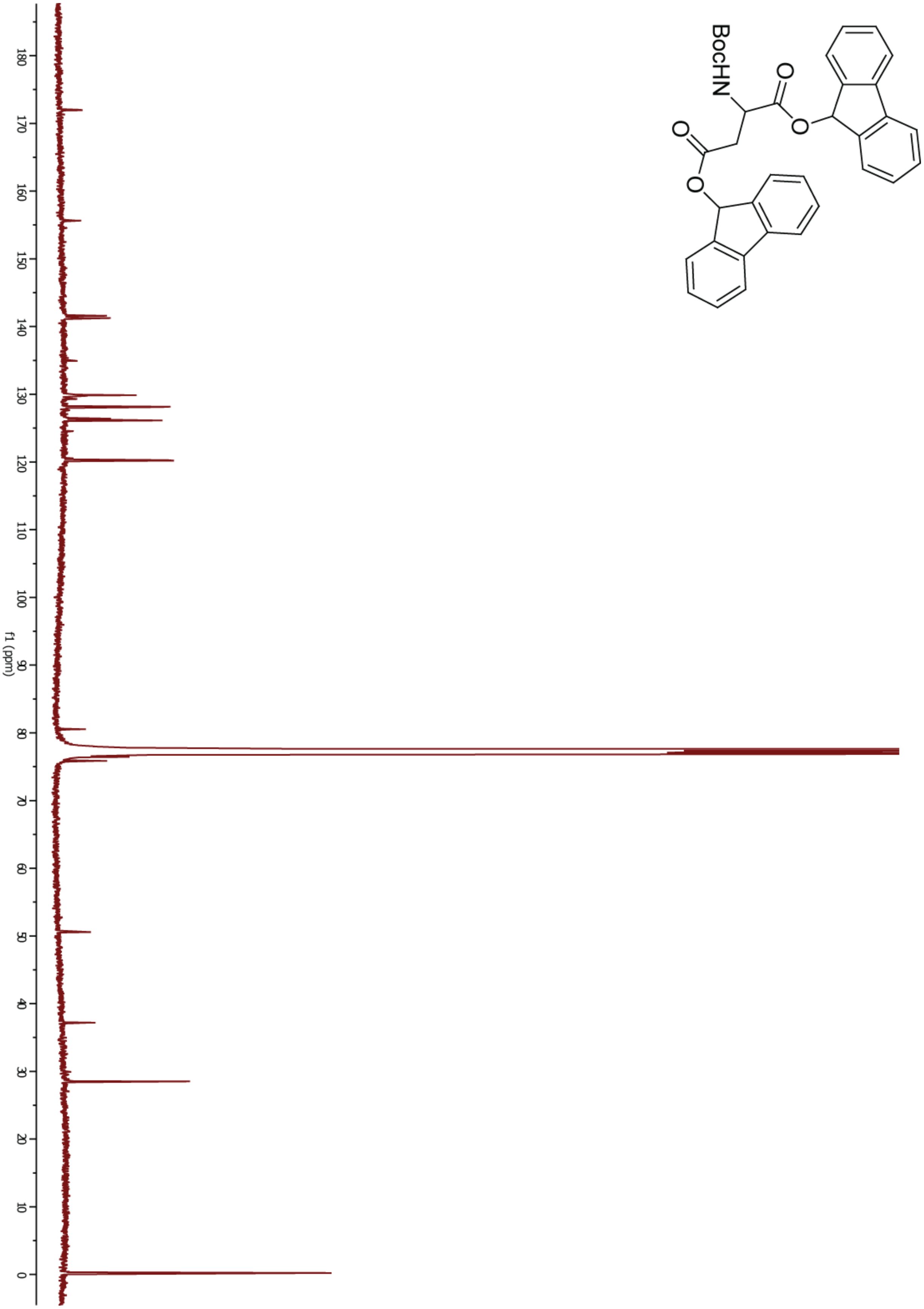
BocHN
O
O
O
O
180
170
160
150
140
130
120
110
100
90
80
70
60
50
40
30
20
10
0
f1 (ppm)

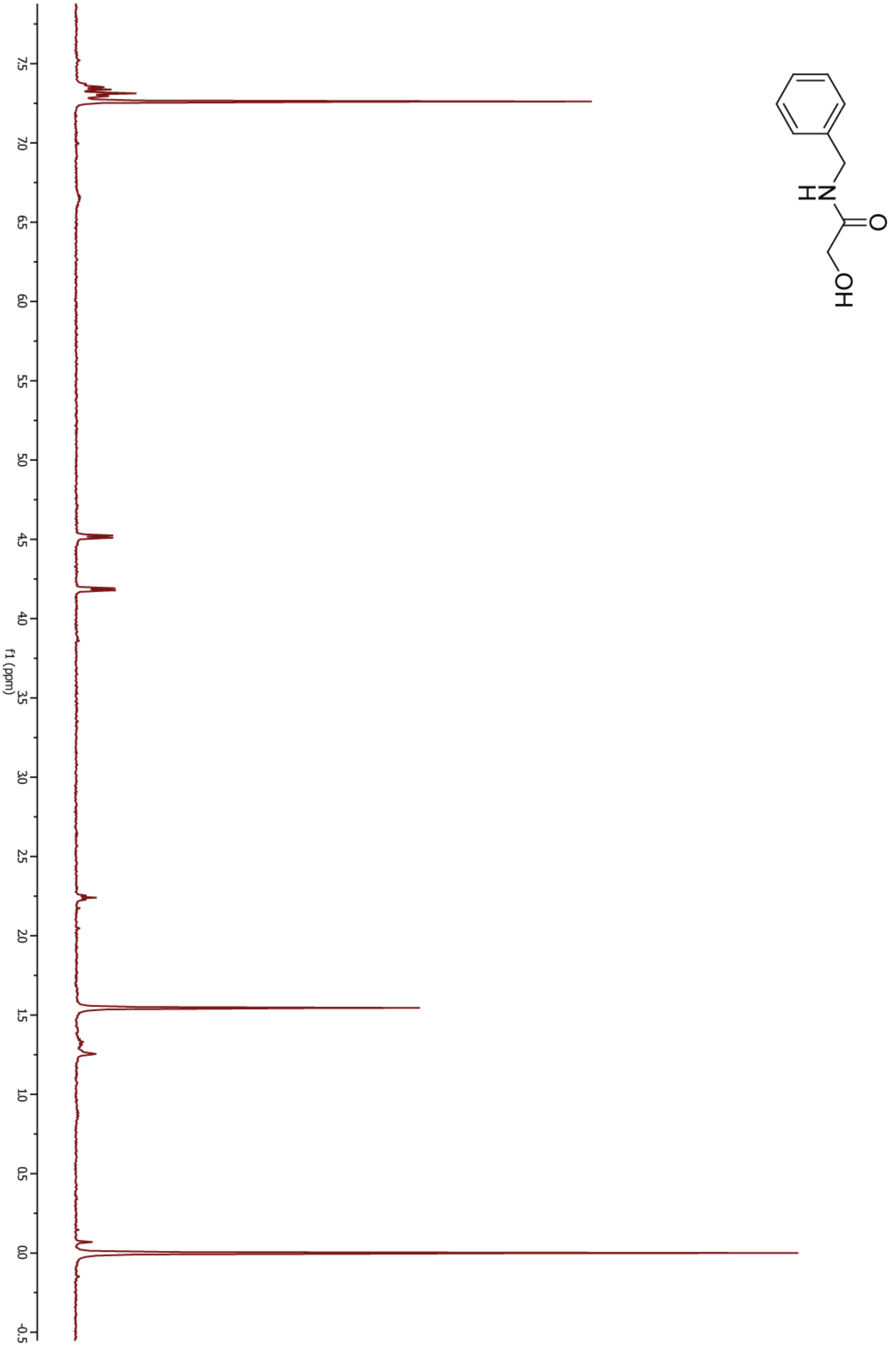
7.5
7.0
6.5
6.0
5.5
5.0
4.5
4.0
3.5
3.0
2.5
2.0
1.5
1.0
0.5
0.0
-0.5
f1 (ppm)
N
H
O
OH

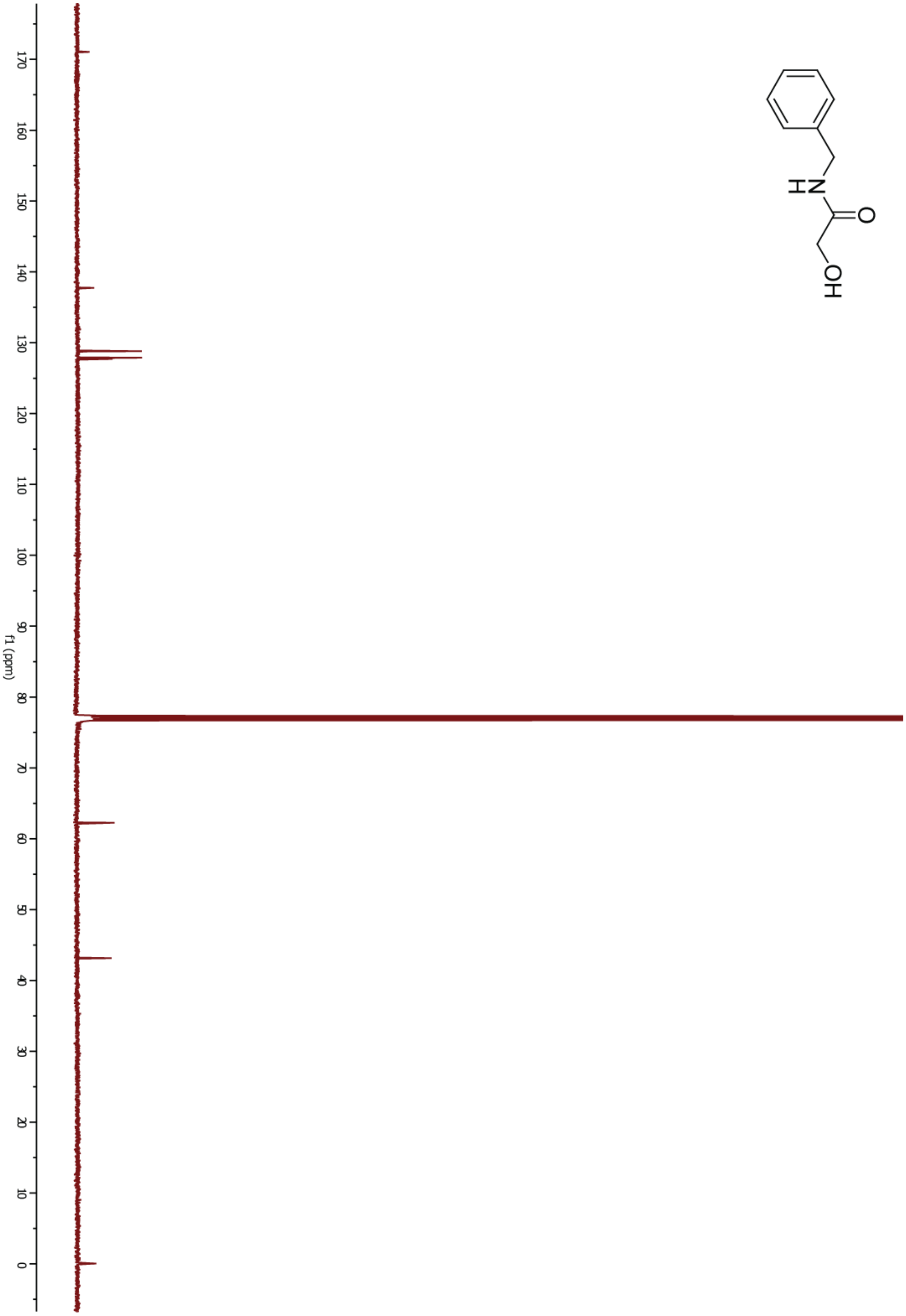
170
160
150
140
130
120
110
100
90
f1 (ppm)
80
70
60
50
40
30
20
10
0
H
N
O
OH

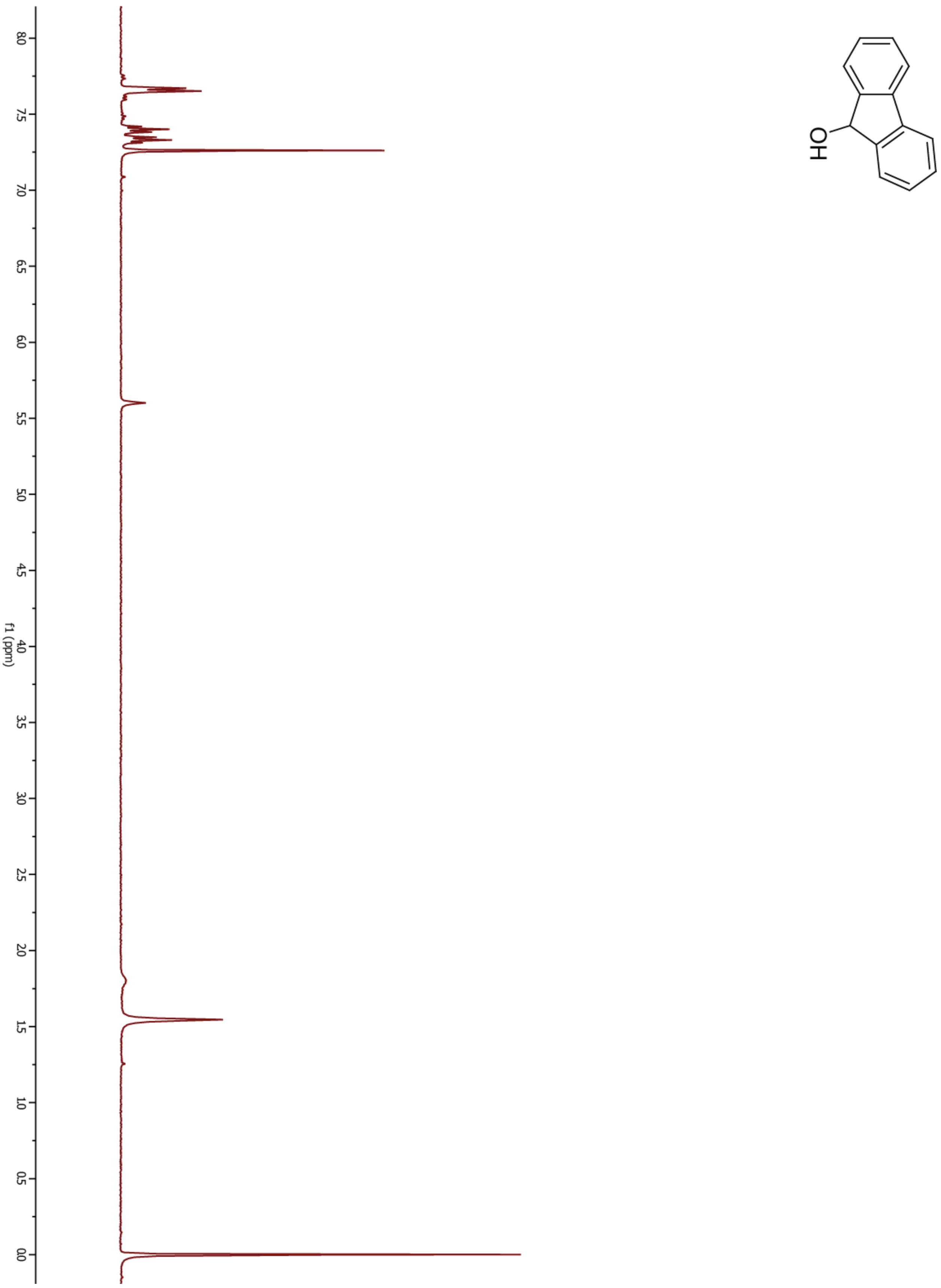
OH
f1 (ppm)
8.0
7.5
7.0
6.5
6.0
5.5
5.0
4.5
4.0
3.5
3.0
2.5
2.0
1.5
1.0
0.5
0.0

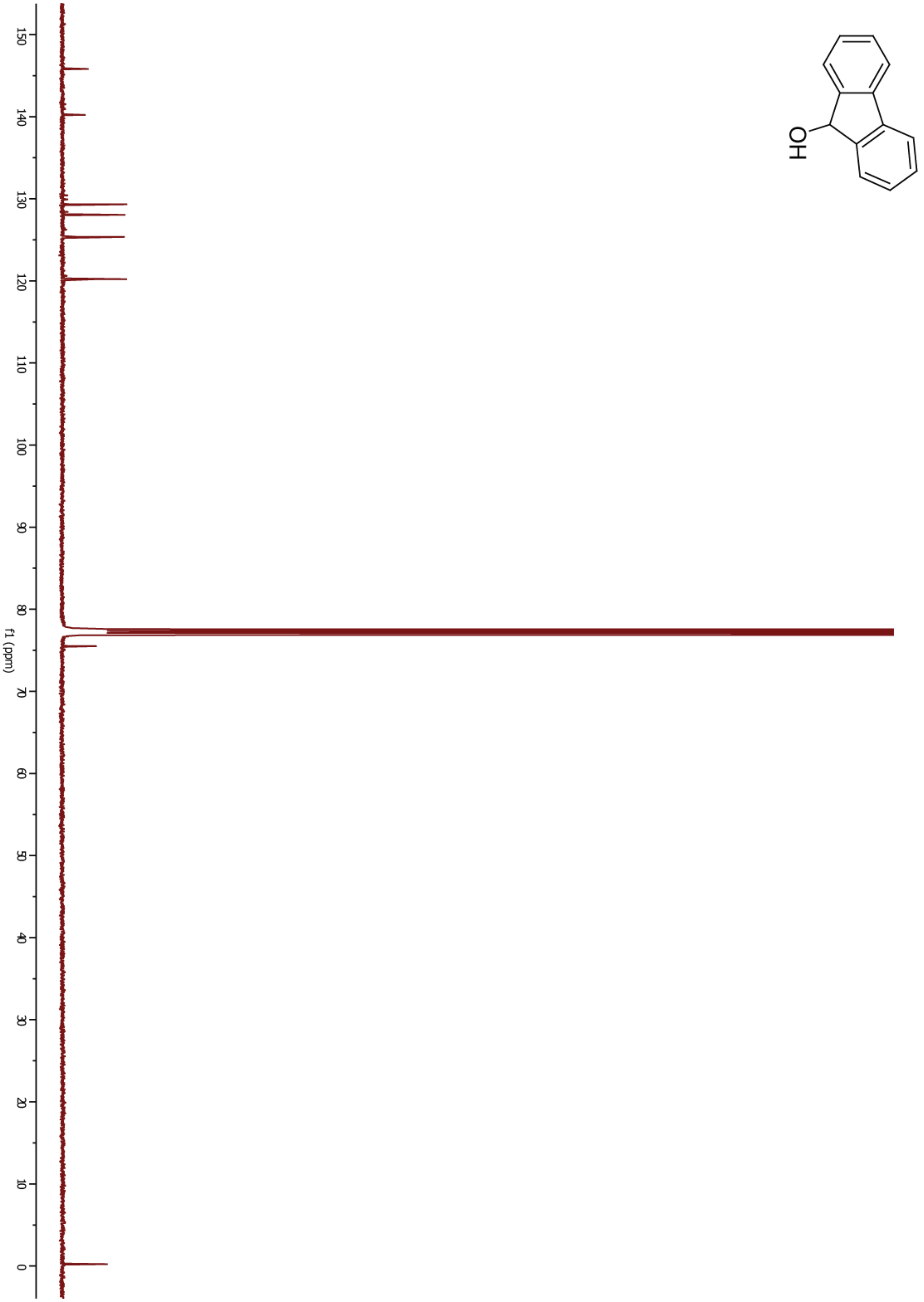
OH
f1 (ppm)
150
140
130
120
110
100
90
80
70
60
50
40
30
20
10
0

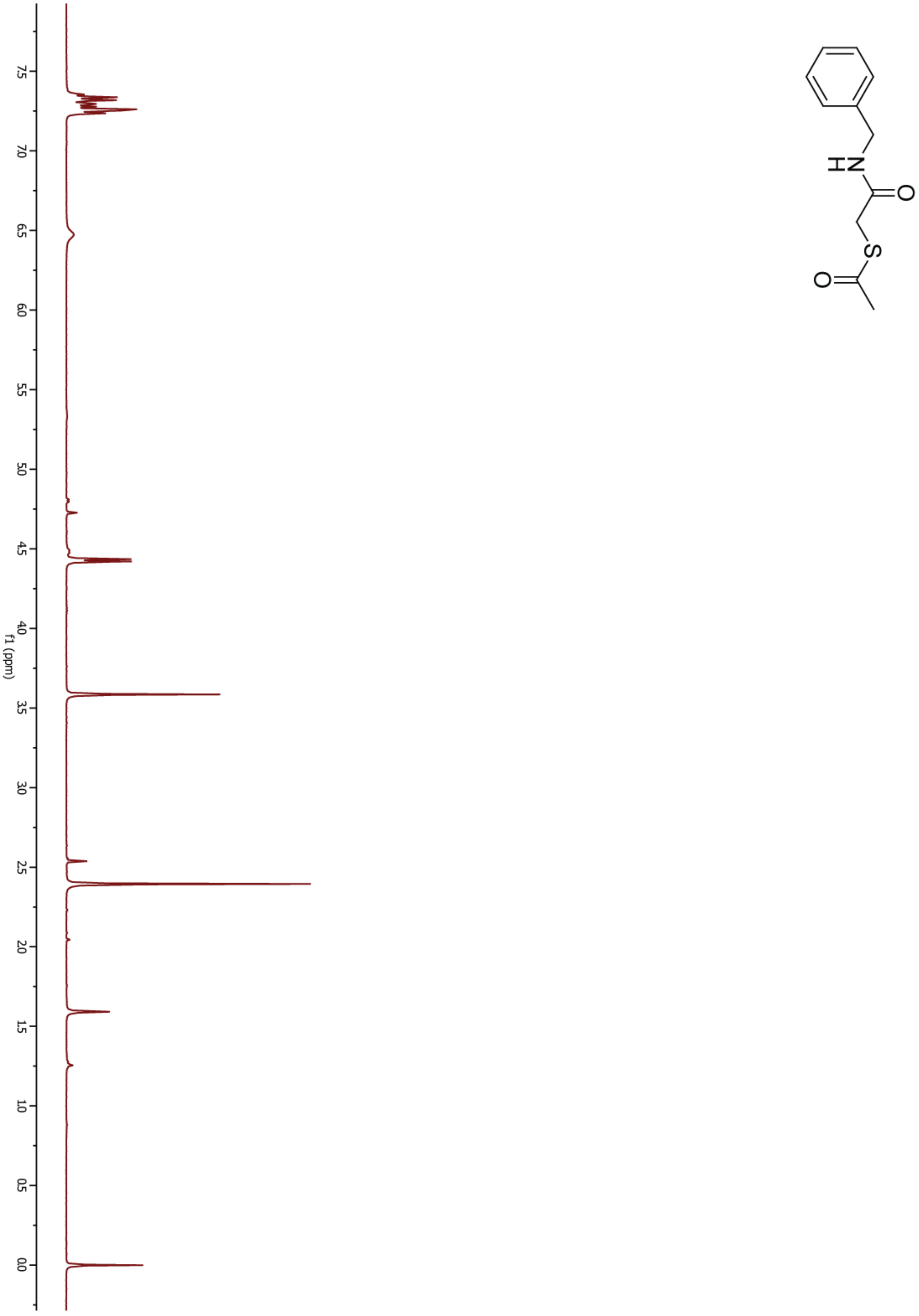
f1 (ppm)

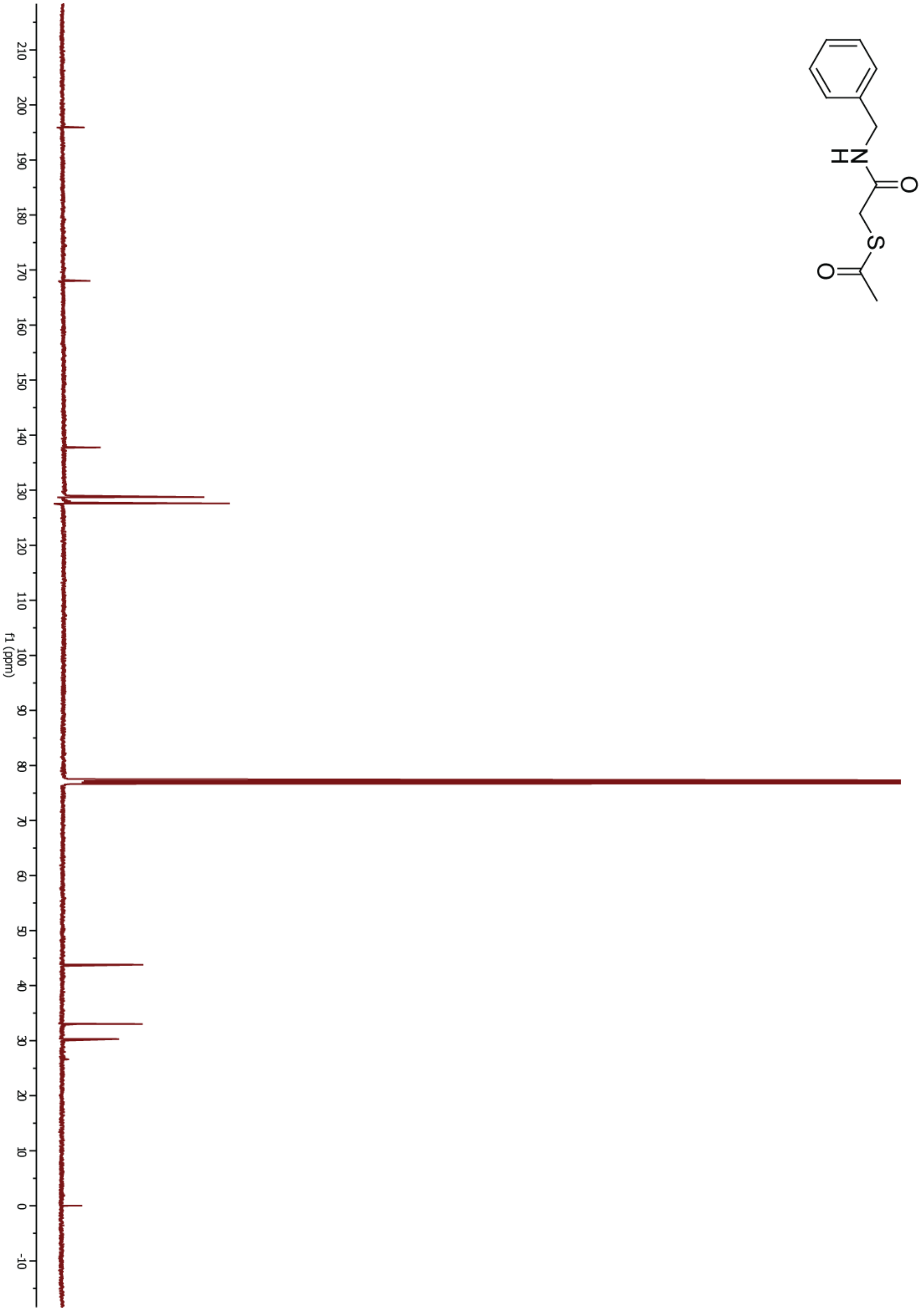
f1 (ppm)
210
200
190
180
170
160
150
140
130
120
110
100
90
80
70
60
50
40
30
20
10
0
-10
N
H
O
S
O

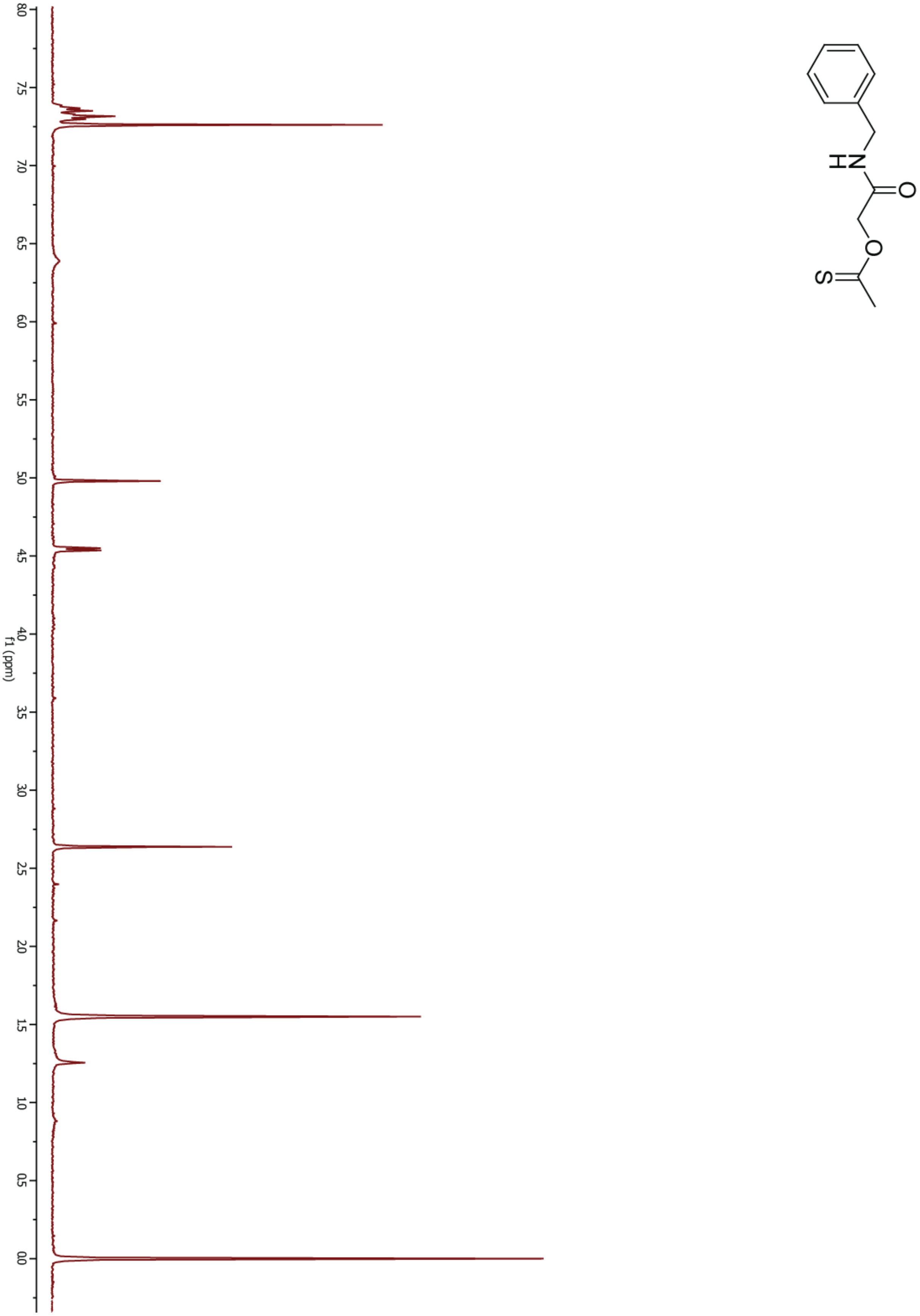
f1 (ppm)

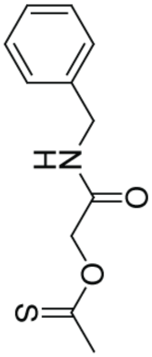

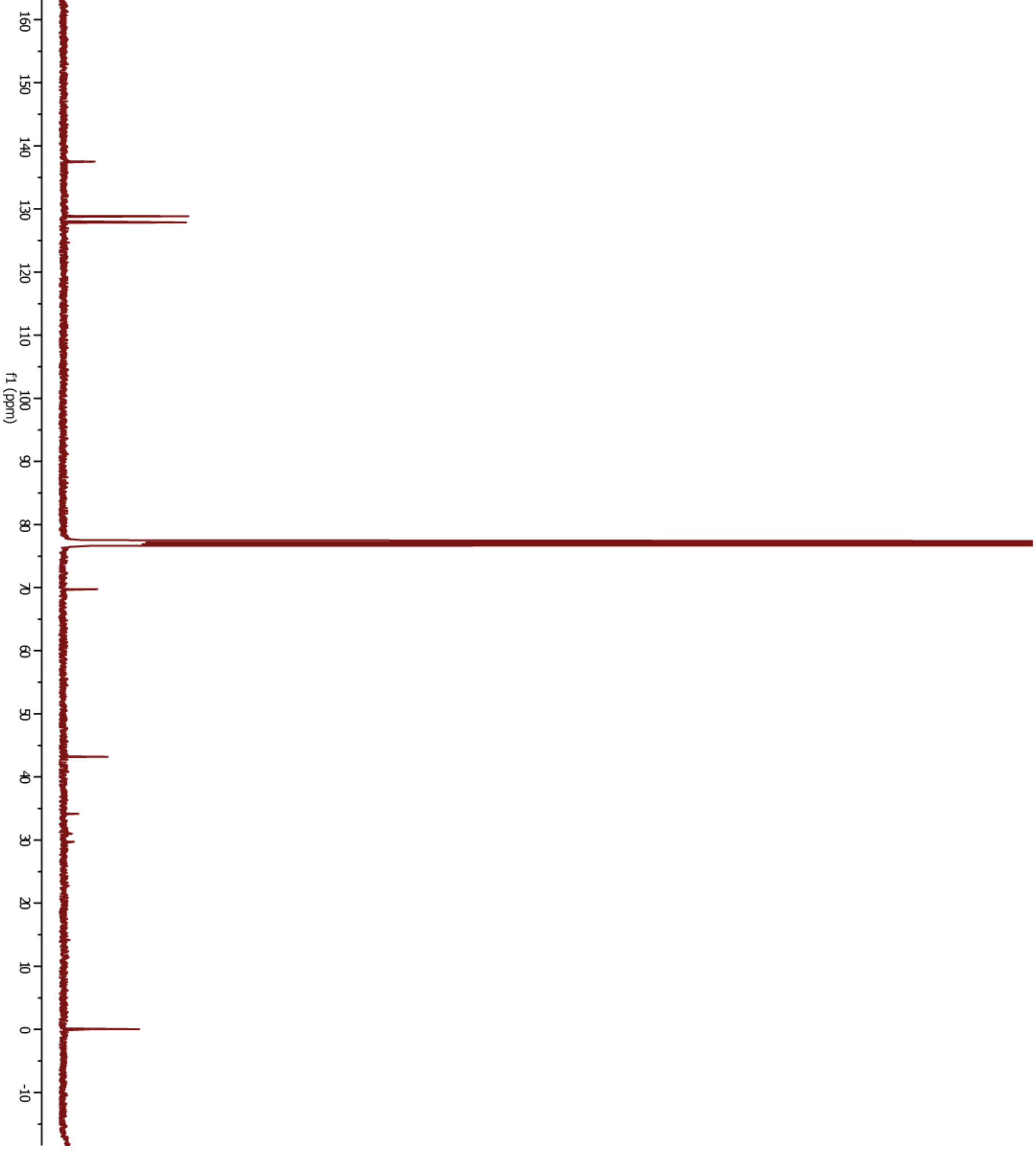
f1 (ppm)

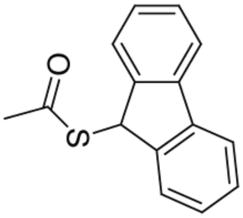
O
S

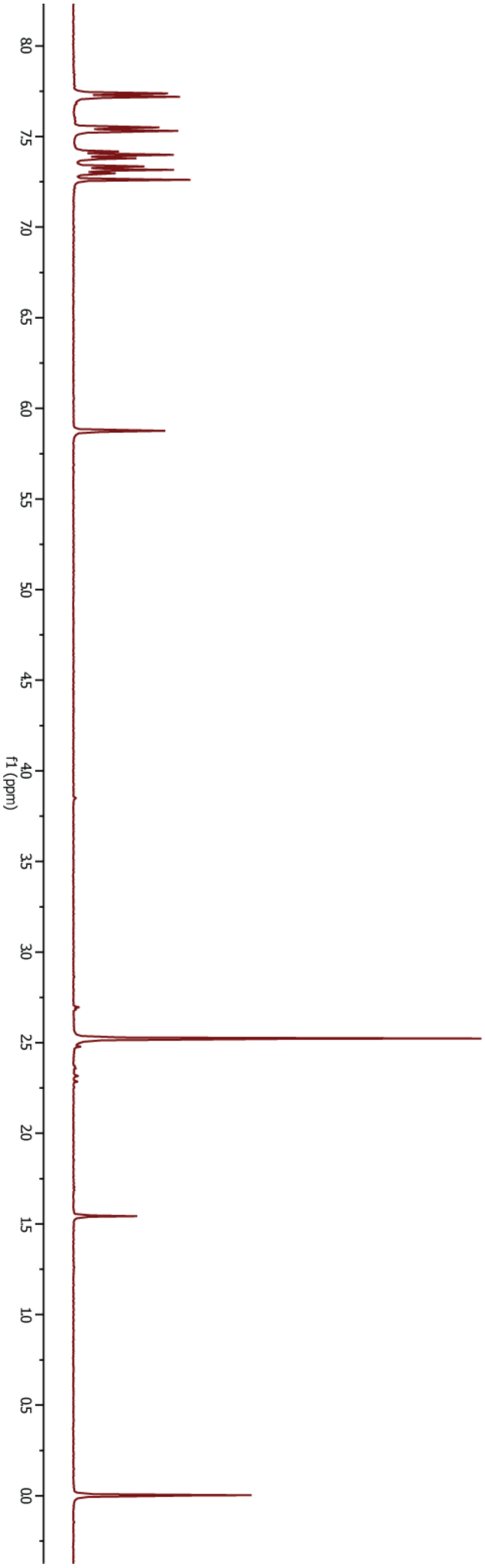
8.0
7.5
7.0
6.5
6.0
5.5
5.0
4.5
4.0
f1 (ppm)
3.5
3.0
2.5
2.0
1.5
1.0
0.5
0.0

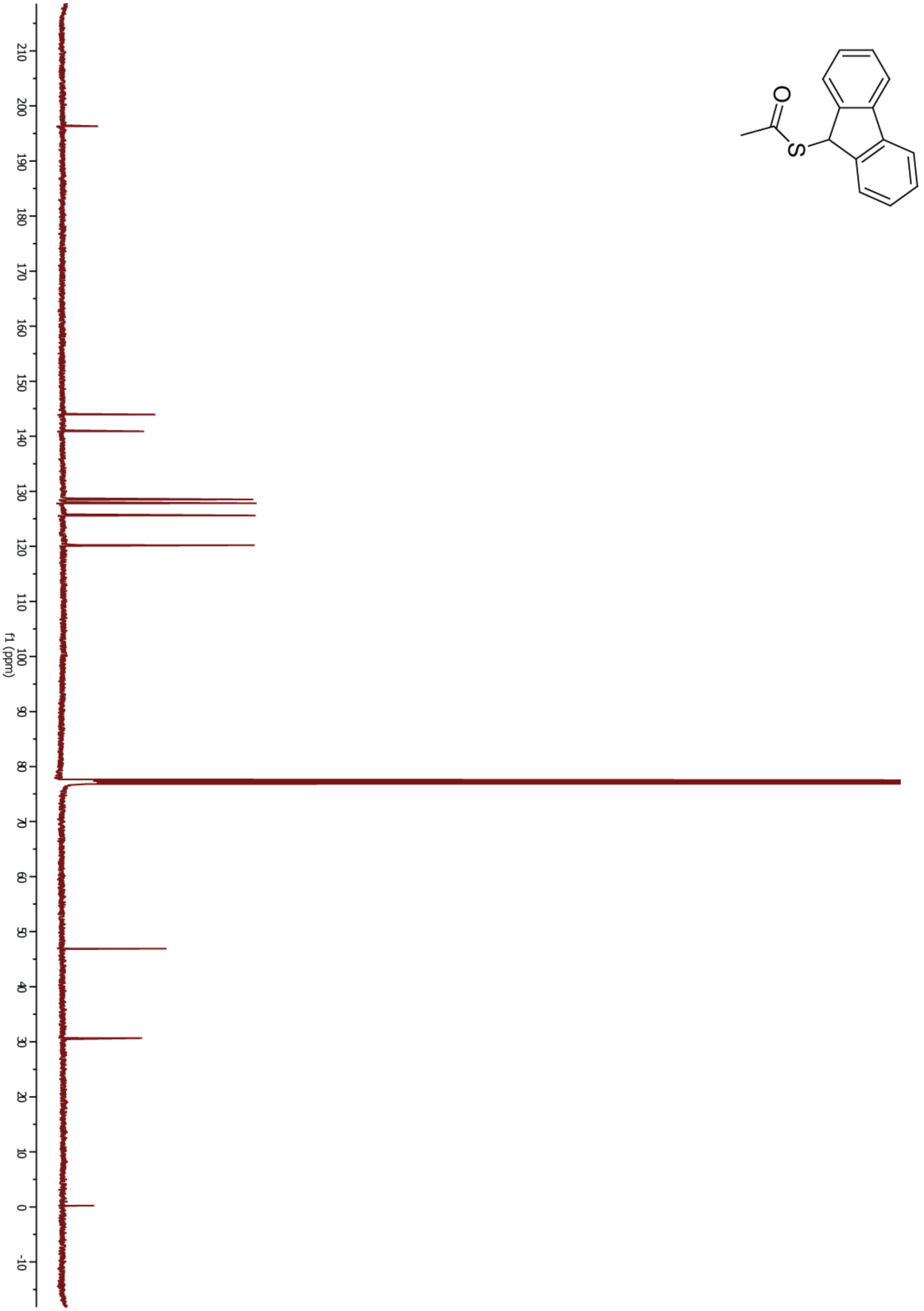
O
S
f1 (ppm)
210
200
190
180
170
160
150
140
130
120
110
100
90
80
70
60
50
40
30
20
10
0
-10
